# The Evolution of Polyclonal Competition in Aging Hematopoiesis

**DOI:** 10.1158/2159-8290.CD-25-0990

**Published:** 2026-05-05

**Authors:** Nathaniel V. Mon Père, Francesco Terenzi, Benjamin Werner

**Affiliations:** Evolutionary Dynamics Group, Centre for Cancer Evolution, Barts Cancer Institute, Queen Mary University of London, London, United Kingdom.

## Abstract

Clonal hematopoiesis (CH)—the expansion of genetic variants in blood—is a prime example of somatic evolution. Although it often precedes malignant transformation, many aspects of this process remain unknown. We show that a model of polyclonal competition, in which selectively advantaged clones continually occur and compete, explains observed CH dynamics throughout human life. We quantify the fitness distribution and occurrence rate of clonal expansions using either variant trajectories or hematopoietic stem cell (HSC) genetic heterogeneity. Inferences on both data converge. Approximately 3 fit clones enter the HSC pool per year, yet rarely more than 5 achieve >1.5% frequency throughout life. The fittest clones emerge predominantly later in life in accordance with a multistep evolutionary process. *DNMT3A* variants were enriched for single-hit clones, whereas *TET2*, *ASXL1*, *JAK2*, *SF3B1*, and *SRSF2* showed enrichment for multihit evolution. These findings suggest that precursors of hematologic malignancies are identifiable prior to transformation and may facilitate early intervention strategies.

**Significance:**

We study the evolution of CH through longitudinal variant trajectories and HSC genetic heterogeneity. Clonal interference and a multistep evolutionary process become evident. Clones further advanced on the path to malignant transformation are identifiable, and propensities for multiple hits differ among most commonly mutated CH variants.

## Teaser

An evolutionary model of clonal hematopoiesis (CH) describes the genetic changes of HSCs through life, predicts the ubiquity of polyclonal competition, and identifies multistage CH progression with varying propensity amongst CH genes.

## Introduction

Genetic heterogeneity pervades somatic tissues at all stages of human life (1–5). It emerges from ubiquitous mutational processes and subsequent expansion of novel clones by selection and random drift (6–10). This phenomenon has been well characterized in blood, where largely expanded clones are common late in life, a condition termed clonal hematopoiesis (CH; refs. 11–16). CH is associated with various adverse outcomes in the aging human population (11), including increased risks of lineage-specific hematologic malignancies (17, 18), atherosclerotic cardiovascular disease (19), chronic liver disease (20), and lung cancer (21, 22), as well as a higher rate of death in patients with solid tumors (23). It is transferable by bone marrow (BM) transplant—persisting and evolving in transplant recipients (24, 25)—and is more common in smokers and after radiation or chemotherapy (23, 26), strongly suggesting context-dependent natural selection. Interestingly, despite the increased cancer risk, the majority of CH cases still do not progress to hematologic malignancies. This dichotomy is also observed in other human tissues. Aging skin and esophagus are riddled with cancer-associated genetic variants (3, 27) without progressing, whereas some variants—most prominently *NOTCH1*—are even thought to be protective (28). In contrast to many other tissues, CH can be followed longitudinally at population scale (29). This offers a unique window into the dynamics of somatic evolution and malignant transformation in human tissues, prospectively promising a better understanding of cancer initiation and informing on strategies of prevention, screening, and early intervention (17).

Obtaining a detailed quantitative description of the evolutionary forces of CH and how these change with age, mutational load, and tissue regulation remains challenging. Current evolutionary models of CH assume that positively selected somatic variants in hematopoietic stem cells (HSC) are rare, and their selective advantages are context independent (7, 8, 30). However, recent observations suggest a more complex scenario (bioRxiv 2023.12.21.572706; refs. 5, 15, 16). Variant frequencies measured at multiple time points cease expanding despite being positively selected (15). Although by itself such behavior is consistent with a size-constrained HSC population (31, 32), other observations remain unexplained; variants typically saturate below fixation, many variant trajectories decrease late in life, and the HSC fitness seems to decrease with age (15, 16). Stratifying interindividually further complicates this picture, as observable growth rates of identical genetic variants vary greatly across donors and age (7, 15, 30, 33).

**Figure 1.**
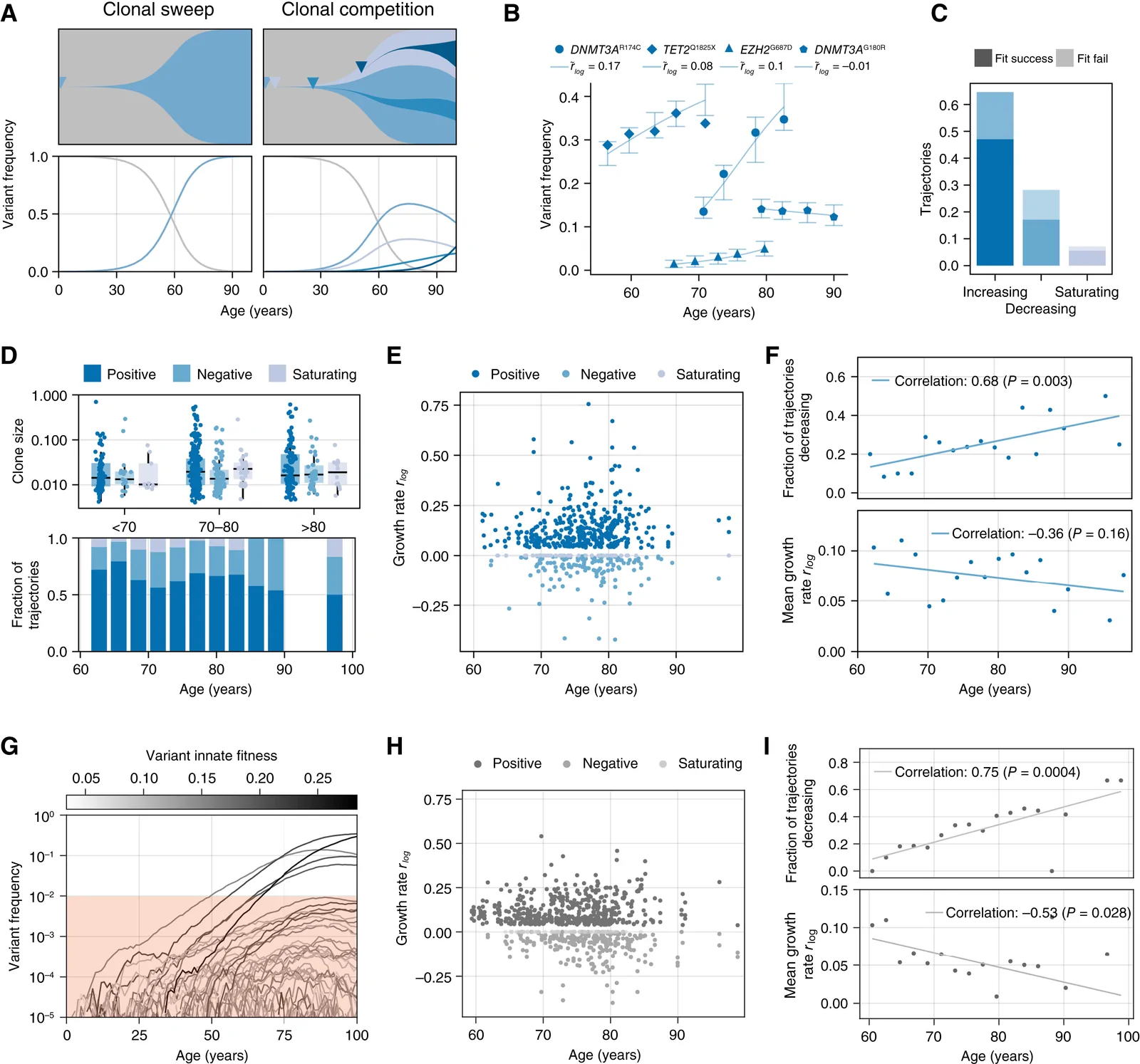
Changing fitness landscape of human hematopoiesis with age. **A,** Schematic of the expected trajectories of CH as clonal sweeps (fixation) or patterns of clonal interference. **B,** VAF trajectories of CH variants with age from donor data (blue symbols), logistic fits of growth rates $r_{\log}$ (blue lines), and 95% CIs obtained from MLE. **C,** Fraction of increasing, decreasing, and saturating logistic fitnesses over 697 fitted variant trajectories. A successful fit is defined as all data points falling within the MLE-estimated 95% CI (Supplementary Methods S1). **D,** Clone sizes and trajectory composition with age. **E,** Growth rates $r_{\log}$ obtained from maximum likelihood estimation on 697 variant trajectories. **F,** The fraction of decreasing trajectories increases with age (Spearman correlation = 0.68; P=0.003). The mean growth rate decreases with age (Spearman correlation = −0.36; P=0.16). **G,** Realization of a stochastic simulation of CH from Equation A. **H,** Growth rates $r_{\log}$ measured from simulations of CH with parameters η=0.11, σ=0.032, and μ=5. **I,** The fraction of decreasing trajectories also increases with age in stochastic simulations (Spearman correlation = 0.75; P=0.0004), and the mean growth rate decreases with age (Spearman correlation = −0.53; P=0.028).

Here, we show that these phenomena all naturally arise in an evolutionary model of clonal competition, in which clones of differing fitness routinely occur and compete among each other. The result is an emerging fitness landscape that changes over time in a way that is highly variable between individuals (Fig. 1A). The clonal competition model generates longitudinal predictions on the distribution of variant trajectories and variant frequency distributions that are testable from *in vivo* observations and allow for a quantitative characterization of the evolutionary process driving CH. To this end, we developed 2 independent Bayesian computation frameworks to infer the fitness distribution and arrival rate of CH variants from either variant trajectories or HSC genetic diversity at single-cell resolution. Both frameworks quantitatively recover CH dynamics and converge onto the same underlying somatic evolutionary inferences. From these estimates, we observe variants of elevated fitnesses, with estimated arrival time distributions in agreement with expectations of a multistep model of clonal evolution (29, 34), suggesting that precursors of hematologic malignancies are distinguishable from nonprogressing CH and are identifiable prior to malignant transformation, potentially enabling a window of opportunity for early intervention.

## Results

### Trajectories of CH Suggest a Dynamic Fitness Landscape in HSCs

We first explored whether the hematopoietic fitness landscape changes noticeably throughout human life. We therefore revisited measurements of variant clone trajectories, obtained by Fabre and colleagues (15), of 56 known CH driver mutations across 385 individuals. This allowed us to follow in total 697 trajectories with 13 years of median follow-up time (Fig. 1B). We evaluated the observable growth rates for these 697 variants by fitting a logistic growth function to each variant trajectory independently. The logistic function has a sigmoidal shape that reflects the transition between early exponential growth and homeostatic cell turnover in a population of constant size. In population genetics theory, this is the natural trajectory of a single variant in the background of a wild-type (WT) population (31). The shape of this trajectory is characterized by the growth rate $r_{\log}$ that either is positive for increasing, negative for decreasing, or zero for saturating trajectories.

We obtained best-fit growth rates $r_{\log}$ for each variant trajectory by implementing a maximum likelihood estimator that assumes the measured variant frequencies to be binomially sampled from unobserved true frequencies due to limited sequencing resolution (Fig. 1B; Supplementary Methods S1). Overall, 67% of all successfully fitted trajectories were increasing, 24% decreasing, and 8% saturating (Fig. 1C and D). This resulted in a distribution of growth rates $r_{\log}$ (Fig. 1E) for individuals ranging from 62 to 98 years of age. Similar frequencies were recently reported by van Zeventer and colleagues (33) in a set of 1,642 mutations across 1,573 individuals. After stratifying trajectories by age, the relative proportion of decreasing trajectories increased from 18% at 63 years to 41% at 88 years (Spearman correlation = 0.68; *P* = 0.003), and the mean growth rate decreased with age (Spearman correlation = −0.36; *P* = 0.16; Fig. 1F). As variants in a population of approximately $10^{5}$ HSCs are unlikely to have reached detectable frequencies of 0.01 or higher solely by random drift (7, 8), those with detectable decreasing trajectories must have been under positive selection in the past to have reached high frequencies and subsequently lose that advantage (Fig. 1A). This suggests an emerging dynamic HSC fitness landscape in which clone growth rates can change with age.

### Clonal Competition Predicts Varying CH Trajectories within and across Individuals

We next explored whether clonal evolution of the HSC population could explain these age-related changes (Fig. 1D–F). The logistic model—in which a single positively selected clone expands in the background of a WT population with constant fitness—cannot by itself predict the observed changes in growth rates of variant trajectories with age (Fig. 1A; ref. 32). We therefore extended the single-clone model to allow for the emergence of arbitrarily many clones in a stem cell population of fixed size. This is motivated by the notion that the overall number of HSCs is regulated, for which there is now evidence from indirect measurements in humans (2, 16), BM transplant studies (25), and direct measurements in mouse hematopoiesis (35). We modeled the occurrence of driver mutations (variants that confer a fitness advantage relative to the WT HSC population) by having new clones arise with constant mutation rate μ at random times according to a Poisson process. To model the possible breadth of many different driver mutations, each clone is at birth assigned a fitness parameter $s_{i}$ randomly drawn from a gamma distribution, a flexible choice that contains many families of probability distributions as limiting cases. This $s_{i}$—which we will hereafter refer to as the clone’s innate fitness—is defined as the difference between the clone’s proliferation rate and that of a WT HSC. We find (see Supplementary Methods S2) that the fully stochastic birth–death process of an arbitrary number of clones obeys a set of stochastic differential equations given by

$$dx_{i}\left(t\right)=\left(s_{i}-\underset{\text{all}\;\text{clones}}{\sum }s_{j}x_{j}\left(t\right)\right)dt+B\left(t\right)\;dW_{t}$$ (A)

The above equation describes the change in the frequencies $x_{i}$ of clones i with time t. The first term on the right-hand side corresponds to a system of competitive Lotka–Volterra equations (36), which has a long-standing tradition in ecology and evolution (37), although here the number of equations changes as clones emerge or go extinct. The second term adds stochastic fluctuations with Gaussian noise. In this model, clonal competition emerges naturally, as HSCs of differing innate fitness continuously emerge and compete for space within the capacity-limited stem cell population (Fig. 1G; Supplementary Fig. S1A–S1D). We confirmed the validity of Equation A with agent-based simulations of the underlying birth–death process using the Gillespie algorithm (Supplementary Methods S4; Supplementary Fig. S1E; ref. 38).

This model highlights the importance of distinguishing between a variant’s innate fitness $s_{i}$ and its observable growth rate $r_{\log}$ within the HSC population. Although a clone with positive innate fitness will always outcompete WT HSCs and therefore grows in the absence of other clones, a polyclonal stem cell population containing fitter clones can force its decline. This occurs because the rate at which a clone grows—the right-hand side of Equation A—is proportional to the difference between its own innate fitness and the average fitness in the HSC population. This average population fitness increases as more clones arise and expand and can eventually surpass that of individual clones with modest positive innate fitness, flipping their growth rate from positive to negative. This becomes more likely with age as more clones inhabit the HSC population.

We first tested if such a model of clonal competition can qualitatively reproduce the observed *in vivo* properties of variant trajectories. Using Equation A, we simulated clone trajectories in virtual individuals that matched the age and frequency of clones in the Fabre data examined previously. We then applied the same maximum likelihood estimation (MLE; Supplementary Methods S1) to obtain the age-stratified distribution of growth rates $r_{\log}$ from simulated trajectories (Fig. 1H). In accordance with the data, simulations show the emergence of decreasing variant trajectories with increasing abundance with age (Spearman correlation = 0.75; P=0.0004), although the mean growth rate across all variants decreases slowly with age (Spearman correlation = −0.53; P=0.028; Fig. 1I). The model furthermore predicts that the growth rate $r_{\log}$ of a variant varies strongly with its time of occurrence, the individual’s age at measurement, and the unique landscape of co-occurring mutations (Supplementary Fig. S1F). This provides a natural explanation for the observed variation in growth rates of identical CH driver mutations across individuals (15, 30, 33). Together, these findings suggest that clonal competition could underlie the dynamic and stochastic nature of observed clone trajectories.

### Characteristic Transitions of Site Frequency Spectra with Age Reflect HSC Population Dynamics

**Figure 2.**
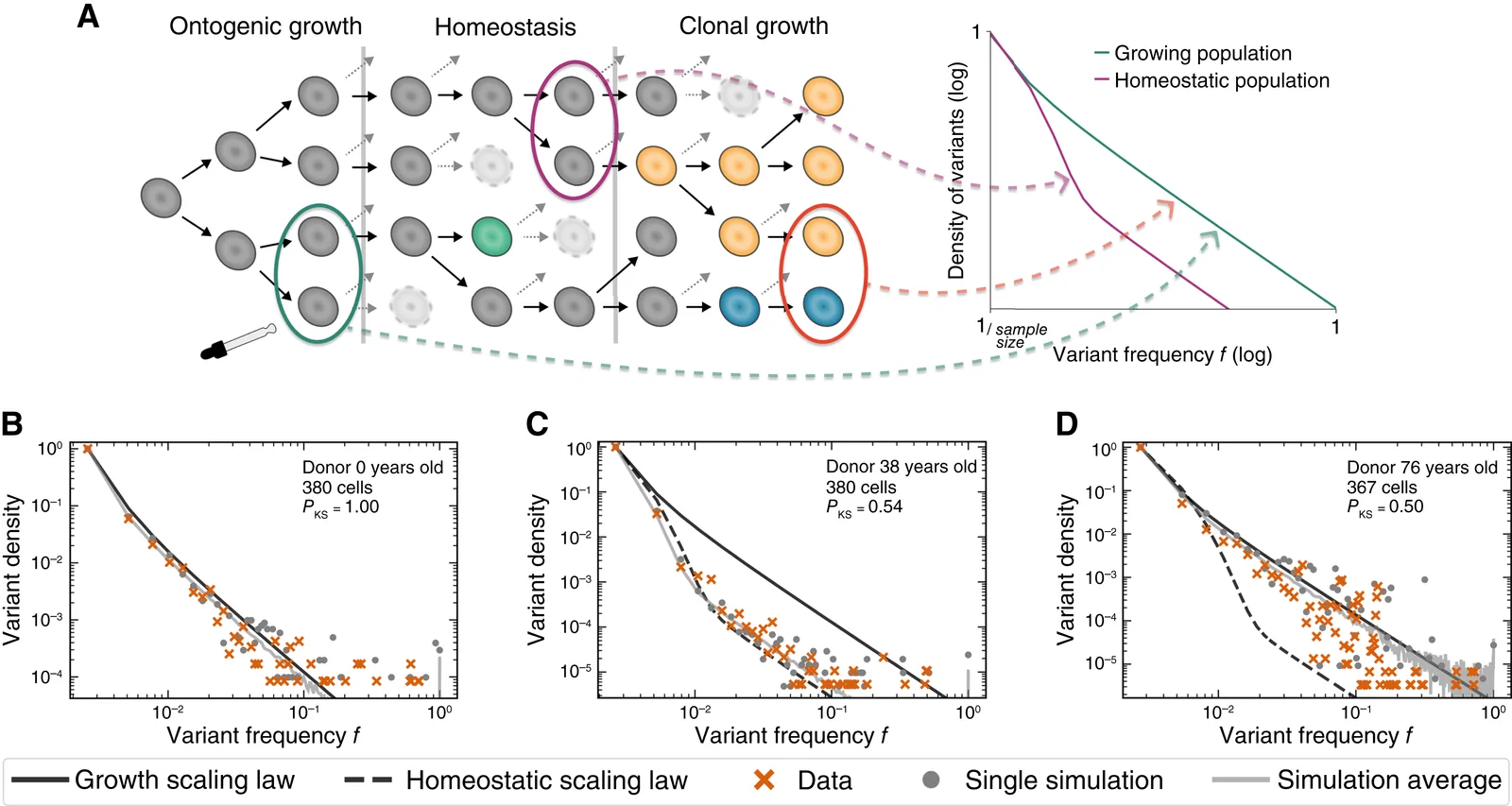
Transitions of HSC SFSs with age in a polyclonal competition model. **A,** Schematic of the expected scaling shift of the SFS under ontogenic growth, homeostasis, and clonal expansion of HSCs. Theory predicts 3 distinct phases of the SFS with age in the presence of clonal competition. **B,** The SFS of a newborn reconstructed from 390 whole genome–sequenced single cells (orange crosses), a single realization of the agent-based simulation (gray dots), averages more than 500 simulations (gray line) and the expected scaling of an exponentially growing population (black line). **C,** SFS in a 38-year-old donor constructed from 380 single HSCs. The SFS has shifted toward the expected scaling of a homeostatic population of constant size (black dashed line). **D,** SFS in a 76-year-old donor constructed from 367 single HSCs. The SFS reverts to the scaling of a growing population at frequencies below <0.1 (black line), and subclonal peaks become evident.

Recent studies have suggested changing patterns of genetic heterogeneity of the HSC population throughout the human lifespan (2, 16). We next investigated whether these changes are consistent with a polyclonal competition model of CH evolution. To this end, we quantified genetic heterogeneity using the site frequency spectrum (SFS)—the distribution of prevalences of genetic variants within a population. The SFS is typically dominated by neutral mutations which arise far more frequently than positively selected variants. However, neutral mutations hitchhike on expanding fit clones (39), suggesting that a polyclonal HSC population may change the SFS with age (Fig. 2A). To derive predictions for the expected SFS in the presence of clonal competition, we developed agent-based simulations that include the accumulation of neutral variants in addition to drivers in a population of $10^{5}$ HSCs (Supplementary Methods S4; Supplementary Fig. S2A–S2I). From these simulations, we observe 2 frequency domains of the SFS (Fig. 2B–D; Supplementary Fig. S3A–S3I). Variants at high frequencies ($≳10^{-1}$) are sparse and highly stochastic across realizations (Supplementary Fig. S4). At young ages, they are predominantly developmental in origin, whereas in the elderly they are a combination of developmental and positively selected variants. In contrast, at lower frequencies ($≲10^{-1}$), variant distributions follow power laws that emerge from the underlying HSC dynamics (Fig. 2A). At birth, the number of mutations at frequency f follows a $1/f^{2}$ power law. This is characteristic for expanding populations and commonly observed in tumor bulk sequencing (40–42). Here, it occurs as a consequence of developmental growth. By early adulthood, the SFS approaches a 1/f power law, which is associated with a neutral homeostatic population of fixed size (10, 42, 43). In the elderly, our simulations suggest that the SFS shifts again, with variants at low frequency reverting to a $1/f^{2}$ scaling because of the expansion of positively selected clones. Thus, our stochastic simulations predict 2 transitions between 3 characteristic stages of the SFS in HSCs. The first reflects a deterministic change in the population-level dynamics from a growing into a constant homeostatic HSC population. The second is driven by subclonal expansions and would be absent in a neutrally evolving population.

We tested these predictions in SFSs reconstructed from single-cell whole-genome sequencing (WGS) data of HSCs published by Mitchell and colleagues (Fig. 2B–D; ref. 16). WGS of 200 to 500 single HSCs for each of the 9 healthy donors 0 to 81 years of age allowed us to compute SFSs with high resolution across the observed frequency spectrum. After adjusting for single-cell sampling in our simulations (Supplementary Fig. S5A–S5D), measured spectra of all ages agree with stochastic simulations (Kolmogorov–Smirnov test $P_{KS}>0.05$ for all SFSs; Fig. 2B–D; Supplementary Fig. S3). HSC-derived SFSs indeed exhibit the predicted transitions throughout life. Furthermore, although the SFSs in newborns and the elderly both follow the same scaling law at low frequencies, the elderly exhibit increased variance at higher frequencies. Single realizations of our stochastic simulations show similar behavior (Supplementary Fig. S3). This is due to neutral mutations hitchhiking on expanding clones (44), resulting in sporadic peaks at high frequencies in the SFS that are not visible in the average over simulations. The frequency and magnitude of these peaks are determined by the clones’ evolutionary histories, which are unique to every individual. Notably, this resembles signals of subclonal selection in tumors (44). In summary, a clonal competition model precisely recovers the nontrivial dynamics of the SFS from newborns to octogenarians, capturing the changes in the genetic heterogeneity observed in CH at single-cell resolution.

### CH Evolutionary Parameters Are Identifiable with Bayesian Computation

**Figure 3.**
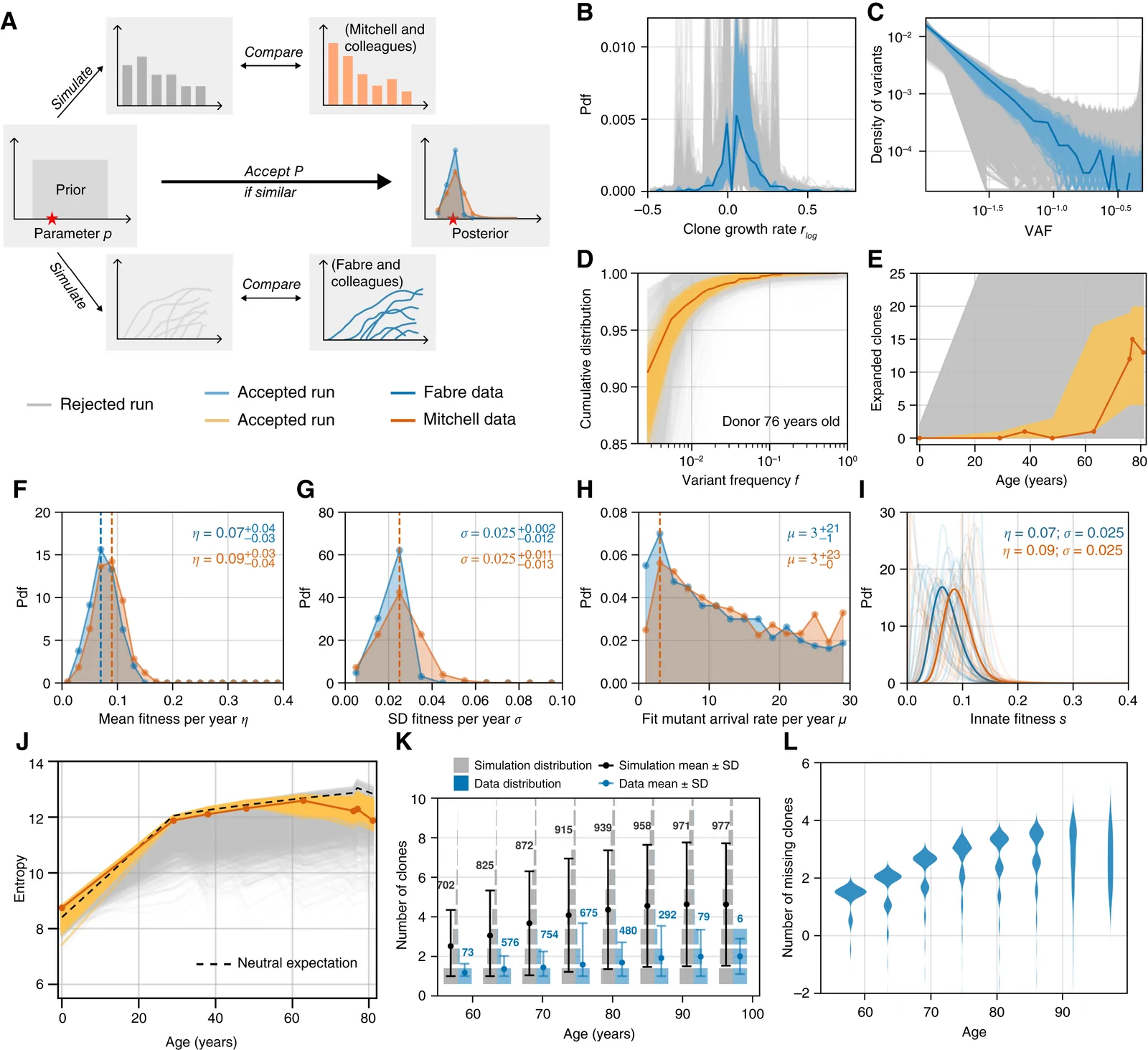
Inferences of the innate fitness distribution and arrival rate of advantageous clones in CH. **A,** Schematic of the ABC framework. **B,** Growth rate distribution $r_{\log}$*in vivo* (dark blue lines), in ABC-accepted runs (light blue lines), and in ABC-rejected runs (gray lines). **C,** Clone size distribution *in vivo* (dark blue lines), in ABC-accepted runs (light blue lines), and in ABC-rejected runs (gray lines) runs. **D,** Cumulative SFS of the 76-year-old donor (dark orange lines), in ABC-accepted runs (light orange lines), and in ABC-rejected runs (gray area). **E,** Number of visible clones at 1% resolution *in vivo* (orange dots), in ABC-accepted runs (orange shaded area), and in ABC-rejected runs (gray shaded area). **F–H,** Posterior ABC parameter estimates based on trajectories (blue) and SFS (orange) for the mean η and the SD σ of the innate fitness distribution of advantageous variants and the HSC population arrival rate μ per year of advantageous variants. Uniform prior distributions: η∈[0.01,0.4], σ∈[0.001,0.1], and μ∈[0.1,30]. **I,** Best estimates of the innate fitness distribution of advantageous variants based on trajectories (blue) and SFS (orange). Thin lines represent individual accepted runs. Thick lines correspond to the estimated mean and SD. **J,** Entropy computed from the *in vivo* SFS (dark orange lines), simulated SFS of the ABC-accepted runs (light orange lines), and simulated SFS of the ABC-rejected runs (gray area). The neutral expectation (dashed black line) is the average of more than 100 SFSs simulated with neutral dynamics. **K,** Number of observable (>1% VAF) clones predicted by simulations (gray) and observed in targeted sequencing data (blue) from 58 to 97 years of age. For each age bin, the distribution across simulations/donors (horizontal bars, sample size shown as number), the mean (circles), and SD (brackets). **L,** Expanded clones missed by target sequencing estimated as the difference between the number of clones predicted by our model and the number of clones detected from target sequencing samples. pdf, probability density function.

We have shown that clonal competition qualitatively recovers the dynamics of clonal trajectories and SFSs in human hematopoiesis. However, the parameters underlying CH evolution in humans so far remain unknown. We therefore next explored whether these parameters are identifiable from variant trajectories or SFSs (Fig. 3A). The clonal competition model requires 3 parameters that determine the HSC fitness landscape: the arrival rate μ of advantageous variants and the mean η and standard deviation (SD) σ of their innate fitness distribution. We additionally require the size of the adult HSC population N and the WT HSC interdivision time τ in order to fully implement the stochastic simulations. These 2 parameters describe healthy hematopoiesis, with typical estimates on the order of $10^{5}$ HSCs and each dividing around once per year (2, 16, 45).

We first tested the identifiability of these parameters from synthetic data. To this end, we first generated simulations of variant allele frequency (VAF)–measured variant trajectories (obtained from Equation A) and single cell–derived SFSs across a broad range of ages [from the agent-based modeling (ABM) simulations] using a predefined parameter set $P^{*}=\left\{\mu^{*},\eta^{*},\sigma^{*},N^{*},\tau^{*}\right\}$. Then, we implemented approximate Bayesian computation (ABC) inference schemes (46) to estimate $P^{*}$ from these synthetic data. In brief, this consisted of first randomly drawing 65,000 parameter combinations $P_{i}=\left\{\mu_{i},\eta_{i},\sigma_{i},N_{i},\tau_{i}\right\}$ from uniform prior distributions. Then, for each $P_{i}$, we generated *in silico* predictions from our modeling and quantitatively compared them with the synthetic data to obtain metric distances. The metric of comparison depended on the type of data. For variant trajectories, we used the distribution of clone sizes and the distribution of fitted growth rates $r_{\log}$ (Fig. 3B and C). For the SFSs, we compared the Wasserstein distances (Fig. 3D; Supplementary Fig. S6A–S6H) and the number of detectable clones above 1% frequency (Fig. 3E; Supplementary Fig. S6I). See also Supplementary Methods S5 for details. We then retained the 1% quantile of ranked distances to derive posterior distributions containing those parameter combinations $P_{i}$ with predictions closest to the synthetic data (Fig. 3A). Both variant trajectories and the SFSs enabled sharp inferences of the mean and variance of the imposed variant fitness distribution (Supplementary Figs. S7A, S7B, and S8A–S8F). We also found agreement between estimated and imposed variant arrival rates, although with larger credible intervals (CI). Consistent with previous observations (2, 7, 16), the stem cell population size N and interdivision time τ were not separately inferrable solely from sequencing information.

### Estimates of Human CH Evolutionary Parameters Converge Independently on Variant Trajectories and SFSs

We next inferred the evolutionary parameters of human CH by applying the same ABC inference schemes to longitudinal variant trajectories and SFSs. The former comprised ∼700 trajectories from ∼400 donors [Fabre and colleagues (15)]. The latter consisted of single cell–expanded HSC colonies from 9 individuals 0 to 81 years of age [Mitchell and colleagues (16)]. Uniform prior distributions for the ABC inference were chosen such that they covered plausible ranges of the parameters (see Supplementary Methods S3). The posterior distributions for the competition parameters (η, σ, and μ) converge across both datasets and deviate significantly from the uniform priors in both ABC inference schemes (Fig. 3F–H; Supplementary Figs. S9A–S9C and S10A–S10F). We report mode and 90% credibility intervals for best parameter values. From the inference on variant trajectories (Fig. 3B and C), we obtain for the mean and the SD of the innate fitness distribution $\eta =0.07_{-0.03}^{+0.04}$ per year and $\sigma =0.025_{-0.012}^{+0.002}$ (Fig. 3F and G), whereas the inference on the SFS (Fig. 3D and E; Supplementary Fig. S11A–S11H) estimated these at $\eta =0.09_{-0.04}^{+0.03}$ per year and $\sigma =0.025_{-0.013}^{+0.011}$ (Fig. 3F and G), respectively. Thus, we find strong agreement between both datasets (Supplementary Figs. S12A–S12I and S13A–S13I). For the arrival rate of advantageous variants, we find from trajectory and SFS data, respectively, $\mu =3_{-1}^{+21}$ and $\mu =3_{-0}^{+23}$ per year (Fig. 3H). Although these posteriors are again in agreement across both datasets, the convergence is weaker, as was also observed in *in silico* inferences. We therefore ran additional ABC inferences on the data with a wider prior distribution for the arrival rate (μ∈[1, 512]) and obtained similar estimates for μ, with posterior distributions that decreased rapidly for increasing μ (Supplementary Figs. S14 and S15). In contrast, the posterior parameter distributions for the HSC population size N and the HSC WT interdivision time τ demonstrate no convergence (Supplementary Figs. S10C and S16). This is in accordance with our validation of the ABC framework on synthetic data (Supplementary Methods S3; Supplementary Fig. S7B) and implies that the competition model is compatible with a wide range of these parameters. We confirmed this observation with another set of ABC inferences with N and τ fixed at best current estimates of $10^{5}$ HSCs dividing once per year, which resulted in almost identical estimates for the remaining parameters (Supplementary Figs. S17 and S18).

The inferred innate fitness distribution peaks at a positive non-zero value (Fig. 3I), implying that clones with very low positive innate fitness (s≈0) are rare. To ensure that this result is not due to limited data resolution—as clones with low innate fitness are less likely to expand to observable frequencies—we performed ABC inferences on simulated data with an imposed exponential innate fitness distribution measured at similarly limited sequencing resolution. The ABC successfully identified the correct innate fitness distribution despite the lowest fitness variants not being directly observed (Supplementary Fig. S7A). This demonstrates that even variants with low positive innate fitness convey a measurable effect on the system dynamics through compounding interactions with other clones. It is worth noting that—due to the nature of the data—the inferred innate fitness distribution (Fig. 3I) describes the prevalence of advantageous variants that have not yet transformed into hematologic malignancies and thus potentially does not describe the fittest variants occurring at high s. This agrees with recent estimates of acute myeloid leukemia (AML) subclonal driver mutations with estimated fitnesses between 0.4 and 0.8 (44) and estimates of the most common mosaic chromosomal aberrations in blood that aggregate at the upper bound of 0.1 to 0.2 per year of the fitness distribution (47).

The parameter inference finds an arrival rate of fit clones on the order of 2 to 25 per year, suggesting that by 50 years, an individual may have harbored up to a 1,000 clones under positive selection, most of which reside in a single HSC or have gone extinct. Although this may seem high, the fraction of fitness-conferring mutations is still low overall (Fig. 3J). In a population of $10^{5}$ HSCs with a total rate of 20 mutations per year per stem cell (5), this estimate results in 1 to 10 in $10^{6}$ mutations conferring a selective advantage. Furthermore, the estimated innate fitness distribution has an average of 7% to 9% per year. Thus, the combined effects of low fitness and competition prevent most clones from escaping extinction and reaching detectable sizes. Taken together, these inferences suggest that positively selected variants are ubiquitous in human HSC populations, begin to accumulate early, and typically become detectable in the fifth decade of life at current sequencing resolution of 1%.

### The Expected Number of Undetected CH Clones in Current Targeted Sequencing Panels Increases with Age

Given the ubiquity of polyclonal CH in the elderly, we next estimated how many expanding clones might be missed with current targeted sequencing panels of known CH driver genes. As our parameterized model predicts the number of expanded clones with age, we compared this quantity with observations in a large cohort of individuals. To this end, we integrated the previously analyzed trajectory data (15) with 2 additional datasets (see Supplementary Methods S6; Supplementary Fig. S19A–S19G; refs. 30, 33). In these data, the average number of observed clones (>1.5% VAF) per individual increased from 1 to 2 over the course of 30 years (55–85 years of age; Fig. 3K). Interestingly, the number of clones levels off at 1.9 in individuals 85 years and older. This trend is also observed in simulations of our model. Across 1,000 simulations with parameters sampled from the previously obtained posteriors, the model predicts a similar linear increase to occur over the same age range, from an average 2.5 expanded clones at 50 years to 4.6 at 85 years, and a leveling off after (Fig. 3K; Supplementary Fig. S20). The variability of detectable clones between individuals is high and increases with age (SD at 50: 1.8; SD at 85: 3.9). Taken together, this suggests that current targeted sequencing panels miss on average 53% at 55 years (up to 76% at 90% credible interval) and 58% at 85 years (up to 79% at 90% credible interval) of expanding clones in the HSC population (Fig. 3L). This is in line with a previous estimate derived from variant frequency distributions in the human population (8), which suggested that on average known drivers of CH account for only 30% of all suspected clonal expansions. Here, we extend this estimate by deriving age-dependent ranges for the interindividual variation. Overall, these estimates are corroborated by the ongoing discovery of novel CH drivers (48, 49), the high prevalence of “driverless” CH (16), and at times missing drivers during malignant transformation (bioRxiv 2024.07.05.602251). Some of these undetected clones may derive from nontargeted genic regions, noncoding regions, structural variants, or epigenetic modifications.

### Innate Fitness and Arrival Time Inferences Uncover a Multistep Model of CH Evolution

**Figure 4.**
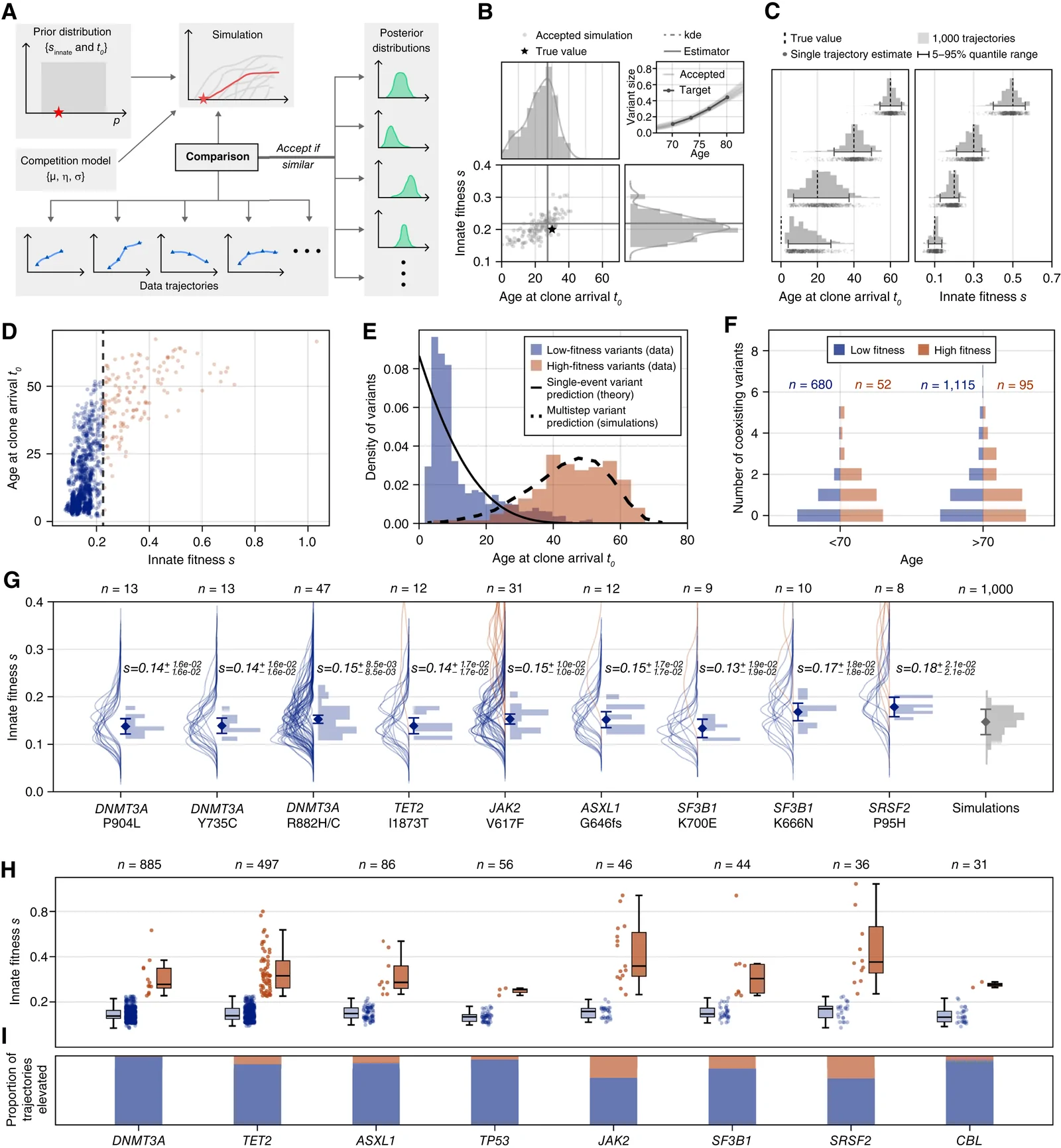
Innate fitness estimates and timing of individual variant trajectories. **A,** Schematic of the ABC framework to infer arrival time $t_{0}$ and innate fitness s of individual variants. **B,** Example outcome of the ABC for a single simulated variant trajectory. **C,** Distribution of ABC estimates on simulated data with fixed values (dashed vertical lines, 1,000 trajectories per value). **D,** Estimates of arrival time $t_{0}$ and fitness s of 1,942 variant trajectories from 3 datasets (Fabre, Robertson, and van Zeventer). Trajectories are segregated in either low (s<0.226, blue circles) or high (s>0.226, red circles) fitness upon unsupervised clustering. Decision boundary (dashed line) estimated at s=0.226 by Gaussian clustering with 2 components. **E,** Distribution of arrival times of both low (blue bars) and high (red) fitness variants compared with predictions from theory (solid line) and simulations (dashed line) for single step and multistep clonal evolution. Distributions are normalized independently for better visualization. **F,** Distribution of the number of observed variants that coexist with the target variant, for low (blue left facing bars) and high (red right facing bars) fitness, in individuals younger than (left) and older than (right) 70 years of age. **G,** ABC-derived posterior distributions (left facing solid lines) for innate fitness s of most common identical variants across multiple individuals (number shown above). Right facing histograms show distributions of innate fitness estimates (maxima of posteriors). Rangebars denote twice the standard error above and below the mean (diamonds). Estimates of *in silico* variants (far right, gray) from 1,000 simulated trajectories with random arrival times $t_{0}\in \left[0,60\right]$ and innate fitness s=0.15. **H,** Estimated innate fitnesses stratified by most commonly mutated genes. **I,** Per gene proportions of low (blue) and high (red) fitness drivers. kde, kernel density estimation.

From the above ABC frameworks, we inferred the evolutionary parameters of CH on a population level. However, the question remains whether the innate fitnesses of individual clones are identifiable in this evolving HSC fitness landscape. Complications arise because clonal trajectories depend on both innate fitness and competitive interactions with other clones, many of which may be undetected. To resolve this, we note that the parameterized competition model predicts the distribution of all possible trajectories of a clone arising at a particular age and fitness. This enables inferences of the innate fitness and arrival time most likely to result in a given trajectory at a given age. We first tested this approach against simulated data. We embedded 1,000 clones with predetermined innate fitness s and arrival time $t_{0}$ in independent realizations of the stochastic differential equation (SDE) model (Fig. 4A). This resulted in a set of clone trajectories with known initial conditions. We then performed an ABC comparing this *in silico* dataset with 150,000 simulated trajectories with uniform randomly drawn innate fitnesses and arrival times (see Supplementary Methods S7 for details). Taking the maxima of the resulting marginalized posteriors (Fig. 4B), we found that the distribution of estimates centered around imposed values, with increasing variance for higher fitness and earlier arrival times. Thus, both innate fitness and arrival time of clones were identifiable from their trajectories (Fig. 4C).

Next, we applied this framework to the 1,942 variant trajectories in the aggregated dataset (Supplementary Methods S6; refs. 15, 30, 33). For all trajectories, the marginalized posterior distributions converged in both parameters. The majority had innate fitness estimates between 10% and 20% per year, placing them above the mean of the innate fitness distribution, in line with the expectation that only sufficiently fit clones reach detection (Fig. 1G). Only a small subset of variant trajectories presented with higher fitnesses. Notably, these tended to arise much later in life, whereas the lower fitness trajectories scattered across all ages (Fig. 4D and E). This observation is predicted by a multistep model of clonal evolution, in which successive driver mutations within a single clone progressively increase its fitness. To test whether we can distinguish between clones expanding through a sole driver and those which have undergone multiple driver events, we performed a 2-component Gaussian mixture model clustering on all estimated innate fitnesses. This resulted in a partition of the trajectories with innate fitnesses above (147 of 1,942) and below (1,795 of 1,942) a threshold $s_{T}=0.226$ (Supplementary Methods S8). Interestingly, the estimated arrival times of the lower fitness ($s<s_{T}$) variants roughly follow the expectation of single events occurring with a constant rate throughout life, whereas those of high fitness match simulated observations of a multistep process (2-sample Kolmogorov–Smirnov test: P=0.27; Fig. 4E; Supplementary Fig. S21; Supplementary Methods S8). Such a multistep model of clonal evolution is further supported by clonal co-occurrence patterns. In donors younger than 70 years of age, the high fitness trajectories had a higher likelihood of being detected alongside at least one other driver (62% of highest fitness trajectories vs. 42% in other drivers; P=0.0059; Mann–Whitney U test) and had a higher average number of co-occurring drivers (1.10 vs. 0.62; p=0.0010; Mann–Whitney U test; Fig. 4F). These statistical differences vanish at older ages (>70), likely as individuals harbor increasingly more detectable clones masking correlations of co-occurrence patterns. Taken together, this suggests that the lower fitness trajectories ($<s_{T}$) are probably derived from single events, and their estimated fitnesses may be identified as the innate fitness of their driver. In contrast, for higher fitness variants ($>s_{T}$), multiple steps have likely elevated their fitness above each driver’s solitary innate value.

### The Propensity for Elevated Fitness Differs across Genes

Surprisingly, none of the combined 73 trajectories of the most common *DNMT3A* variants were estimated to have elevated ($>s_{T}$) fitness (Fig. 4G). In contrast, in *JAK2*.pV617F, *SRSF2*.p95H, and *TET2*.1873T, we observe trajectories with elevated fitness despite small sample sizes. The same trend remains if we compare estimated innate fitness across genes. Only 10 of 885 *DNMT3A* variants presented elevated fitness, as opposed to 51 of 497 *TET2* and 11 of 36 *SRSF2* variants. Overall, the propensity of elevated fitness in *DNMT3A* is significantly lower than the next most frequent CH driver genes *TET2* ($P=5.4\times 10^{-17}$; Fisher exact test), *ASXL1* ($P=5.4\times 10^{-5}$), *JAK2* ($P=5.2\times 10^{-14}$), *SF3B1* ($P=2.2\times 10^{-6}$), and *SRSF2* ($P=7\times 10^{-12}$), with the notable exceptions of *TP53* (P=0.11), and *CBL* (P=0.16; Fig. 4H and I). These different propensities suggest epistatic interactions among common CH variants, the details of which remain largely unknown. Among the 9 most commonly mutated genes, mean elevated fitness ranged from 0.26 to 049, with noticeable variation within and between genes (Fig. 4H). The highest observed fitness was 1.03 in 1 mutated *SRSF2* clone, followed by 0.72 also in *SRSF2*. These ranges are consistent with recent retrospective fitness inferences of preleukemic clones with 2 to 4 accumulated driver mutations that had transformed into AML (bioRxiv 2024.07.05.602251). This suggests that variant innate fitness and arrival time estimates could conceivably allow for identification of clones at high risk of progression, even at current clinical resolution.

### Clonal Competition Can Account for Most of the Interindividual Variant Growth Rate Variation

Identical variants present with largely varying growth rates across individuals and age (15, 33). Although these growth rates are often equated to fitness, we have shown that a competitive fitness landscape predicts a high variability of observable growth rates, even if underlying innate fitnesses are similar. This elicits the question of how much of this observed heterogeneity is explainable by competition. To address this, we calculated the fraction of variance unexplained—here defined as the ratio of predicted to empirically observed variance (50)—by comparing the variation in innate fitness estimates on simulated trajectories with that observed in the data (see Supplementary Methods S7 for details). We assessed the variation in the data by examining identically mutated sites across multiple donors. The variants most commonly occurring (*n*≥ 11) among the 1,942 trajectories comprised 3 mutations in *DNMT3A* (*n* = 47, *n* = 13, and *n* = 13), 2 in *SF3B1* (*n* = 12 and *n* = 11), and single mutations in *JAK2* (*n* = 46), *SRSF2* (*n* = 16), *TET2* (*n* = 14), and *ASXL1* (*n* = 13). For each distinct variant, we obtained a distribution of fitness estimates from the donor trajectories; e.g., for the *DNMT3A*.R882H/C variant, this resulted in 47 independent innate fitness estimates (Fig. 4G). Taking the mean across donors—excluding multistep trajectories (s>0.226)—as estimate for the variants’ innate fitness, these ranged from 0.14 to 0.18, with standard errors between 0.004 and 0.009 (Fig. 4G; Supplementary Table S1). To obtain an empirically observed variance, we focused on the 2 most abundant variants in the data—*DNTM3A*.R882H/C and *JAK2*.V617F (46 and 31 trajectories). Both had a mean innate fitness at s=0.15, with variances of $8.7\times 10^{-4}$ and $6.3\times 10^{-4}$, respectively. We took the weighted average of these as the total variance estimate and calculated a 95% CI (Supplementary Methods S7), resulting in $\sigma_{obs}^{2}=7.2_{-1.9}^{+3.2}\times 10^{-4}$. We then performed the same innate fitness inference on 1,000 simulated trajectories with random arrival times and fixed innate fitness s = 0.15, resulting in a distribution with variance $\sigma_{sim}^{2}=7.1\times 10^{-4}$ (Fig. 4G). At the lower bound of $\sigma_{obs}^{2}$, the observed variance of the innate fitness in the data is slightly smaller than that in the simulations, suggesting that in the best-case scenario, all of the interindividual variation in growth rates of identical variants could be explained by the model. However, taking the upper bound of the error as a more conservative estimate of $\sigma_{obs}^{2}$, 65% of the observed growth rate variation can be explained by our model of polyclonal competition. Indeed, the variation predicted by the model cannot all be attributed to competition, as even in a model without it [e.g., the model by Watson and colleagues (7)], there can be variation in the estimated growth rates because of the stochasticity of clonal expansions. To control for possible biases introduced by including trajectories also used for model fitting (Supplementary Methods S3), we repeated the variance analysis excluding trajectories obtained by Fabre and colleagues (15). From the remaining 70 trajectories (43 *DNTM3A*.R882H/C and 27 *JAK2*.V617F), we obtained $\sigma_{obs}^{2}=7.2_{-1.9}^{+3.2}\times 10^{-4}$, and thus at least 69% of the growth rate variation was explained by our model, consistent with the previous estimate. It will be important for future work to further narrow these estimates when more trajectories of identically mutated sites become available.

## Discussion

The clonal competition model arises from 2 premises: (i) The accumulation of mutations with age is unavoidable and highly conserved across individuals; (ii) in homeostasis, the HSC population is regulated to maintain its size. The former is now supported by overwhelming evidence (3, 5, 16, 27, 29). The latter is perhaps most strongly illustrated by the BM’s ability to swiftly repopulate its HSC pool following transplant (25, 51). This size regulation has been directly observed in mice through grafting (36) and is indirectly supported in humans by observations of consistent telomere attrition (52, 53) and phylogenetic tree reconstruction (2, 16). Although it is possible that certain nonmalignant or premalignant variants allow partial escape from such regulation, a model of unconstrained exponential growth for all advantageous variants would result in a 5- to 100-fold increase in the stem cell population late in life (see Supplementary Methods S2; Supplementary Fig. S22A and S22B), for which there is currently little evidence in human hematopoiesis. Conversely, certain conditions suggest a reduced stem cell population size; e.g., patients with aplastic anemia present with accelerated telomere shortening (54). Interestingly, in the case of chronic myeloid leukemia, the BCR-ABL–positive HSC population seems to increase over time (55), whereas in chronic lymphocytic leukemia, the estimated HSC population is about the size of the normal HSC population (56). Collectively, these conditions are well-defined severely dysregulated states, with characteristically altered malignant phenotypes compared with most CH variants in the aging population.

The model of polyclonal evolution presented here offers a natural explanation for multiple paradoxical unexplained observations of CH, such as declining variant trajectories in the elderly, an overall fitness decline with age, and excessive interindividual growth rate variation. Longitudinal data of patients progressed to AML reveal similar clonal dynamics in malignant precursors in aged individuals (bioRxiv 2024.07.05.602251). Nevertheless, multiple additional factors possibly contribute to the development of CH. For example, declining trajectories might result from immunosurveillance acting on expanded clones, if these were to express tumor-specific antigens identifiable by the immune system (57). However, a recent study found no association between MHC binding affinity and CH prevalence for common CH hotspot mutations (bioRxiv 2024.09.27.615394), suggesting a limited ability for immunosurveillance to detect premalignant CH clones. This is further supported by the typically slow decline over multiple years of variant trajectories in the elderly (15), an unlikely signature of an acute immune response. The overall fitness decline at old ages could be due to a growing burden of deleterious variants—given the linear accumulation of mutations with age (5), although the weak signal of negative selection in hematopoiesis (58, 59) does not strongly support this. Still, other age-related changes might contribute to the observed fitness reduction. Stem cell aging could cause reduced proliferation ability (60, 61), although the *in vivo* effect of this has not yet been shown. Telomere shortening might also play a role, although typically telomeres of HSCs are not critically short in the elderly (25, 52, 53). External microenvironmental processes such as aging BM niches and increased inflammation may also contribute. Low-grade inflammation is a key associate to aging (62) and can be driven by various CH variants (14). Notably, although inflammation can increase mutagenesis (63), some expanding CH variants may derive an increased fitness from the adaptation to inflammatory signaling (63, 64). Environmental risk factors could potentially contribute to this, although it is important to distinguish between mutagenic processes that affect driver acquisition (65) and those that—through epistatic effects—affect fitness (29, 66). The lack of correlation between clonal expansion rates and typical risk factors like smoking, alcohol intake, and body size (33) suggests that they may relate more to CH initiation than clonal expansion. Finally, although germline variants can affect CH and increase the risk of cancer progression, these variants are rare and cannot account for the ubiquity of growth rate variation between individuals and age (21, 67, 68).

Because our model is agnostic to functional differences of CH driver mutations, it cannot directly inform on mechanisms underpinning the selective advantage of expanding clones. In general, variants in common CH genes such as *DNMT3A* and *TET2* tend to disrupt DNA methylation, skewing the differentiation landscape of hematopoiesis toward increased HSC self-renewal (69–71). In contrast, it has been suggested that clones with common splicing variants in *SF3B1* or *SRSF2* are prone to cryptic splicing among multiple genes involved in terminal HSC differentiation (72). Moreover, splicing-factor variants seem to slow telomere shortening in aged individuals, possibly rescuing HSCs from critical telomere shortening (73). The functional interaction of co-occurring CH variants, however, remains largely unknown.

The clear distinction in estimated arrival times between low and high fitness variants highlights the role of accumulating driver events along the path of neoplastic transformation and the importance of better understanding this process. For example, the considerable underrepresentation of *DNMT3A* variants among elevated fitness trajectories is a surprising observation that warrants further investigation. *DNMT3A*.R882 has high estimated innate fitness and plays a prominent role in transformation to AML. Yet, despite its dominant prevalence in CH, it seems to co-occur less frequently with many other AML driver mutations (74). This might speculatively point to a rugged fitness landscape of *DNMT3A* and co-occurring variants—with few viable variant combinations—implying a slow transition rate and higher prevalence of single-step *DNMT3A* variants in CH. Conversely, the absence of elevated fitness *DNMT3A* trajectories may also point to a survivorship bias, in which rare jackpot events lead to fast progression. It also remains an open question if all *DNMT3A*.R882 variants are equally likely to transition, or if there are differences in the patterns of hypomethylation that prime some over others. Answers may come from evolutionary models of clonal competition combined with single-cell analysis (71). We expect that with higher resolution additional effects such as age-related HSC deregulation, immune interactions, inflammation, epistasis, and frequency dependence may eventually become identifiable as well. The identifiability of multihit clones also suggests potential for risk stratification of CH trajectories across individuals and age, possibly even in a driver mutation–agnostic manner. In principle, sequencing any sufficiently large indicator region of the human genome would suffice to identify high-risk clones, as any mutation within that region also carried by an unseen clone would hitchhike with the same growth rate. Importantly, both fast-growing and fast-shrinking clones can be indicators of an undetected premalignancy. Once the presence of such a high-risk clone is suspected, a more detailed genomic analysis could potentially identify the underlying causal genomic alterations and inform early intervention strategies.

Lastly, it has been speculated that clonal competition may act as a barrier to some malignant expansions, as widespread competition among a large population of HSCs limits growth of individual clones (75). This work provides some credence to the idea; however, the strength of this effect is not clear. Furthermore, its exact manifestation may be highly tissue specific, and thus the extent to which it might impede the transition from healthy tissue to malignancies remains an open question.

## Methods

### MLE of Logistic Trajectories

We fit all variant size trajectories reported by Fabre and colleagues (15) to the logistic function

$$x\left(t\right)=\frac{K}{1+\frac{K-x_{0}}{x_{0}}e^{-r_{\log}\left(t-t_{0}\right)}}$$ (B)

using an MLE, assuming each measurement of a VAF ${\tilde{x}}_{i}$ to be a binomial sampling, in which the success probability is given by the (unknown) true frequency $x_{i}$ and the number of trials is given by the coverage V of that site. Thus, for any proposed trajectory y(t|β) (where β is the set of parameters to fit), we computed the likelihood of obtaining the observed sequence of measurements ${\tilde{X}}_{i}={\tilde{x}}_{i}V$ at times $t_{i}$ as $L\left(\beta \right)=\prod_{i}P\left\{{\tilde{X}}_{i}\;|\;y\left(t_{i}|\beta \right),V\right\}$, where P is the associated binomial probability. This likelihood was then maximized with respect to β to obtain the best fit for each of the 3 trajectory types (increasing, decreasing, and saturating). For an increasing trajectory on a nonsex chromosome, we took K=0.5 and $x_{0}=1/2N$ as fixed parameters and assumed $r_{\log}>0$, maximizing with respect to $\beta =\left\{t_{0},r_{\log}\right\}$. For a decreasing trajectory, we took $\beta =\left\{t_{0},x_{0},r_{\log}\right\}$ (with $x_{0}$ the size at which the clone became negatively selected) and assumed $r_{\log}<0$. For the saturating trajectory, we assumed $r_{\log}=0$ and fit the single free parameter $\beta =\left\{x_{0}\right\}$. The trajectory with the highest maximum likelihood was then taken as the best fit.

### Numerical Simulations of the SDE System

To simulate a population of clones occurring with rate μ and obeying Equation A, we first draw the total number of equations K from a Poisson distribution with rate μT, draw their arrival times from the uniform distribution on [0,T] (this describes a Poisson process in time), and have newly introduced clones initiate at size 1/N. Upon introduction, a clone k has its fitness $s_{k}$ drawn from a gamma distribution $s_{k}∼\gamma \left(\frac{\eta^{2}}{\sigma^{2}},\frac{\sigma^{2}}{\eta }\right)$ with mean η and variance $\sigma^{2}$. Numerical solution trajectories of this system were calculated using the Julia computing language and the package “DifferentialEquations.jl” (76, 77).

### Simulating Trajectories for the Approximate Bayesian Computation

To construct a simulated dataset for ABC comparison of variant trajectories, 300 realizations of the SDE system were performed. Each run was measured at time T, and of the variants exceeding a detection threshold $x_{\min}=0.002$, only up to 3 were chosen for tracking to obtain a similar number of variants per donor in the data. Because trajectories in the dataset were captured through targeted sequencing of known driver genes, it is possible that the observed variants would be biased toward higher innate fitness compared with a randomly selected set of drivers, as the knowledge of a driver may correlate with its fitness. To account for this, we introduced another parameter q describing the probability of an observed driver possessing the highest innate fitness in the system. After ranking all variants in a simulated individual by their innate fitness, each of the observed variants has a probability q of being chosen in ranked order and a probability 1-q of being selected randomly. Thus, for q = 1, observed variants are maximally biased toward high innate fitness—i.e., sampled in order of innate fitness—whereas for q = 0 variant observation is entirely random. From the pooled set of selected variants across all simulated individuals, the size distribution was constructed by binning variant sizes at the time of measurement into 25 bins covering the full VAF range $\left[x_{\min}/2,0.5\right]$ (the size of a clone is twice its VAF). For the growth rate distribution, each sampled variant was measured at the 4 equidistant time points spanning 10 years centered around T: {T-5,T-1.66,T+1.66,T+1.66}. The maximum likelihood fitting performed to obtain all variants’ growth rates $r_{\log}$, which were partitioned into 50 equidistant bins constituting the logistic growth rate distribution. Because both the size- and growth-rate distributions are expected to change with the age of observed individuals, the donor dataset was partitioned into age groups covering 6-year spans, and the comparison with simulations was performed with T at the center of the respective age group. For example, variants measured between 70 and 76 years of age were compared with simulations measured at T=73 (Supplementary Fig. S3).

### Agent-Based Model with Neutral Mutation Accumulation

We implement the agent-based clonal competition model via the stochastic simulation algorithm [also referred to as the Gillespie algorithm (38)]. Each agent represents a cell with designated clone k and a set of mutations {u}. Changes to the system are performed as successive events, with the interevent times Δt drawn from an exponential distribution with the rate equal to the population-level division rate $\underset{\text{cell}\;i}{\sum }r_{i}$. The division rate of a cell is given by its clone k as $r_{k}=r_{WT}+s_{k}$ with $s_{k}$ representing the fitness increase compared with the WT. Starting from a single cell with no mutations, we assume a pure birth process—all events are cell divisions—during ontogenic growth, followed by a Moran process—events consist of a simultaneous birth and death (selected randomly from the population)—once the mature population size is reached. Upon proliferation, a new daughter cell inherits the mutations of its parent. It also inherits the clone of its parent or with probability  μΔt/N establishes a new fit clone the fitness $s_{k}$ of which is drawn from the population-level fitness distribution. After duplication, each daughter cell is assigned $m_{b}∼\text{Pois}\left(\mu_{b}\Delta t\right)$ mutations due to background mutational processes and $m_{d}∼\text{Pois}\left(\mu_{d}\right)$ mutations due to replication errors (see Supplementary Methods S5 and Supplementary Table S2 for more details).

### WGS of HSC Colonies

We obtained the WGS data published by Mitchell and colleagues (16) from Mendeley at https://data.mendeley.com/datasets/np54zjkvxr/2, from which we extracted the genotype matrices of all 9 individuals (3,046 cells). The SFSs were computed from the single-nucleotide variants and short indels present in the individual cells of the 9 donors (216–451 cells per donor; 362 cells on average) stored as the genotype matrices. We obtained the number of expanded clones per donor, defined as the number of postnatal ancestral lineages whose descendants contributed more than 1% of colonies at the time of sampling, from the phylogenies reconstructed by (16).

### Aggregated Trajectory Datasets

To investigate individual trajectories, we aggregated the target sequencing data generated by Fabre and colleagues (15), Robertson and colleagues (30), and van Zeventer and colleagues (33), all comprising longitudinal studies of DNA blood samples collected from a total of 1,969 individuals with no history of hematologic malignancy. To ensure consistency across data, variants with a frequency smaller than 1% VAF were removed from Fabre and colleagues (15) and van Zeventer and colleagues (33), whereas a stricter threshold of <1.5% VAF was adopted for Robertson and colleagues (30), due to excessive noise in these data. In total, this resulted in 1,942 viable donors across all data sets.

### Bayesian Inference on Individual Trajectories

To estimate the innate fitness $\tilde{s}$ and arrival time $\tilde{t_{0}}$ of a single trajectory, we performed an ABC comparing the target trajectory with an ensemble of trajectories simulated from the SDE model (1) with the best-fit parameters obtained from the parameter-fitting ABC. Simulated trajectories were generated by inserting 1 single-celled clone with a predetermined fitness $s_{i}$ and arrival time $t_{i}$ in a simulation of the SDE. Drawing all $s_{i}$ and $t_{i}$ from uniform prior distributions s∼U(0.01,2) and t∼U(0,90), we obtained an ensemble of 150,000 independently simulated trajectories. For a target trajectory $\tilde{x}=\left\{{\tilde{x}}_{j}\right\}$ measured at ages $\left\{z_{j}\right\}$, the distance from a reference trajectory $x_{i}\left(t\right)$ was calculated as the Euclidean distance $d_{i}=\left|\tilde{x}-x_{i}\right|$ from the reference measured at the same times $x_{i}=\left\{x_{i}\left(z_{j}\right)\right\}$. To generate the posterior distribution, we retained only trajectories with distances up to the 150th order statistic (0.1% of all trajectories). As estimators for $\tilde{s}$ and $\tilde{t_{0}}$, we took the maxima of a kernel density estimate of the marginalized posteriors. See also Supplementary Methods S7, for more details.

## Supplementary Material

Supplementary Methods 1 Description of the mathematical and computational methods

Supplementary Table 1 Supplementary Table 1: Inferred innate fitness of commonly occurring variants.

Supplementary Table 2 Supplementary Table 2: Parameters of the agent-based model. Parameters were inferred either through the ABC framework or by regression of the mean of the number of mutations.

Supplementary Figures S1-S22 Figure S1. Model dynamics testing and exploration. Figure S2. The dynamics of the simulated site frequency spectrum (SFS). Figure S3. Transitions of HSC site frequency spectrum (SFS) with age in Mitchell et al. data. Figure S4. Zoomed-in HSC site frequency spectrum (SFS) in Mitchell et al. data. Figure S5. Agent-based model parametrisation with the single-cell mutational burden in Mitchell et al. data. Figure S6. Summary statistics of the approximate Bayesian computation on the SFS and the number of detectable expanded clones from Mitchell et al. data. Figure S7. Validation of approximate Bayesian computation on variant trajectories from simulated datasets. Figure S8. Validation of approximate Bayesian computation on SFS and number of clones on simulated data. Figure S9. Parameter posterior distributions obtained via approximate Bayesian computation on time-resolved clone trajectories from Fabre et al. donor data. Figure S10. Parameter posterior distributions obtained via approximate Bayesian computation on the SFS and the number of detectable expanded clones from Mitchell et al. data. Figure S11. The SFS of the simulations closest to the data according to the SFS and the number of expanded clones summary statistics. Figure S12. Sensitivity analysis for the posterior distributions inferred from the time-resolved clone trajectories from Fabre et al. data. Figure S13. Sensitivity analysis for the posterior distributions inferred from the SFS and the number of detectable expanded clones from Mitchell et al. data. Figure S14. Parameter posterior distributions obtained via approximate Bayesian computation on time-resolved clone trajectories from Fabre et al. donor data with extended prior for µ. Figure S15. Parameter posterior distributions obtained via approximate Bayesian computation on the SFS and the number of detectable expanded clones from Mitchell et al. data using a wider prior distribution for the arrival rate µ. Figure S16. Parameter posterior distributions including the total HSC population size obtained via approximate Bayesian computation on time-resolved clone trajectories from Fabre et al. donor data. Figure S17. Parameter posterior distributions obtained via approximate Bayesian computation on time-resolved clone trajectories from Fabre et al. donor data, assuming known values of N and τ. Figure S18. Posterior distributions obtained via approximate Bayesian computation on the SFS and the number of detectable expanded clones from Mitchell et al. data using fixed values for N and τ. Figure S19. Aggregating three target sequencing datasets to study haematopoiesis in 1,942 individuals. Figure S20. The mean number of detectable clones with age measured in simulations of the SDE model for various values of the clone arrival rate µ (gray lines) alongside the number observed in data (blue lines). Figure S21. p-values for the Anderson-Darling and Kolmogorov-Smirnov tests when comparing the arrival-time distributions of high-fitness (s > sT) trajectories observed in the data and simulated multistep clones. Figure S22. Unconstrained growth of clones leads to exponentially increasing stem cell populations.

## Data Availability

Publicly available data generated by others were used by the authors. The data analyzed in this study were obtained from the following repositories: Figshare: https://figshare.com/articles/dataset/Data/15029118. Mendeley: https://data.mendeley.com/datasets/np54zjkvxr/2. NCBI Gene Expression Omnibus: https://www.ncbi.nlm.nih.gov/geo/query/acc.cgi?acc=GSE178936. The other dataset analyzed in this study is available from Lifelines at https://www.lifelines-biobank.com/. Restrictions apply to the availability of these data, which were used under license for this study and downloaded from the European Genome-phenome Archive at https://ega-archive.org/datasets/EGAD00001010144 and https://ega-archive.org/datasets/EGAD00001010145.

## Code Availability

The HSC dynamics are implemented in the Rust programming language, and the code is available at https://github.com/fraterenz/hsc. The numerical implementation of the SDE system in Equation A was written in the Julia programming language and is available at https://github.com/natevmp/competitive-selection. The Python code to recreate the figures is hosted at https://github.com/fraterenz/hsc-figures/tree/master/inferences.

## References

[1] Blokzijl F , de Ligt J, Jager M, Sasselli V, Roerink S, Sasaki N, . Tissue-specific mutation accumulation in human adult stem cells during life. Nature 2016;538:260–4. 10.1038/nature19768. 27698416 PMC5536223

[2] Lee-Six H , Øbro NF, Shepherd MS, Grossmann S, Dawson K, Belmonte M, . Population dynamics of normal human blood inferred from somatic mutations. Nature 2018;561:473–8. 10.1038/s41586-018-0497-0. 30185910 PMC6163040

[3] Martincorena I , Fowler JC, Wabik A, Lawson ARJ, Abascal F, Hall MWJ, . Somatic mutant clones colonize the human esophagus with age. Science 2018;362:911–7. 10.1126/science.aau3879. 30337457 PMC6298579

[4] Werner B , Case J, Williams MJ, Chkhaidze K, Temko D, Fernández-Mateos J, . Measuring single cell divisions in human tissues from multi-region sequencing data. Nat Commun 2020;11:1035. 10.1038/s41467-020-14844-6. 32098957 PMC7042311

[5] Abascal F , Harvey LMR, Mitchell E, Lawson ARJ, Lensing SV, Ellis P, . Somatic mutation landscapes at single-molecule resolution. Nature 2021;593:405–10. 10.1038/s41586-021-03477-4. 33911282

[6] Martincorena I . Somatic mutation and clonal expansions in human tissues. Genome Med 2019;11:35. 10.1186/s13073-019-0648-4. 31138277 PMC6537184

[7] Watson CJ , Papula AL, Poon GYP, Wong WH, Young AL, Druley TE, . The evolutionary dynamics and fitness landscape of clonal hematopoiesis. Science 2020;367:1449–54. 10.1126/science.aay9333. 32217721

[8] Poon GYP , Watson CJ, Fisher DS, Blundell JR. Synonymous mutations reveal genome-wide levels of positive selection in healthy tissues. Nat Genet 2021;53:1597–605. 10.1038/s41588-021-00957-1. 34737428

[9] Jassim A , Rahrmann EP, Simons BD, Gilbertson RJ. Cancers make their own luck: theories of cancer origins. Nat Rev Cancer 2023;23:710–24. 10.1038/s41568-023-00602-5. 37488363

[10] Moeller ME , Mon Père NV, Werner B, Huang W. Measures of genetic diversification in somatic tissues at bulk and single-cell resolution. Elife 2024;12:RP89780. 10.7554/eLife.89780. 38265286 PMC10945735

[11] Jaiswal S , Fontanillas P, Flannick J, Manning A, Grauman PV, Mar BG, . Age-related clonal hematopoiesis associated with adverse outcomes. N Engl J Med 2014;371:2488–98. 10.1056/NEJMoa1408617. 25426837 PMC4306669

[12] Genovese G , Kähler AK, Handsaker RE, Lindberg J, Rose SA, Bakhoum SF, . Clonal hematopoiesis and blood-cancer risk inferred from blood DNA sequence. N Engl J Med 2014;371:2477–87. 10.1056/NEJMoa1409405. 25426838 PMC4290021

[13] Zink F , Stacey SN, Norddahl GL, Frigge ML, Magnusson OT, Jonsdottir I, . Clonal hematopoiesis, with and without candidate driver mutations, is common in the elderly. Blood 2017;130:742–52. 10.1182/blood-2017-02-769869. 28483762 PMC5553576

[14] Jaiswal S , Ebert BL. Clonal hematopoiesis in human aging and disease. Science 2019;366:eaan4673. 10.1126/science.aan4673. 31672865 PMC8050831

[15] Fabre MA , de Almeida JG, Fiorillo E, Mitchell E, Damaskou A, Rak J, . The longitudinal dynamics and natural history of clonal haematopoiesis. Nature 2022;606:335–42. 10.1038/s41586-022-04785-z. 35650444 PMC9177423

[16] Mitchell E , Spencer Chapman M, Williams N, Dawson KJ, Mende N, Calderbank EF, . Clonal dynamics of haematopoiesis across the human lifespan. Nature 2022;606:343–50. 10.1038/s41586-022-04786-y. 35650442 PMC9177428

[17] Bowman RL , Busque L, Levine RL. Clonal hematopoiesis and evolution to hematopoietic malignancies. Cell Stem Cell 2018;22:157–70. 10.1016/j.stem.2018.01.011. 29395053 PMC5804896

[18] Niroula A , Sekar A, Murakami MA, Trinder M, Agrawal M, Wong WJ, . Distinction of lymphoid and myeloid clonal hematopoiesis. Nat Med 2021;27:1921–7. 10.1038/s41591-021-01521-4. 34663986 PMC8621497

[19] Jaiswal S , Natarajan P, Silver AJ, Gibson CJ, Bick AG, Shvartz E, . Clonal hematopoiesis and risk of atherosclerotic cardiovascular disease. N Engl J Med 2017;377:111–21. 10.1056/NEJMoa1701719. 28636844 PMC6717509

[20] Wong WJ , Emdin C, Bick AG, Zekavat SM, Niroula A, Pirruccello JP, . Clonal haematopoiesis and risk of chronic liver disease. Nature 2023;616:747–54. 10.1038/s41586-023-05857-4. 37046084 PMC10405350

[21] Kessler MD , Damask A, O’Keeffe S, Banerjee N, Li D, Watanabe K, . Common and rare variant associations with clonal haematopoiesis phenotypes. Nature 2022;612:301–9. 10.1038/s41586-022-05448-9. 36450978 PMC9713173

[22] Pich O , Bernard E, Zagorulya M, Rowan A, Pospori C, Slama R, . Tumor-infiltrating clonal hematopoiesis. N Engl J Med 2025;392:1594–608. 10.1056/NEJMoa2413361. 40267425 PMC12021423

[23] Coombs CC , Zehir A, Devlin SM, Kishtagari A, Syed A, Jonsson P, . Therapy-related clonal hematopoiesis in patients with non-hematologic cancers is common and associated with adverse clinical outcomes. Cell Stem Cell 2017;21:374–82.e4. 10.1016/j.stem.2017.07.010. 28803919 PMC5591073

[24] Spencer Chapman M , Cull AH, Ciuculescu MF, Esrick EB, Mitchell E, Jung H, . Clonal selection of hematopoietic stem cells after gene therapy for sickle cell disease. Nat Med 2023;29:3175–83. 10.1038/s41591-023-02636-6. 37973947 PMC10719109

[25] Spencer Chapman M , Wilk CM, Boettcher S, Mitchell E, Dawson K, Williams N, . Clonal dynamics after allogeneic haematopoietic cell transplantation. Nature 2024;635:926–34. 10.1038/s41586-024-08128-y. 39478227 PMC11602715

[26] Bolton KL , Ptashkin RN, Gao T, Braunstein L, Devlin SM, Kelly D, . Cancer therapy shapes the fitness landscape of clonal hematopoiesis. Nat Genet 2020;52:1219–26. 10.1038/s41588-020-00710-0. 33106634 PMC7891089

[27] Martincorena I , Roshan A, Gerstung M, Ellis P, Van Loo P, McLaren S, . Tumor evolution. High burden and pervasive positive selection of somatic mutations in normal human skin. Science 2015;348:880–6. 10.1126/science.aaa6806. 25999502 PMC4471149

[28] Abby E , Dentro SC, Hall MW, Fowler JC, Ong SH, Sood R, . Notch1 mutations drive clonal expansion in normal esophageal epithelium but impair tumor growth. Nat Genet 2023;55:232–45. 10.1038/s41588-022-01280-z. 36658434 PMC9925379

[29] Lawson AR , Abascal F, Nicola PA, Lensing SV, Roberts AL, Kalantzis G, . Somatic mutation and selection at population scale. Nature 2025;647:411–20. 10.1038/s41586-025-09584-w. 41062696 PMC12611758

[30] Robertson NA , Latorre-Crespo E, Terradas-Terradas M, Lemos-Portela J, Purcell AC, Livesey BJ, . Longitudinal dynamics of clonal hematopoiesis identifies gene-specific fitness effects. Nat Med 2022;28:1439–46. 10.1038/s41591-022-01883-3. 35788175 PMC9307482

[31] Ewens WJ . Mathematical population genetics: theoretical introduction. New York (NY): Springer; 2004. p. 11–3.

[32] Czuppon P , Traulsen A. Understanding evolutionary and ecological dynamics using a continuum limit. Ecol Evol 2021;11:5857–73. 10.1002/ece3.7205. 34141189 PMC8207364

[33] van Zeventer IA , de Graaf AO, Salzbrunn JB, Nolte IM, Kamphuis P, Dinmohamed A, . Evolutionary landscape of clonal hematopoiesis in 3,359 individuals from the general population. Cancer Cell 2023;41:1017–31.e4. 10.1016/j.ccell.2023.04.006. 37146604

[34] Armitage P , Doll R. The age distribution of cancer and a multi-stage theory of carcinogenesis. Br J Cancer 2004;91:1983–9. 10.1038/sj.bjc.6602297. 15599380 PMC2410148

[35] Takeishi S , Marchand T, Koba WR, Borger DK, Xu C, Guha C, . Haematopoietic stem cell number is not solely defined by niche availability. Nature 2025;646:687–96. 10.1038/s41586-025-09462-5. 40866696 PMC12527909

[36] Page KM , Nowak MA. Unifying evolutionary dynamics. J Theor Biol 2002;219:93–8. 10.1006/jtbi.2002.3112. 12392978

[37] Volterra V . Fluctuations in the abundance of a species considered mathematically. Nature 1926;118:558–60. 10.1038/118558a0.

[38] Gillespie DT . Stochastic simulation of chemical kinetics. Annu Rev Phys Chem 2007;58:35–55. 10.1146/annurev.physchem.58.032806.104637. 17037977

[39] Lang GI , Rice DP, Hickman MJ, Sodergren E, Weinstock GM, Botstein D, . Pervasive genetic hitchhiking and clonal interference in forty evolving yeast populations. Nature 2013;500:571–4. 10.1038/nature12344. 23873039 PMC3758440

[40] Durrett R . Population genetics of neutral mutations in exponentially growing cancer cell populations. Ann Appl Probab 2013;23:230–50. 10.1214/11-aap824. 23471293 PMC3588108

[41] Williams MJ , Werner B, Barnes CP, Graham TA, Sottoriva A. Identification of neutral tumor evolution across cancer types. Nat Genet 2016;48:238–44. 10.1038/ng.3489. 26780609 PMC4934603

[42] Gunnarsson EB , Leder K, Foo J. Exact site frequency spectra of neutrally evolving tumors: a transition between power laws reveals a signature of cell viability. Theor Popul Biol 2021;142:67–90. 10.1016/j.tpb.2021.09.004. 34560155

[43] Stein A , Werner B. On the patterns of genetic intra-tumor heterogeneity before and after treatment. Genetics 2025;230:iyaf101. 10.1093/genetics/iyaf101. 40439127 PMC12341898

[44] Williams MJ , Werner B, Heide T, Curtis C, Barnes CP, Sottoriva A, . Quantification of subclonal selection in cancer from bulk sequencing data. Nat Genet 2018;50:895–903. 10.1038/s41588-018-0128-6. 29808029 PMC6475346

[45] Catlin SN , Busque L, Gale RE, Guttorp P, Abkowitz JL. The replication rate of human hematopoietic stem cells in vivo. Blood 2011;117:4460–6. 10.1182/blood-2010-08-303537. 21343613 PMC3099568

[46] Csilléry K , Blum MGB, Gaggiotti OE, François O. Approximate Bayesian Computation (ABC) in practice. Trends Ecol Evol 2010;25:410–8. 10.1016/j.tree.2010.04.001. 20488578

[47] Watson CJ , Blundell JR. Mutation rates and fitness consequences of mosaic chromosomal alterations in blood. Nat Genet 2023;55:1677–85. 10.1038/s41588-023-01490-z. 37697102 PMC10562253

[48] Bernstein N , Spencer Chapman M, Nyamondo K, Chen Z, Williams N, Mitchell E, . Analysis of somatic mutations in whole blood from 200,618 individuals identifies pervasive positive selection and novel drivers of clonal hematopoiesis. Nat Genet 2024;56:1147–55. 10.1038/s41588-024-01755-1. 38744975 PMC11176083

[49] Pich O , Reyes-Salazar I, Gonzalez-Perez A, Lopez-Bigas N. Discovering the drivers of clonal hematopoiesis. Nat Commun 2022;13:4267. 10.1038/s41467-022-31878-0. 35871184 PMC9308779

[50] Pospisil DA , Bair W. The unbiased estimation of the fraction of variance explained by a model. PLoS Comput Biol 2021;17:e1009212. 10.1371/journal.pcbi.1009212. 34347786 PMC8367013

[51] Niederwieser D , Baldomero H, Bazuaye N, Bupp C, Chaudhri N, Corbacioglu S, . One and a half million hematopoietic stem cell transplants: continuous and differential improvement in worldwide access with the use of non-identical family donors. Haematologica 2022;107:1045–53. 10.3324/haematol.2021.279189. 34382386 PMC9052915

[52] Aubert G , Lansdorp PM. Telomeres and aging. Physiol Rev 2008;88:557–79. 10.1152/physrev.00026.2007. 18391173

[53] Werner B , Beier F, Hummel S, Balabanov S, Lassay L, Orlikowsky T, . Reconstructing the in vivo dynamics of hematopoietic stem cells from telomere length distributions. Elife 2015;4:e08687. 10.7554/eLife.08687. 26468615 PMC4744200

[54] Brümmendorf TH , Maciejewski JP, Mak J, Young NS, Lansdorp PM. Telomere length in leukocyte subpopulations of patients with aplastic anemia. Blood 2001;97:895–900. 10.1182/blood.v97.4.895. 11159514

[55] Kamizela AE , Leongamornlert D, Williams N, Wang X, Nyamondo K, Dawson K, . Timing and trajectory of BCR::ABL1-driven chronic myeloid leukaemia. Nature 2025;640:982–90. 10.1038/s41586-025-08817-2. 40205062 PMC12018454

[56] Gabbutt C , Duran-Ferrer M, Grant HE, Mallo D, Nadeu F, Househam J, . Fluctuating DNA methylation tracks cancer evolution at clinical scale. Nature 2025;645:764–73. 10.1038/s41586-025-09374-4. 40931062 PMC12443617

[57] Swann JB , Smyth MJ. Immune surveillance of tumors. J Clin Invest 2007;117:1137–46. 10.1172/JCI31405. 17476343 PMC1857231

[58] Martincorena I , Raine KM, Gerstung M, Dawson KJ, Haase K, Van Loo P, . Universal patterns of selection in cancer and somatic tissues. Cell 2017;171:1029–41.e21. 10.1016/j.cell.2017.09.042. 29056346 PMC5720395

[59] Weghorn D , Sunyaev S. Bayesian inference of negative and positive selection in human cancers. Nat Genet 2017;49:1785–8. 10.1038/ng.3987. 29106416

[60] Geiger H , De Haan G, Florian MC. The ageing haematopoietic stem cell compartment. Nat Rev Immunol 2013;13:376–89. 10.1038/nri3433. 23584423

[61] de Haan G , Lazare SS. Aging of hematopoietic stem cells. Blood 2018;131:479–87. 10.1182/blood-2017-06-746412. 29141947

[62] Vasto S , Candore G, Balistreri CR, Caruso M, Colonna-Romano G, Grimaldi MP, . Inflammatory networks in ageing, age-related diseases and longevity. Mech Ageing Dev 2007;128:83–91. 10.1016/j.mad.2006.11.015. 17118425

[63] Caiado F , Pietras EM, Manz MG. Inflammation as a regulator of hematopoietic stem cell function in disease, aging, and clonal selection. J Exp Med 2021;218:e20201541. 10.1084/jem.20201541. 34129016 PMC8210622

[64] Jakobsen NA , Turkalj S, Zeng AG, Stoilova B, Metzner M, Rahmig S, . Selective advantage of mutant stem cells in human clonal hematopoiesis is associated with attenuated response to inflammation and aging. Cell Stem Cell 2024;31:1127–44.e17. 10.1016/j.stem.2024.05.010. 38917807 PMC11512683

[65] Kucab JE , Zou X, Morganella S, Joel M, Nanda AS, Nagy E, . A compendium of mutational signatures of environmental agents. Cell 2019;177:821–36.e16. 10.1016/j.cell.2019.03.001. 30982602 PMC6506336

[66] Hill W , Lim EL, Weeden CE, Lee C, Augustine M, Chen K, . Lung adenocarcinoma promotion by air pollutants. Nature 2023;616:159–67. 10.1038/s41586-023-05874-3. 37020004 PMC7614604

[67] Bick AG , Weinstock JS, Nandakumar SK, Fulco CP, Bao EL, Zekavat SM, . Inherited causes of clonal haematopoiesis in 97,691 whole genomes. Nature 2020;586:763–8. 10.1038/s41586-020-2819-2. 33057201 PMC7944936

[68] Liu J , Tran D, Xue L, Wiley BJ, Vlasschaert C, Watson CJ, . Germline genetic variation impacts clonal hematopoiesis landscape and progression to malignancy. Nat Genet 2025;57:1872–80. 10.1038/s41588-025-02250-x. 40664769 PMC12339400

[69] Izzo F , Lee SC, Poran A, Chaligne R, Gaiti F, Gross B, . DNA methylation disruption reshapes the hematopoietic differentiation landscape. Nat Genet 2020;52:378–87. 10.1038/s41588-020-0595-4. 32203468 PMC7216752

[70] Yang Y , Cathelin S, Liu AC, Subedi A, Maher A, Hosseini M, . TET2 deficiency increases the competitive advantage of hematopoietic stem and progenitor cells through upregulation of thrombopoietin receptor signaling. Nat Commun 2025;16:2384. 10.1038/s41467-025-57614-y. 40064887 PMC11894142

[71] Nam AS , Dusaj N, Izzo F, Murali R, Myers RM, Mouhieddine TH, . Single-cell multi-omics of human clonal hematopoiesis reveals that DNMT3A R882 mutations perturb early progenitor states through selective hypomethylation. Nat Genet 2022;54:1514–26. 10.1038/s41588-022-01179-9. 36138229 PMC10068894

[72] Cortés-López M , Chamely P, Hawkins AG, Stanley RF, Swett AD, Ganesan S, . Single-cell multi-omics defines the cell-type-specific impact of splicing aberrations in human hematopoietic clonal outgrowths. Cell Stem Cell 2023;30:1262–81.e8. 10.1016/j.stem.2023.07.012. 37582363 PMC10528176

[73] McLoughlin MA , Cheloor Kovilakam S, Dunn WG, Gu M, Tobin J, Pershad Y, . Telomere attrition becomes an instrument for clonal selection in aging hematopoiesis and leukemogenesis. Nat Genet 2025;57:2215–25. 10.1038/s41588-025-02296-x. 40877435 PMC12425810

[74] Papaemmanuil E , Gerstung M, Bullinger L, Gaidzik VI, Paschka P, Roberts ND, . Genomic classification and prognosis in acute myeloid leukemia. N Engl J Med 2016;374:2209–21. 10.1056/NEJMoa1516192. 27276561 PMC4979995

[75] Rahal Z , Scheet P, Kadara H. Somatic mutations in normal tissues: calm before the storm. Cancer Discov 2024;14:605–9. 10.1158/2159-8290.CD-23-1508. 38571416 PMC11234823

[76] Rackauckas C , Nie Q. DifferentialEquations.jl – a performant and feature-rich ecosystem for solving differential equations in Julia. J Open Res Softw 2017;5:15. 10.5334/jors.151.

[77] Rackauckas C , Nie Q. Adaptive methods for stochastic differential equations via natural embeddings and rejection sampling with memory. Discrete Continuous Dyn Syst Ser B 2017;22:2731–61. 10.3934/dcdsb.2017133. PMC5844583 29527134

