# Supplementary material for: The Evolution of Polyclonal Competition in Aging Hematopoiesis
Source: Cancer Discov. 2026 May 5;16(9):1765–79. doi: 10.1158/2159-8290.CD-25-0990 (PMC13531000; doi:10.1158/2159-8290.CD-25-0990)
Supplement: Supplementary file 1 [file cd-25-0990_supplementary_figures_s1-s22_suppsf1.pdf]

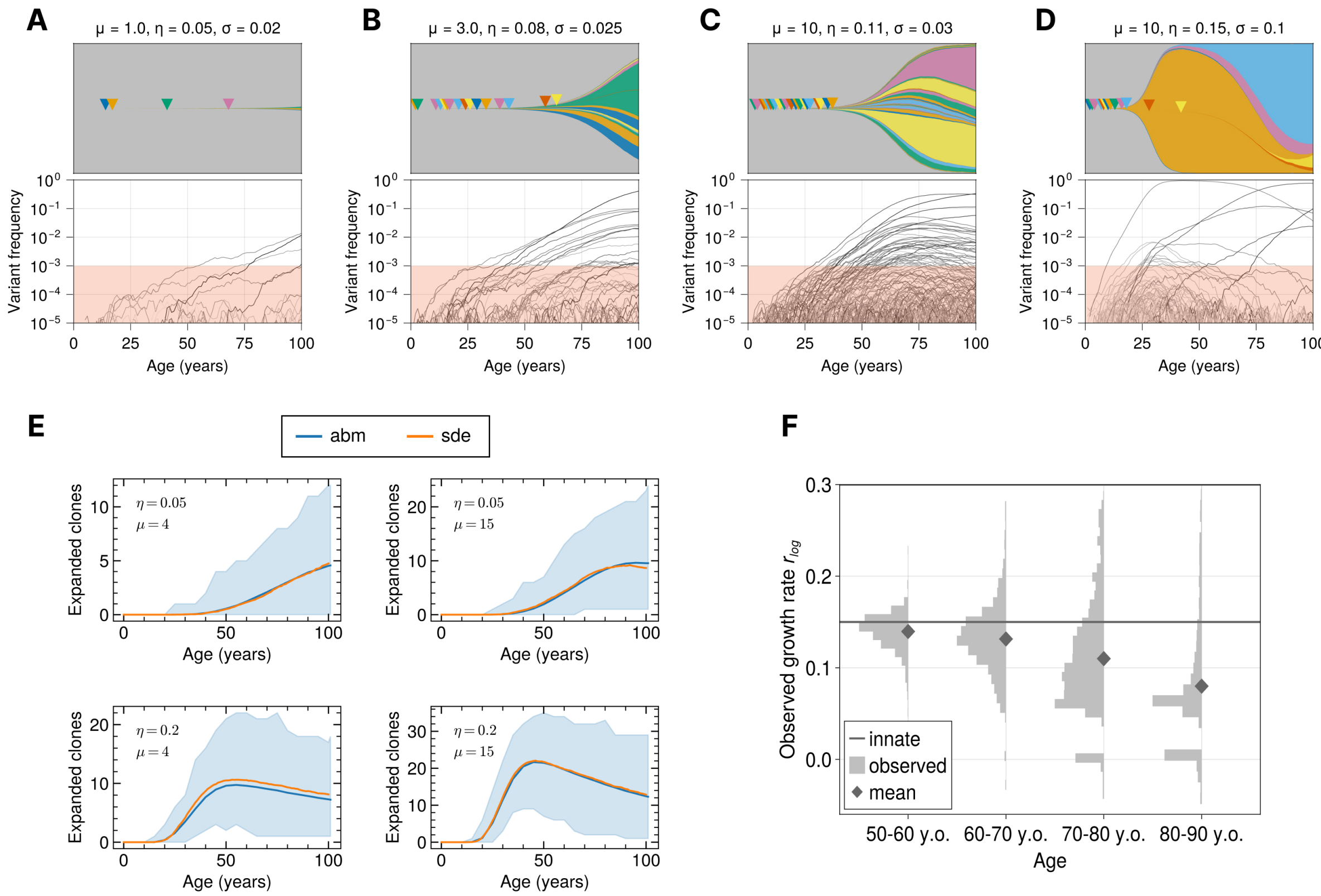

Figure S1. Model dynamics testing and exploration. A-D) Example realizations of the SDE model (Equation (SE19)) for different parameter combinations. In the Muller plots (top row) the mutant arrival times are indicated by triangles. **E)** The number of detectably expanded (>0.5%) clones with age averaged over 500 simulations agrees between the SDE (2.1) and ABM (4.2) implementations of the model. The blue shaded area shows the minimum and maximal values of the expanded clones for the ABM model. **F)** In 10,000 simulations a variant with innate fitness $s = 0.15$ is introduced at age 10 and observed at different time points later in life. The distribution of measured growth rates becomes wider and its mean decreases with age.

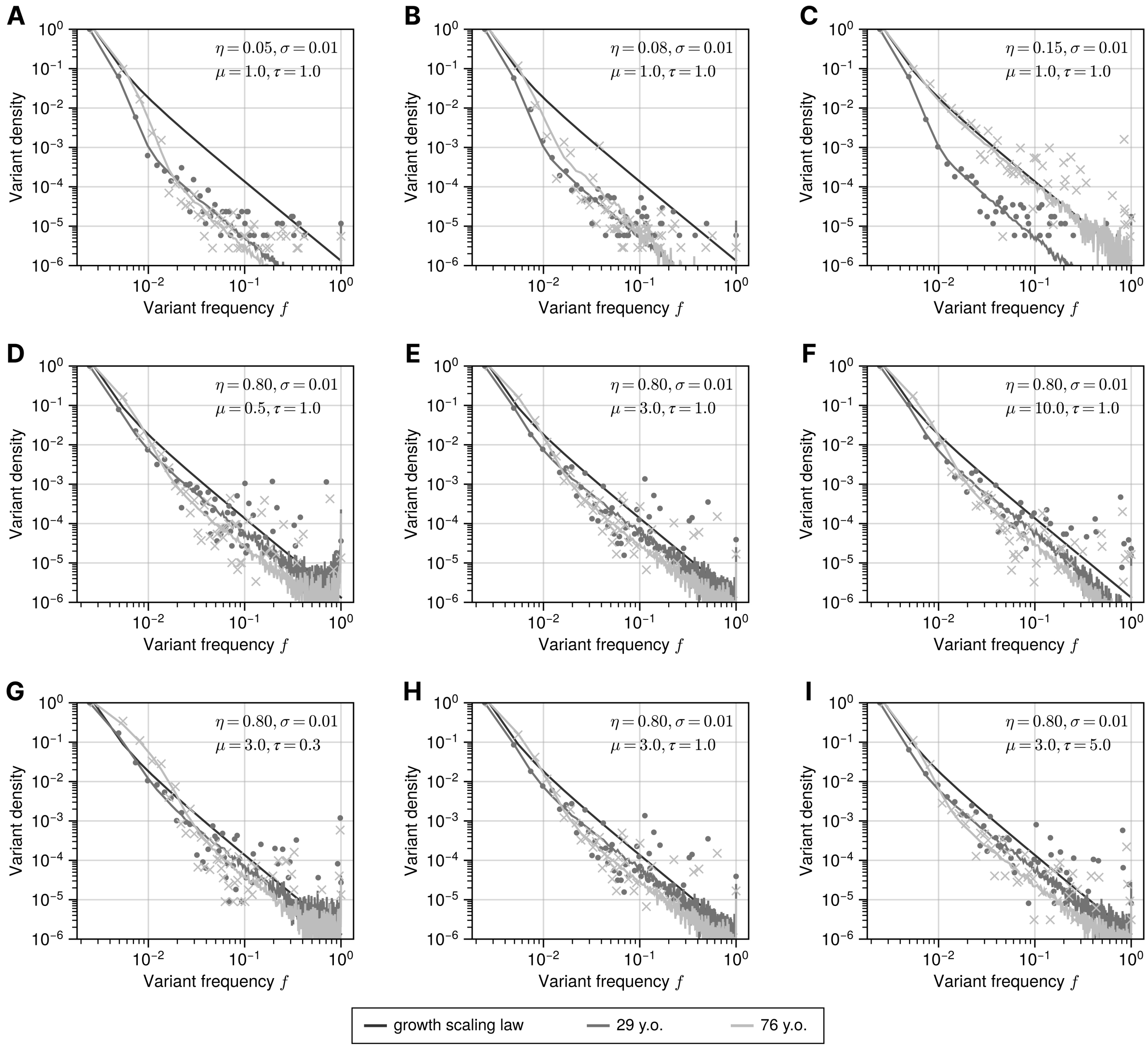

Figure S2. The dynamics of the simulated site frequency spectrum (SFS). Simulated SFS constructed by taking a sample of size 407 cells and 367 cells from population of 10^5 HSCs at age 29 and 76 respectively. Grey lines show the SFS averaged over 500 simulations, dots and crosses show individual simulations, and the solid black line shows the growth scaling law considering 367 cells. **A-C)** The dynamics of the SFS varying the value of the mean of the Gamma distribution of fitness effects η. **D-F)** The dynamics of the SFS varying the value of the arrival rate of fit clones per year μ. **G-I)** The dynamics of the SFS varying the value of the inter-division time for of wild-type cells per year τ.

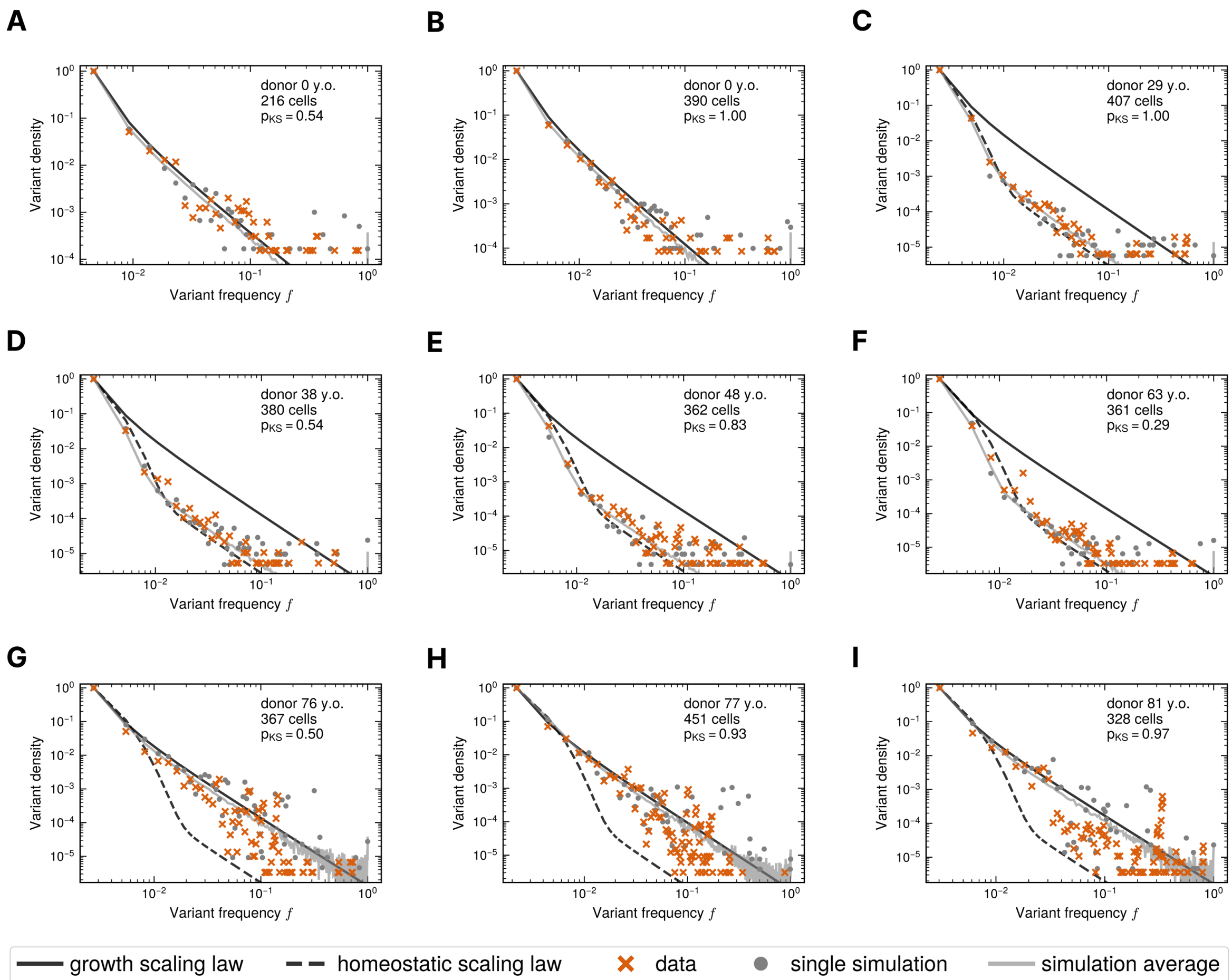

Figure S3. Transitions of HSC site frequency spectrum (SFS) with age in Mitchell et al. data. The SFS computed from 216-451 whole genome sequenced single cells (orange crosses), from a single realization of the agent-based simulation (gray dots), from averages over 500 simulations (gray line), from the expected scaling of a growing population (black solid line) and from the expected scaling of a homeostatic population of constant size (black dashed line). **A-B)** The SFS follows the expected scaling of a growing population for the two newborns. **C-F)** The SFS transitions towards the expected scaling of a homeostatic population of constant size. **G-I)** The SFS shifts back towards the expected scaling of a growing population at older ages.

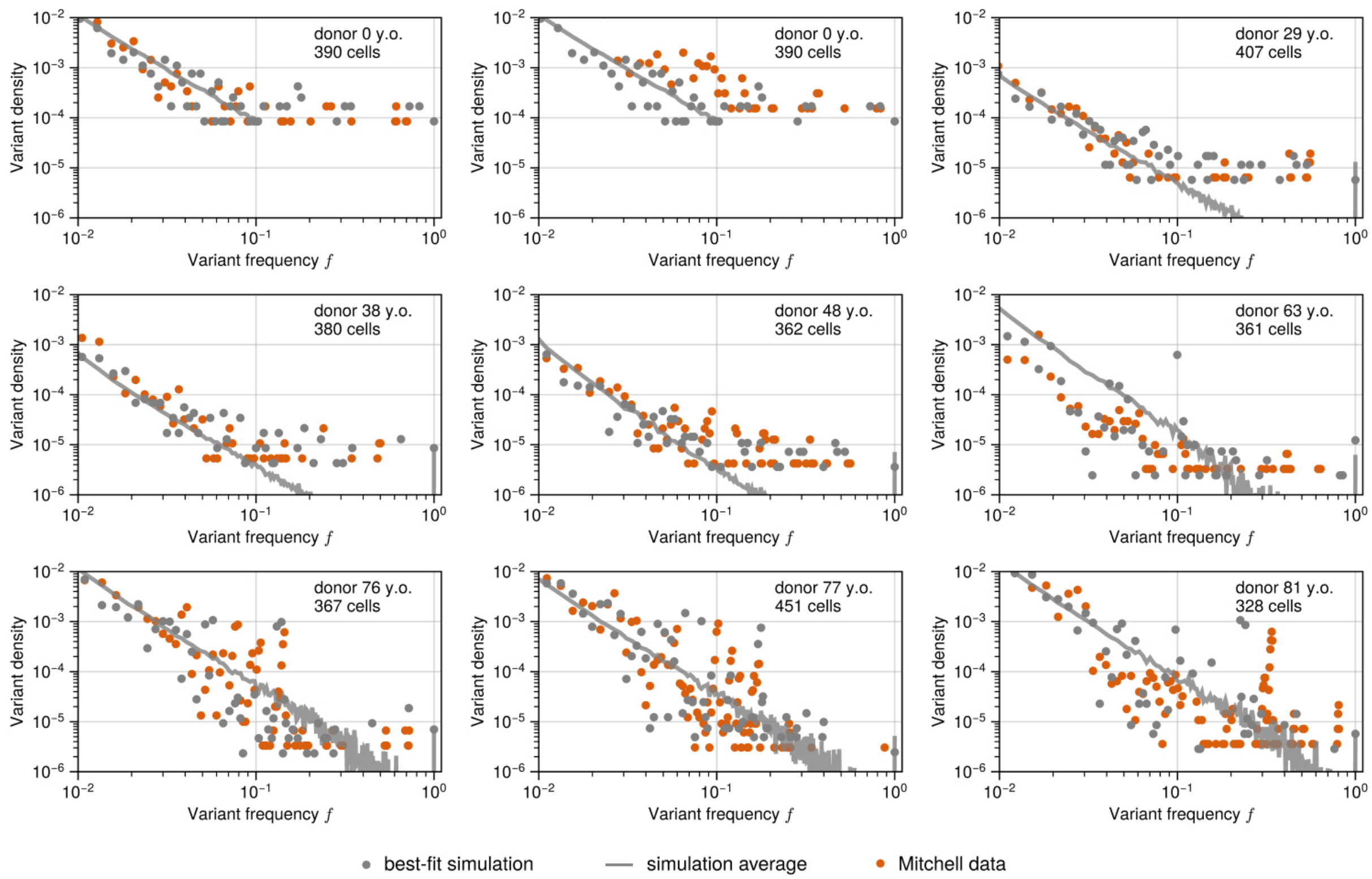

Figure S4. Zoomed-in HSC site frequency spectrum (SFS) in Mitchell et al. data. The zoomed-in (y-axis starts at frequency of 10^{-2}) SFS computed from 216-451 whole genome sequenced single cells (orange dots), from a single realization of the agent-based simulation (gray dots) and from averages over 500 simulations (gray line), see also Figure S3. The SFS of the newborns (first two panels) reaches minimal y-axis frequencies of approximately 10^{-4}, as newborns have fewer mutations compared to older donors.

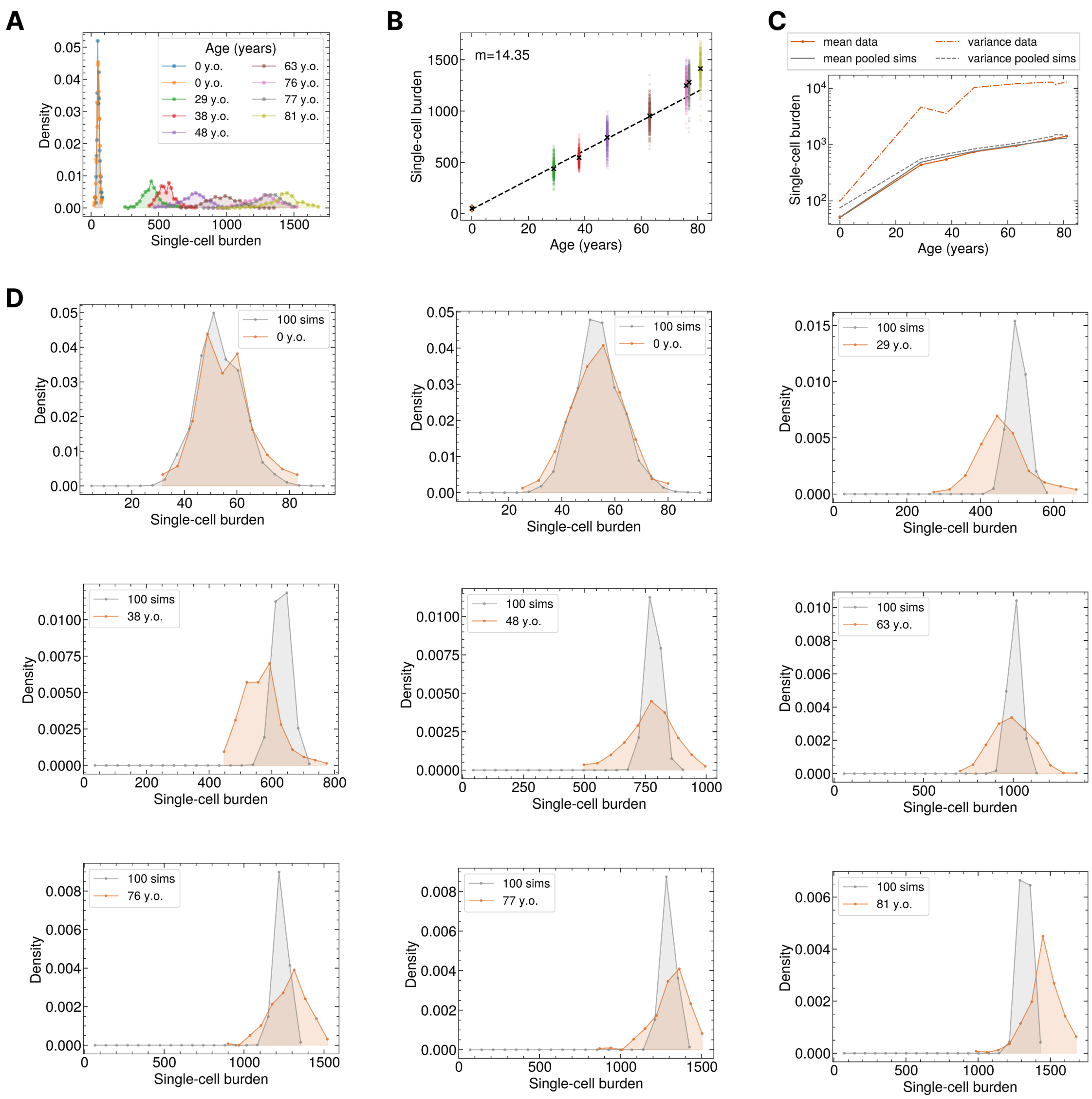

Figure S5. Agent-based model parametrisation with the single-cell mutational burden in Mitchell et al. data. A) Distribution of the number of mutations (indels and single-nucleotides) in individual cells across the donor cohort. **B)** Estimation of the average burden increase per year considering only donors without any clone at frequency greater than 1%. **C)** The mean (solid lines) and the variance (dashed lines) of the single-cell mutational burden in the data (orange) and those computed from 100 pooled simulations (gray). **D)** The single-cell mutational burden distributions in the data (orange) and those computed from 100 pooled simulations (gray).

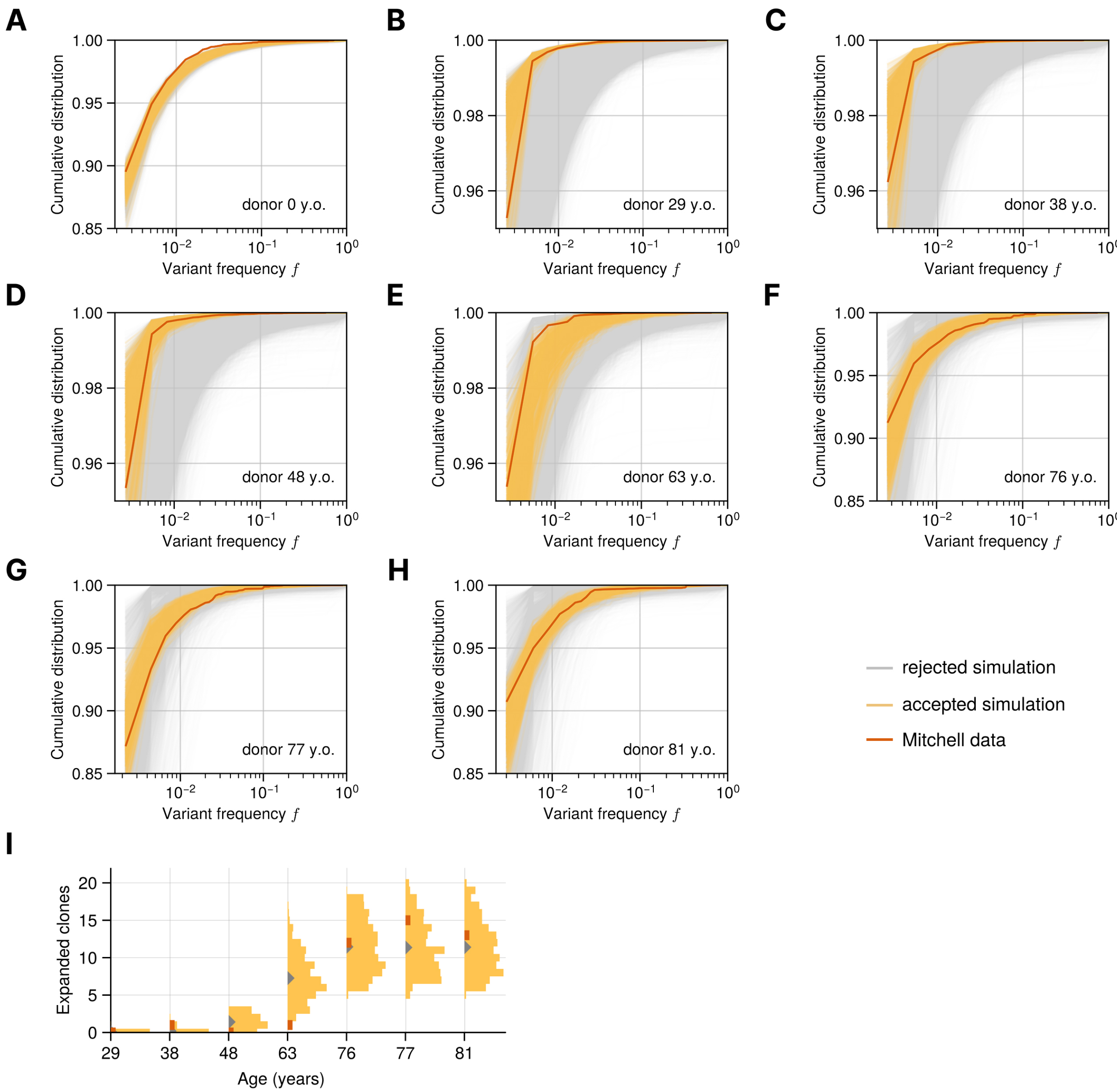

Figure S6. Summary statistics of the approximate Bayesian computation on the SFS and the number of detectable expanded clones from Mitchell et al. data. A-H) The cumulative distribution (cdf) computed from the site frequency spectra of the data (dots) is compared against the simulations. The simulations that are close to the data according to the average distance in the cdf for 7 out of 8 donors are kept (yellow) as samples of the posterior distributions. The remaining simulations (gray) are discarded. **I)** The number of clones with frequency $\geq 1\%$ in the data (orange squares) and those in the accepted simulations (in yellow, their means are shown with gray triangles).

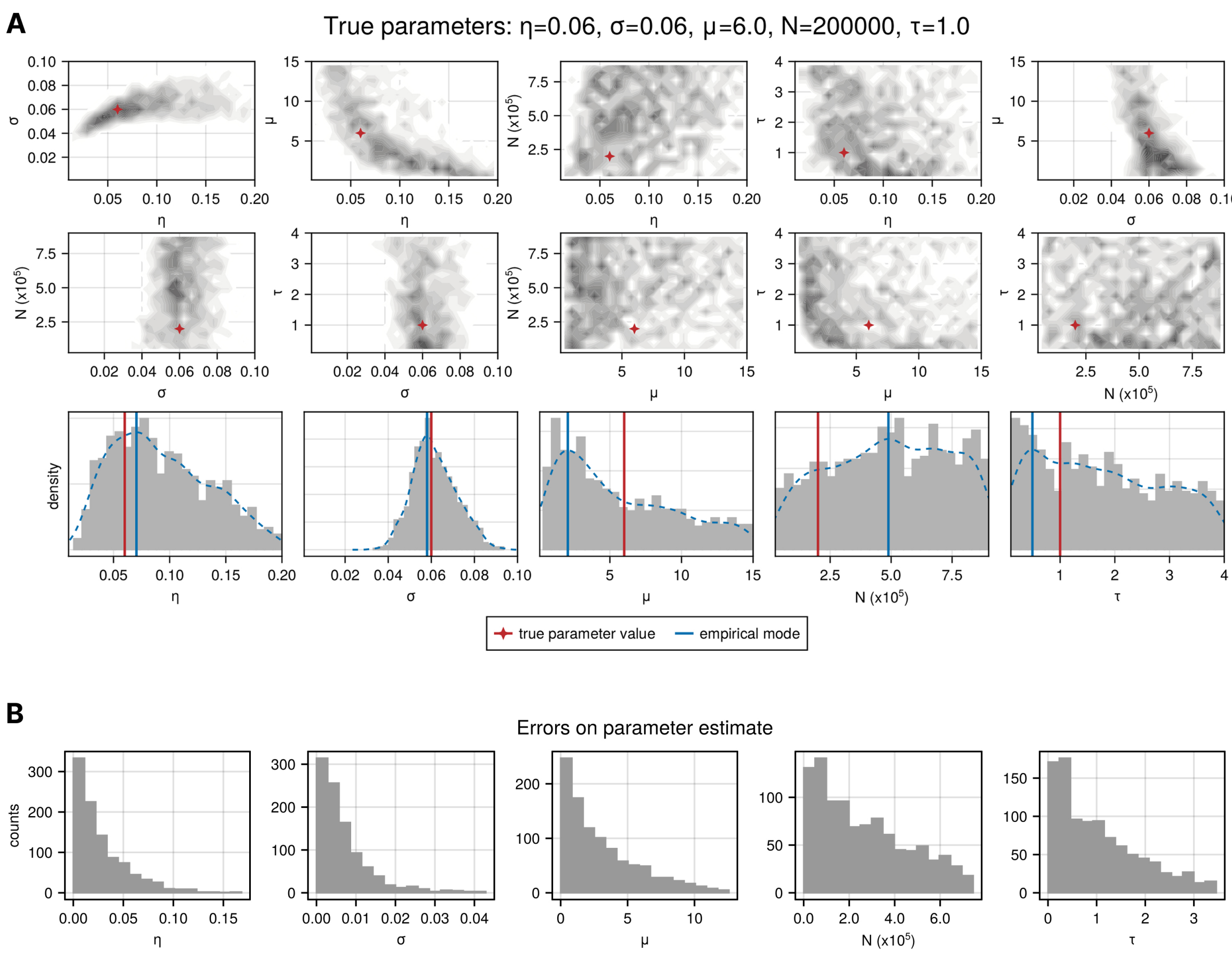

Figure S7. Validation of approximate Bayesian computation on variant trajectories from simulated datasets. A) Posterior distributions of evolutionary parameters for a single simulated dataset with known parameter values. Uniform prior distributions: $\eta \in [0.01, 0.4]$, $\sigma \in [0.001, 0.1]$, $\mu \in [0.1, 30]$, $\tau \in [0.1, 10]$ and $N \in [10^5, 9 \times 10^5]$. Top rows: 2D projections of the full 5-parameter posterior distribution for parameter pairs. Bottom row: marginal distributions for each parameter, with estimated value (mode of the empirical distribution) and true value indicated as red and blue vertical lines respectively. **B)** Distribution of errors on estimated parameters from ABC for 1000 simulated datasets.

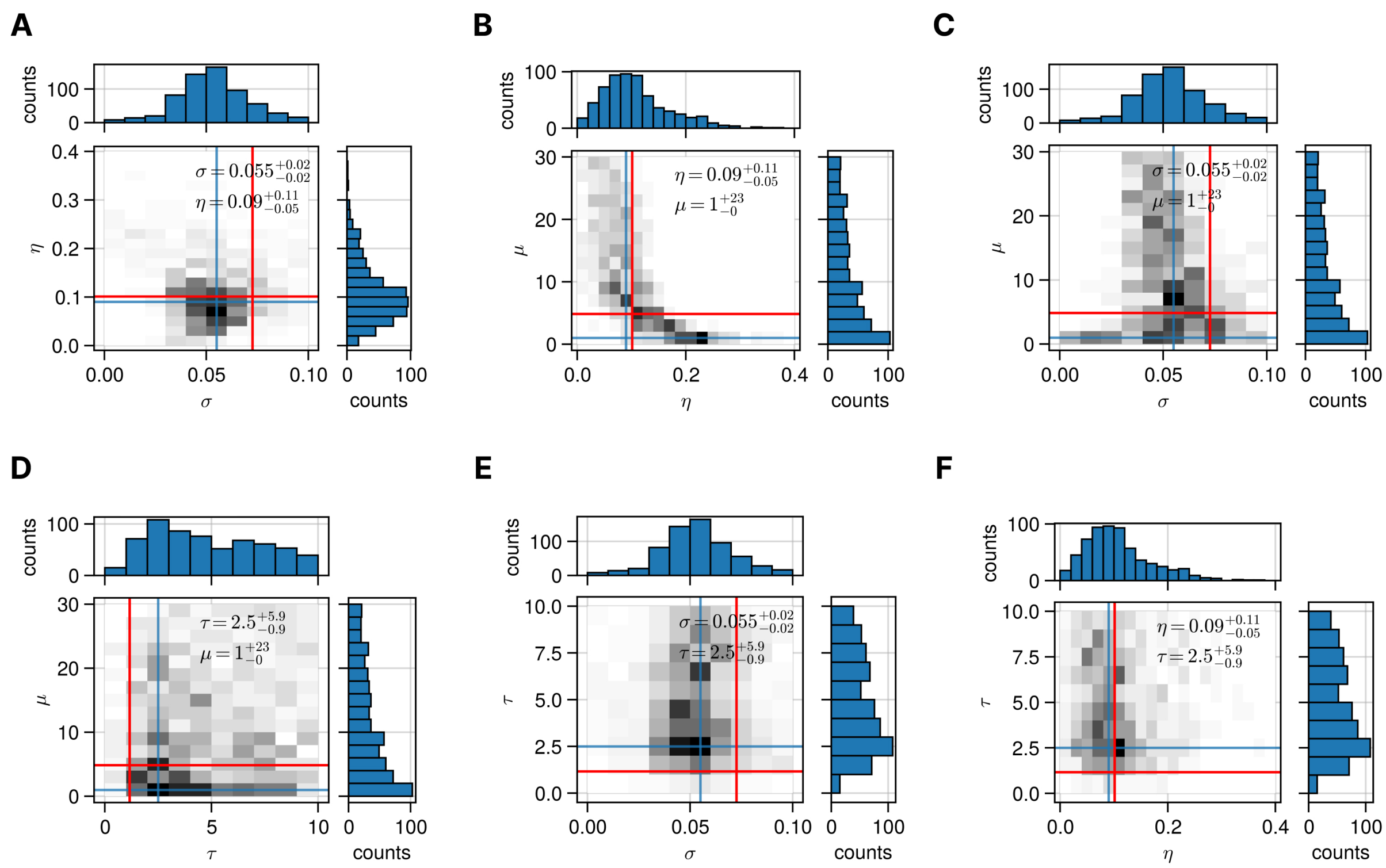

Figure S8. Validation of approximate Bayesian computation on SFS and number of clones on simulated data. A-F) A set of parameter values (red) have been used to generate a single realization of the HSC dynamics, which is considered as ground truth and used to test the accuracy of the inferential framework. 65,000 virtual patients were simulated with parameters randomly drawn from uniform priors, with only the 1% most similar retained in the posterior distribution. Uniform prior distributions: $\eta \in [0.01, 0.4]$, $\sigma \in [0.001, 0.1]$, $\mu \in [0.1, 30]$ and $\tau \in [0.1, 10]$. We fixed $N = 10^5$ for this inference. The inferred point estimates (modes) are shown in blue. The target values (red) are: $\eta = 0.10, \sigma = 0.072, \mu = 4.86, \tau = 1.16$.

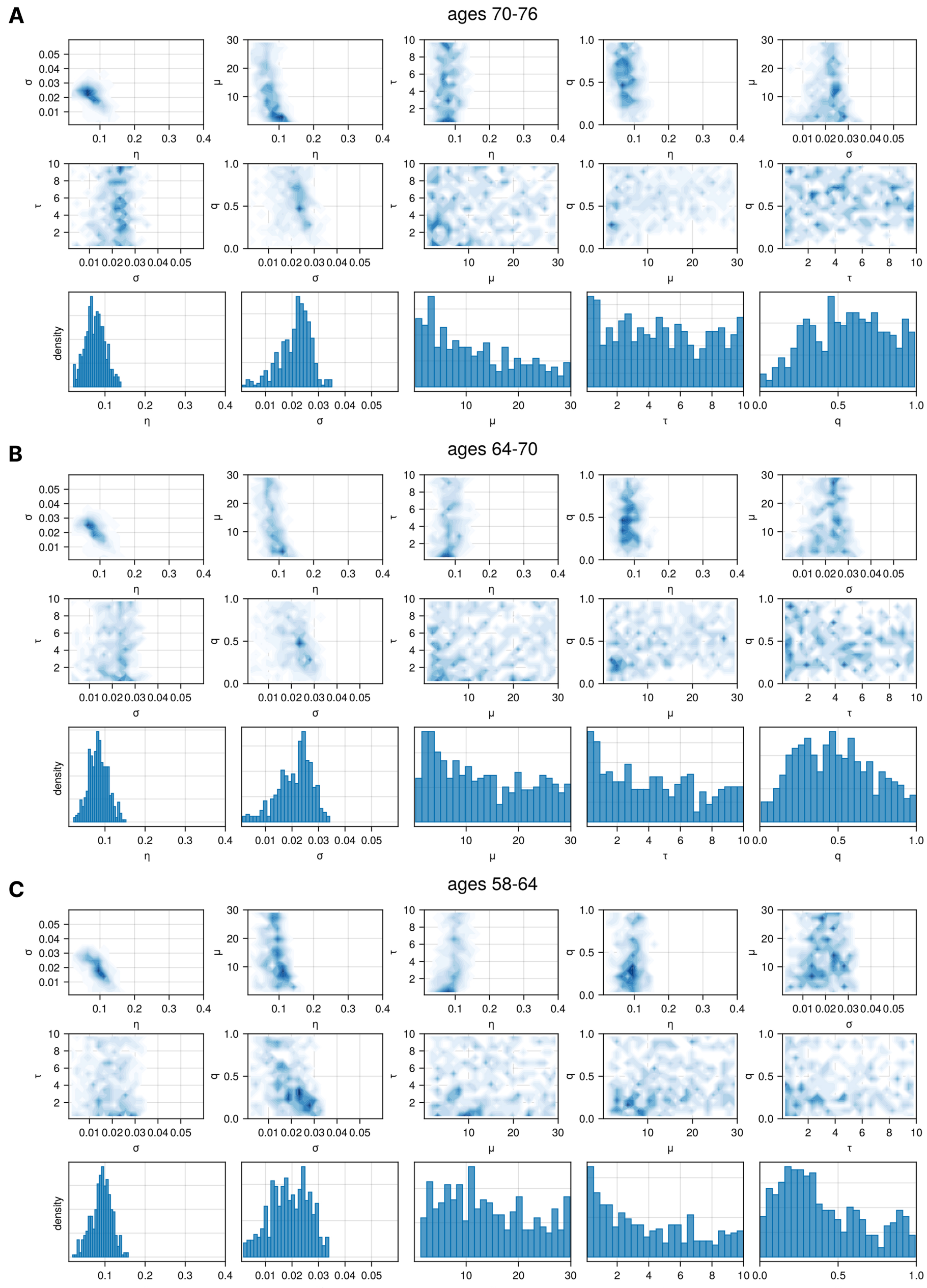

Figure S9. Parameter posterior distributions obtained via approximate Bayesian computation on time-resolved clone trajectories from Fabre et al. donor data. A-C) For each age group 40,000 virtual patients were simulated with parameters randomly drawn from uniform priors, with only the 1% most similar retained in the posterior. Uniform prior distributions: $\eta \in [0.01, 0.4]$, $\sigma \in [0.001, 0.1]$, $\mu \in [0.1, 30]$ and $\tau \in [0.1, 10]$. We fixed $N = 10^5$ for this inference. Top rows: 2D projections of the full 5-parameter posterior distribution for parameter pairs. Bottom row: marginal distributions for each parameter.

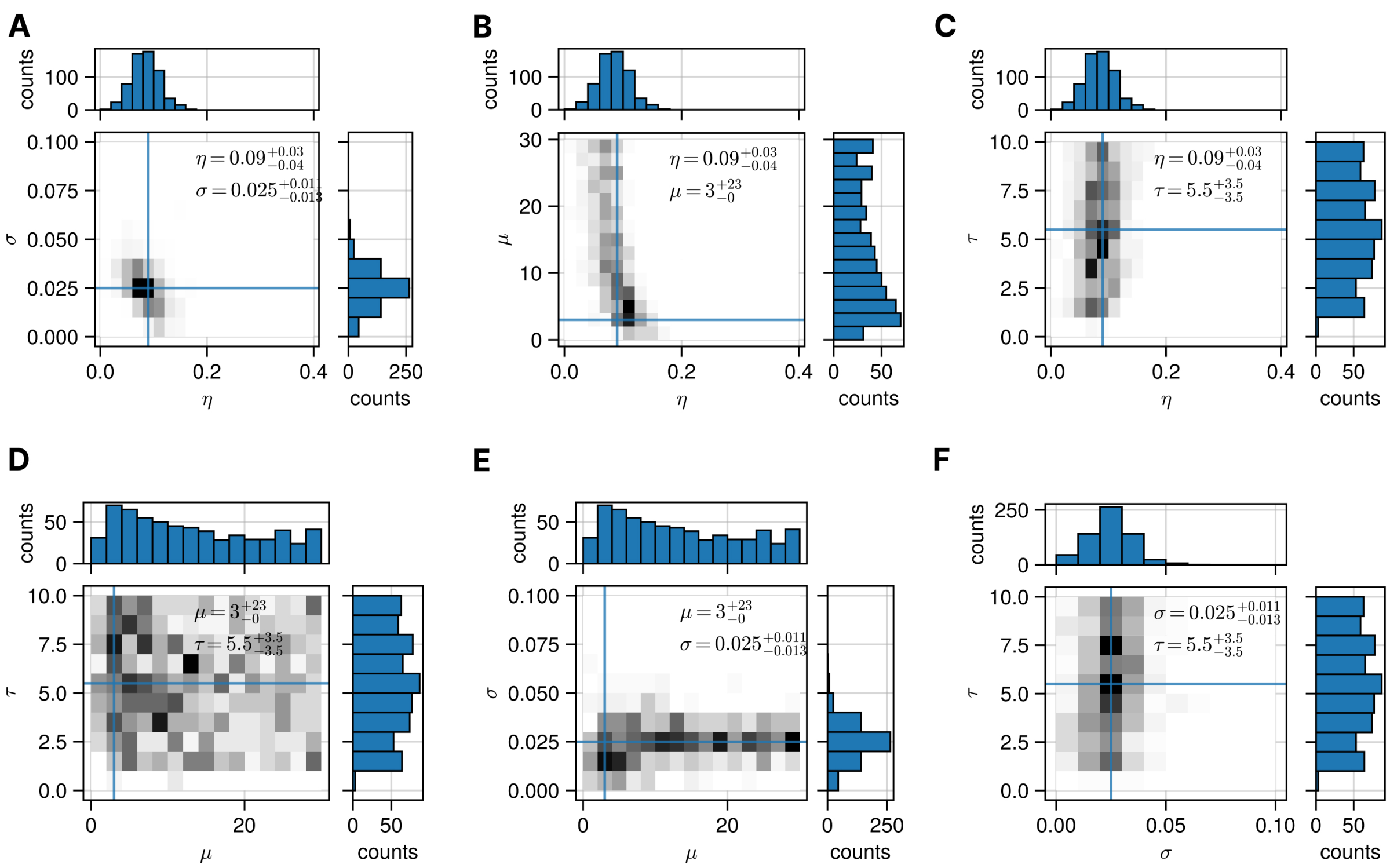

Figure S10. Parameter posterior distributions obtained via approximate Bayesian computation on the SFS and the number of detectable expanded clones from Mitchell et al. data. A-F) 65,000 virtual patients were simulated with parameters randomly drawn from uniform priors, with only the 1% most similar retained in the posterior. Uniform prior distributions: $\eta \in [0.01, 0.4]$, $\sigma \in [0.001, 0.1]$, $\mu \in [0.1, 30]$ and $\tau \in [0.1, 10]$. We fixed $N = 10^5$ for this inference. For pairs of parameters 2D projections of the posterior are shown alongside individual marginal distributions and their modes (point estimates).

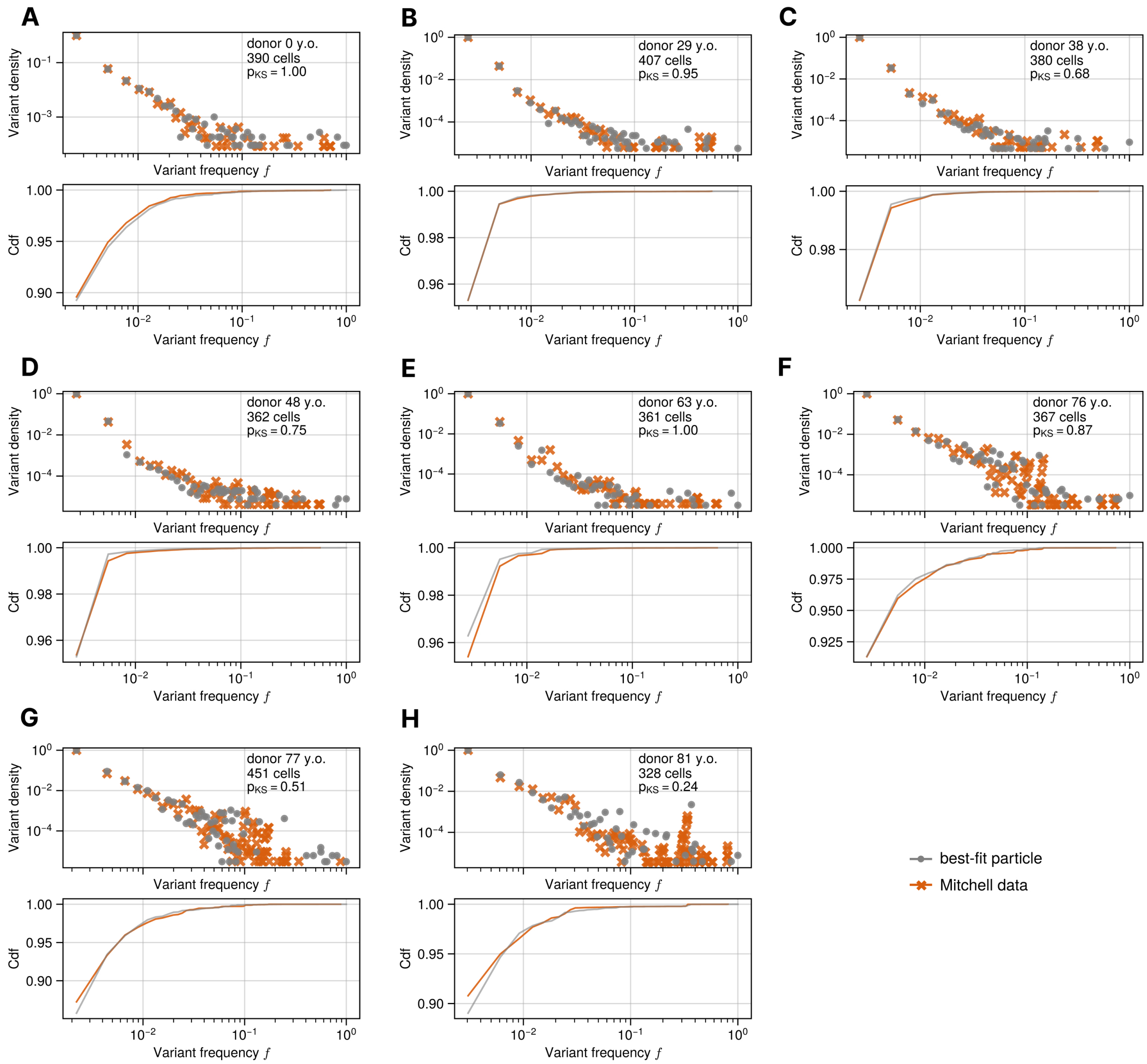

Figure S11. The SFS of the simulations closest to the data according to the SFS and the number of expanded clones summary statistics. A-H) The SFS (top) and the SFS cumulative distributions (bottom) computed from 216-451 whole genome sequenced single cells (orange crosses) and from the realizations of the agent-based simulation that fit the data best (gray dots) for different ages. The best fits recapitulate the stochastic behavior of clonal expansions, detected in the SFS as peaks in the higher frequency domain.

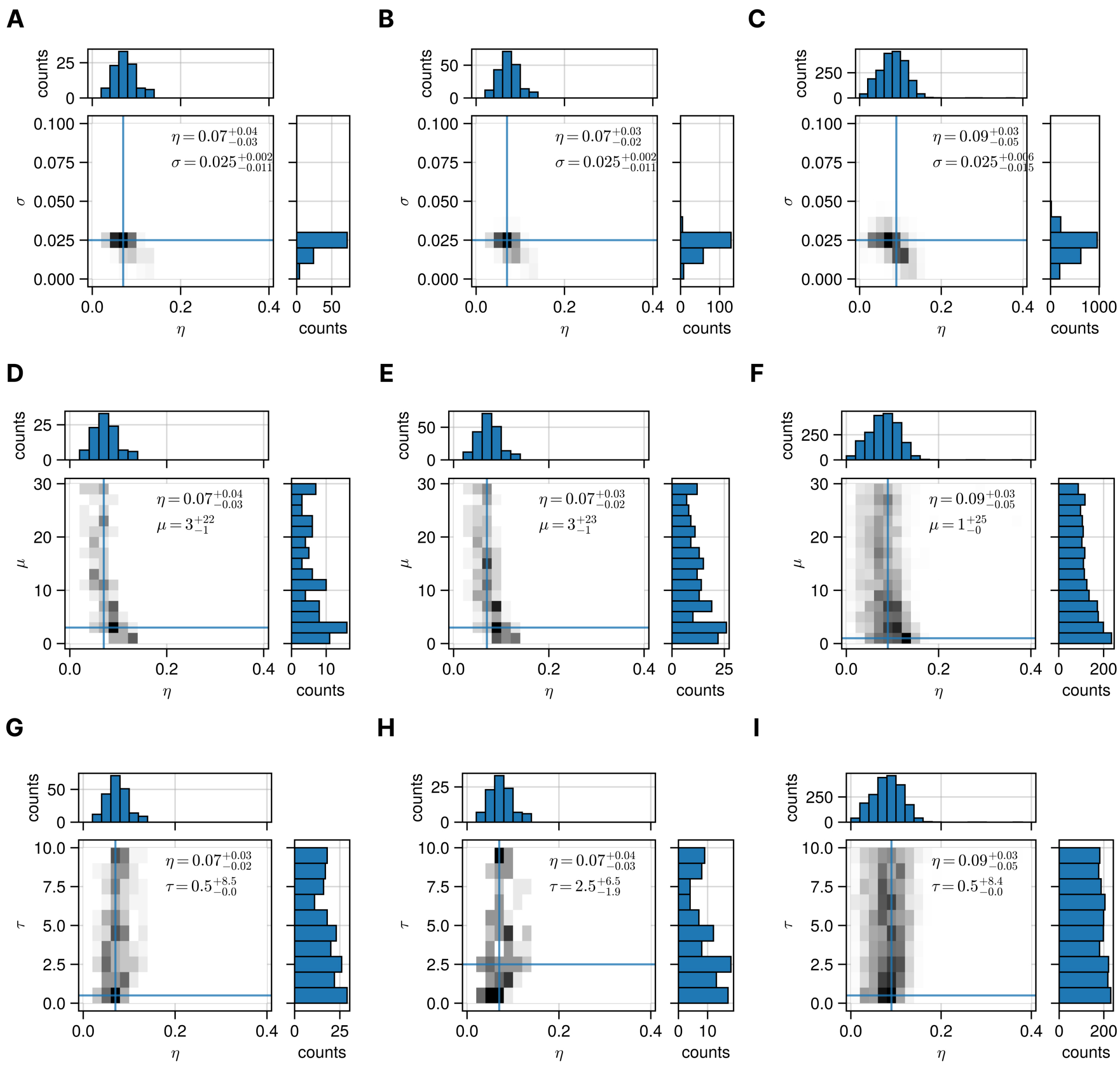

Figure S12. Sensitivity analysis for the posterior distributions inferred from the time-resolved clone trajectories from Fabre et al. data. The posterior distributions and their modes show consistent parameter values inferred for (η, σ, μ) from different percentages of the total 40,000 realizations of the HSC dynamics. Uniform prior distributions: $\eta \in [0.01, 0.4]$, $\sigma \in [0.001, 0.1]$, $\mu \in [0.1, 30]$, $\tau \in [0.1, 10]$. We fixed $N = 10^5$ for this inference. This suggests that the point values inferred are not influenced by the number of samples retained. **A-C)** The 2D posterior distributions of the pair η-σ considering 0.25%, 0.5% and 5% of the runs along with the marginal posterior distributions. **D-F)** The 2D posterior distributions of the pair η-μ considering 0.25%, 0.5% and 5% of the runs along with the marginal posterior distributions. **G-I)** The 2D posterior distributions of the pair η-τ considering 0.25%, 0.5% and 5% of the runs along with the marginal posterior distributions.

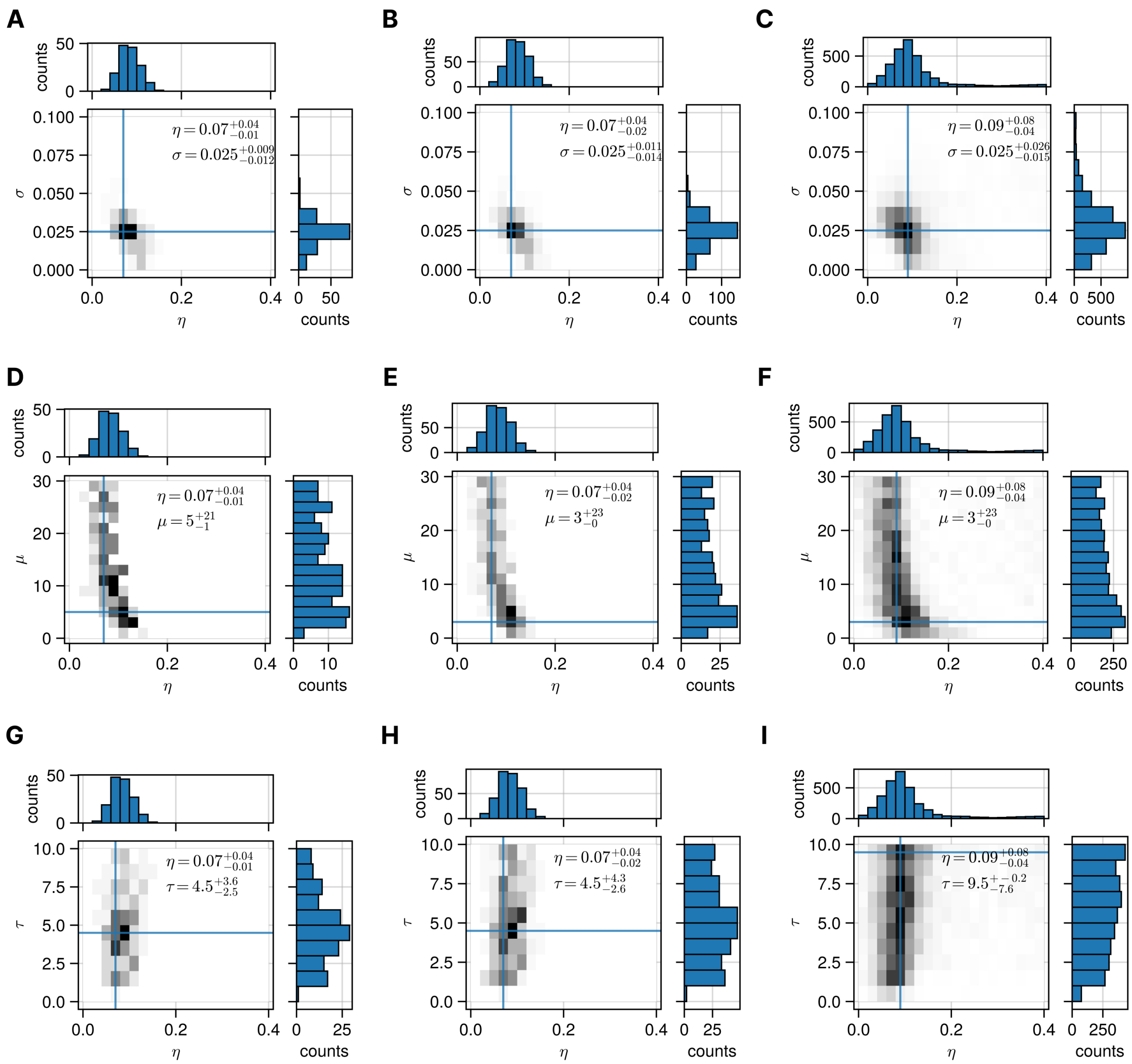

Figure S13. Sensitivity analysis for the posterior distributions inferred from the SFS and the number of detectable expanded clones from Mitchell et al. data. The posterior distributions and their modes show consistent parameter values inferred for (η, σ, μ) from different percentages of the total 65,000 realizations of the HSC dynamics. This suggests that the point values inferred are not influenced by the number of samples retained. Uniform prior distributions: $\eta \in [0.01, 0.4]$, $\sigma \in [0.001, 0.1]$, $\mu \in [0.1, 30]$ and $\tau \in [0.1, 10]$. We fixed $N = 10^5$ for these inferences. **A-C)** The 2D posterior distributions of the pair η-σ considering 0.25%, 0.5% and 5% of the runs along with the marginal posterior distributions. **D-F)** The 2D posterior distributions of the pair η-μ considering 0.25%, 0.5% and 5% of the runs along with the marginal posterior distributions. **G-I)** The 2D posterior distributions of the pair η-τ considering 0.25%, 0.5% and 5% of the runs along with the marginal posterior distributions.

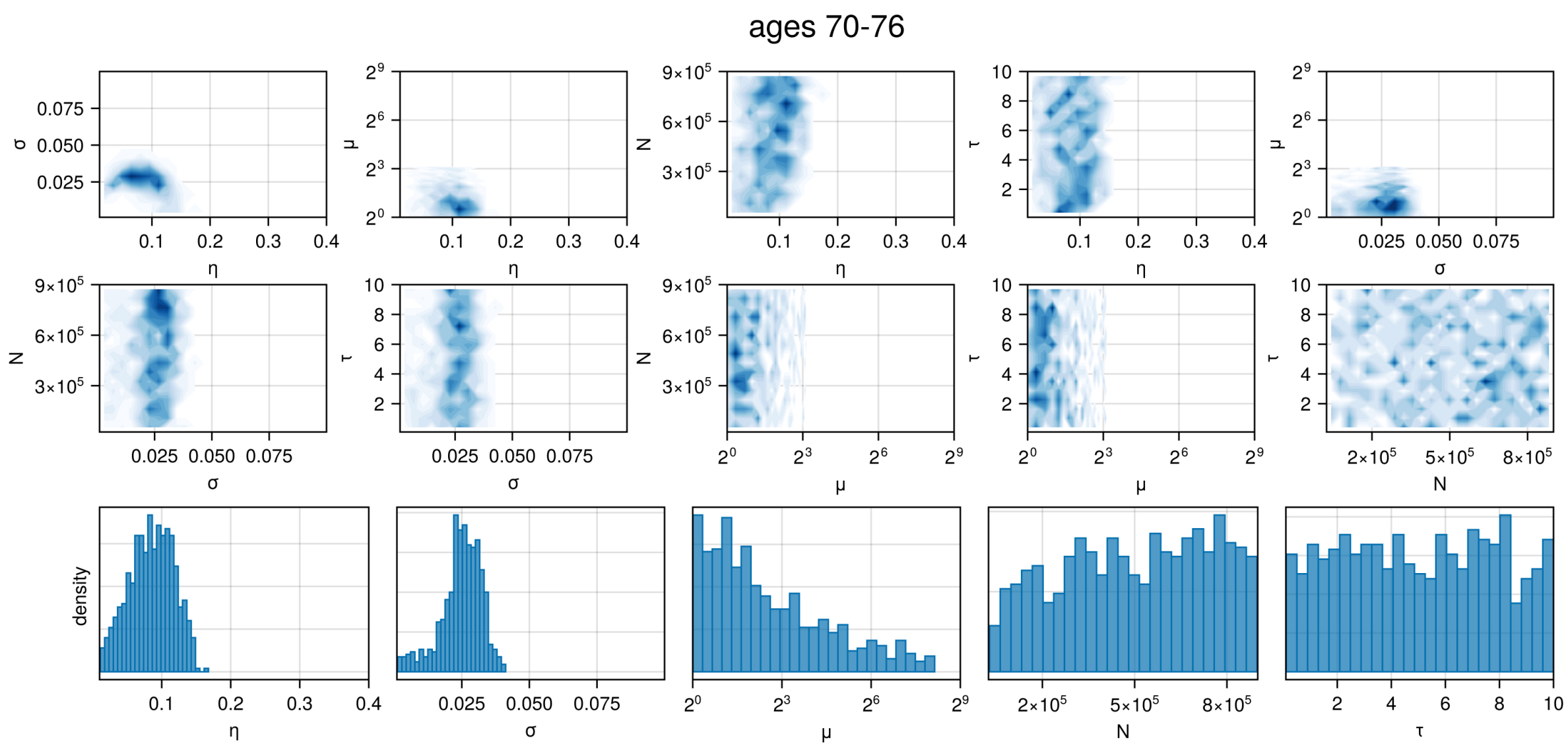

Figure S14. Parameter posterior distributions obtained via approximate Bayesian computation on time-resolved clone trajectories from Fabre et al. donor data with extended prior for μ. 40,000 virtual patients were simulated with parameters randomly drawn from uniform priors, with only the 1% most similar retained in the posterior. Uniform prior distributions: $\eta \in [0.01, 0.4]$, $\sigma \in [0.001, 0.1]$, $\mu \in [1, 512]$, $\tau \in [0.1, 10]$ and $N \in [10^5, 9 \times 10^5]$. Top rows: 2D projections of the full 5-parameter posterior distribution for parameter pairs. Bottom row: marginal distributions for each parameter.

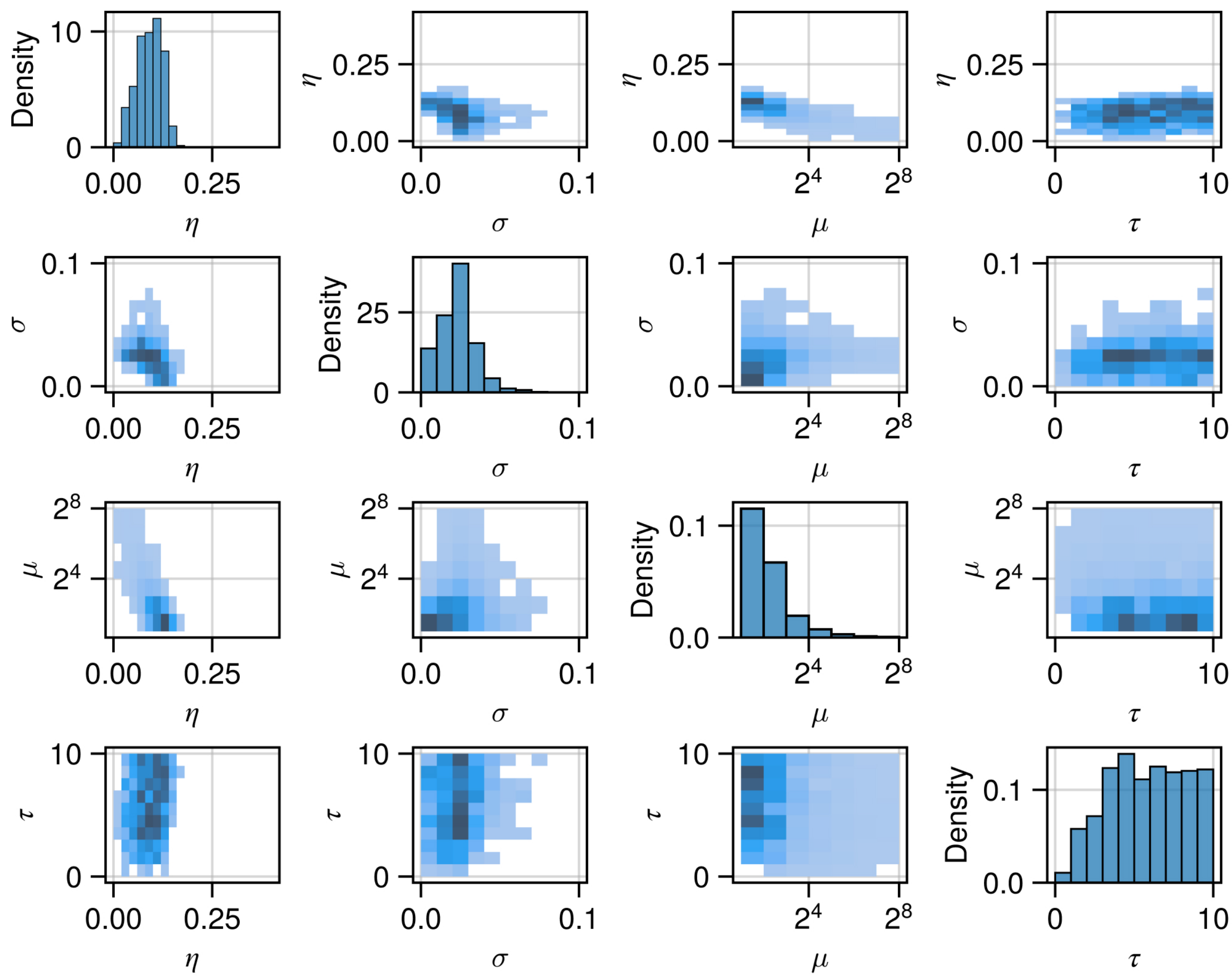

Figure S15. Parameter posterior distributions obtained via approximate Bayesian computation on the SFS and the number of detectable expanded clones from Mitchell et al. data using a wider prior distribution for the arrival rate μ. 65,000 virtual patients were simulated with parameters randomly drawn from uniform priors, with only the 1% most similar retained in the posterior. A wider uniform prior distribution for the arrival rate μ was used for this inference: $\mu \in \{1, 2, 4, 8, 64, 256\}$. The other uniform prior distributions: $\eta \in [0.01, 0.4]$, $\sigma \in [0.001, 0.1]$ and $\tau \in [0.1, 10]$. We fixed $N = 10^5$ for this inference.

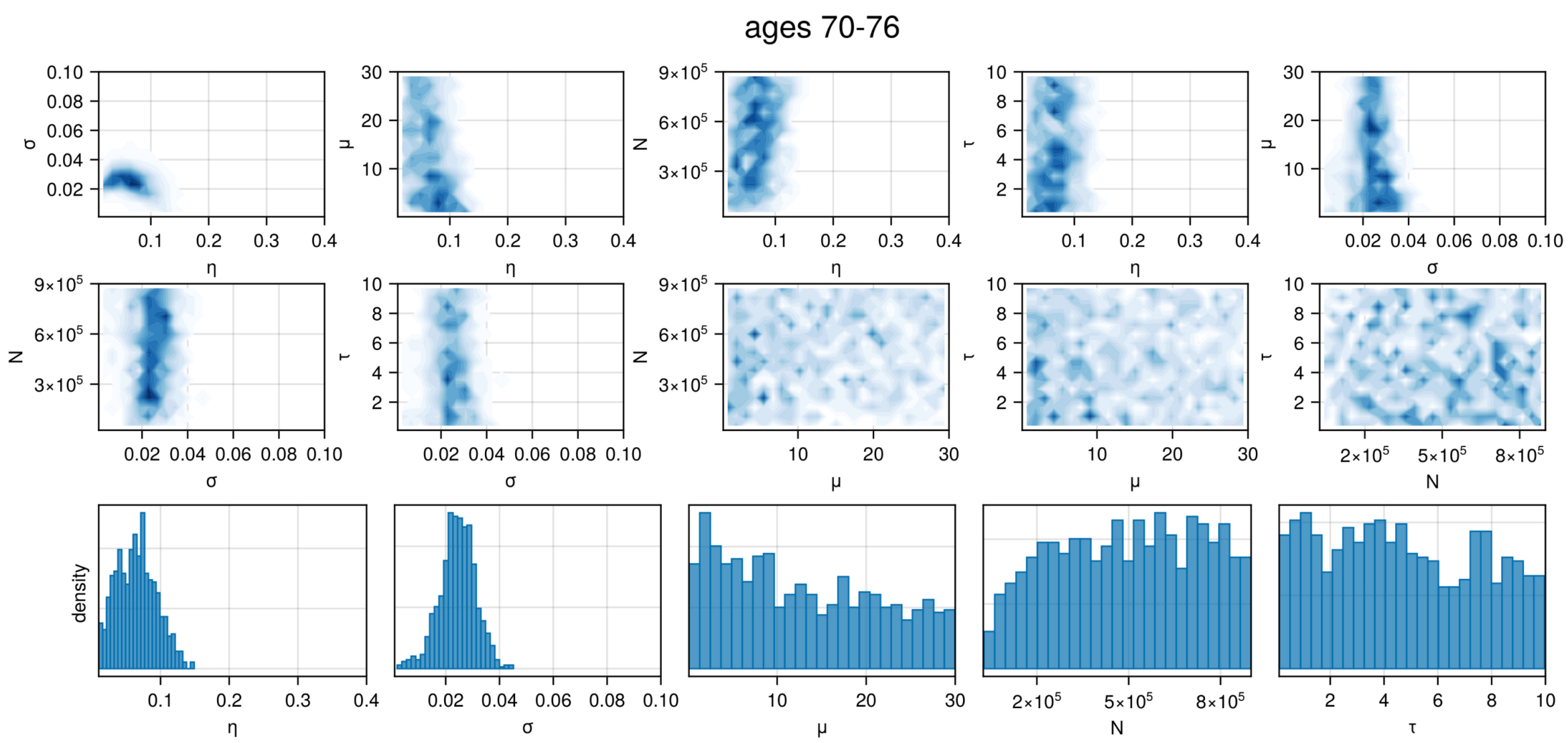

Figure S16. Parameter posterior distributions including the total HSC population size obtained via approximate Bayesian computation on time-resolved clone trajectories from Fabre et al. donor data. 40,000 virtual patients were simulated with parameters randomly drawn from uniform priors, with only the 1% most similar retained in the posterior. Uniform prior distributions: $\eta \in [0.01, 0.4]$, $\sigma \in [0.001, 0.1]$, $\mu \in [0.1, 30]$, $\tau \in [0.1, 10]$ and $N \in [10^5, 9 \times 10^5]$. Top rows: 2D projections of the full 5-parameter posterior distribution for parameter pairs. Bottom row: marginal distributions for each parameter.

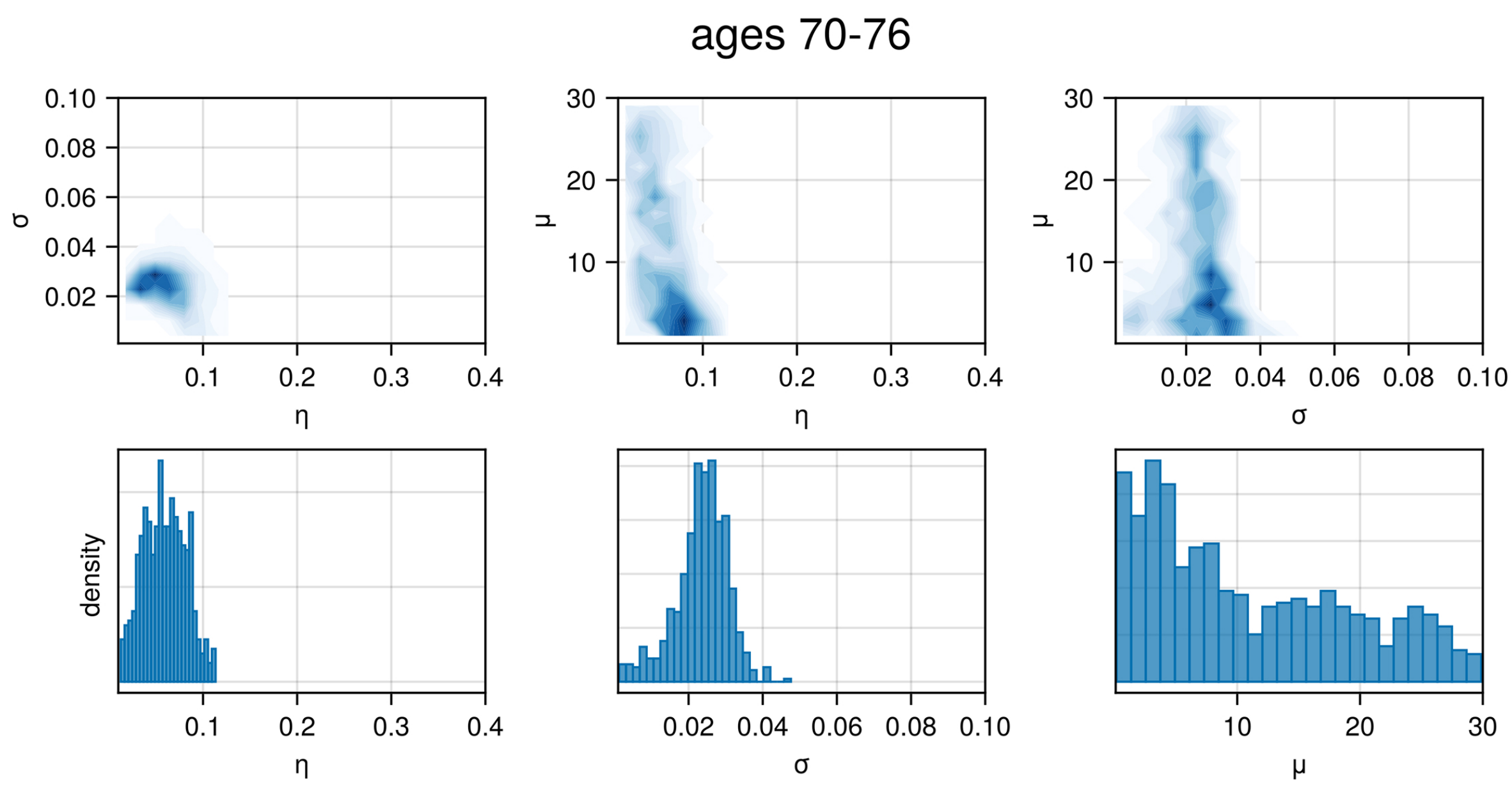

Figure S17. Parameter posterior distributions obtained via approximate Bayesian computation on time-resolved clone trajectories from Fabre et al. donor data, assuming known values of N and τ. 40,000 virtual patients were simulated with parameters randomly drawn from uniform priors, with only the 1% most similar retained in the posterior. Uniform prior distributions: $\eta \in [0.01, 0.4]$, $\sigma \in [0.001, 0.1]$, $\mu \in [0.1, 30]$, $\tau = 1$, and $N = 100,000$. Top rows: 2D projections of the full 3-parameter posterior distribution for parameter pairs. Bottom row: marginal distributions for each parameter.

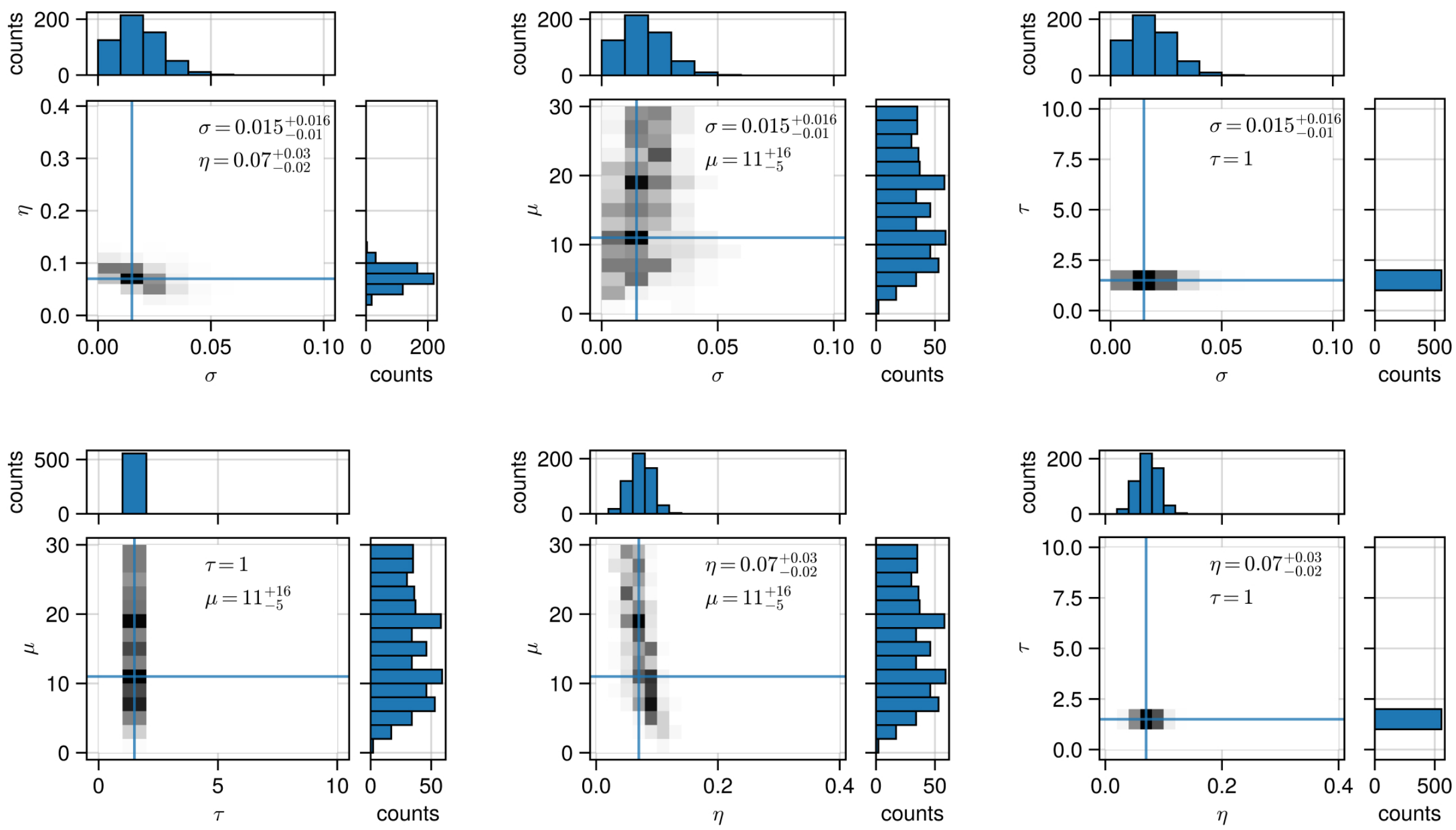

Figure S18. Posterior distributions obtained via approximate Bayesian computation on the SFS and the number of detectable expanded clones from Mitchell et al. data using fixed values for N and τ. 65,000 virtual patients were simulated with parameters randomly drawn from uniform priors, with only the 1% most similar retained in the posterior distribution. We fixed both $\tau = 1$ and $N = 10^5$ for this inference. The uniform prior distributions: $\eta \in [0.01, 0.4]$, $\sigma \in [0.001, 0.1]$ and $\mu \in [0.1, 30]$. The inferred point estimates (modes) are shown in blue.

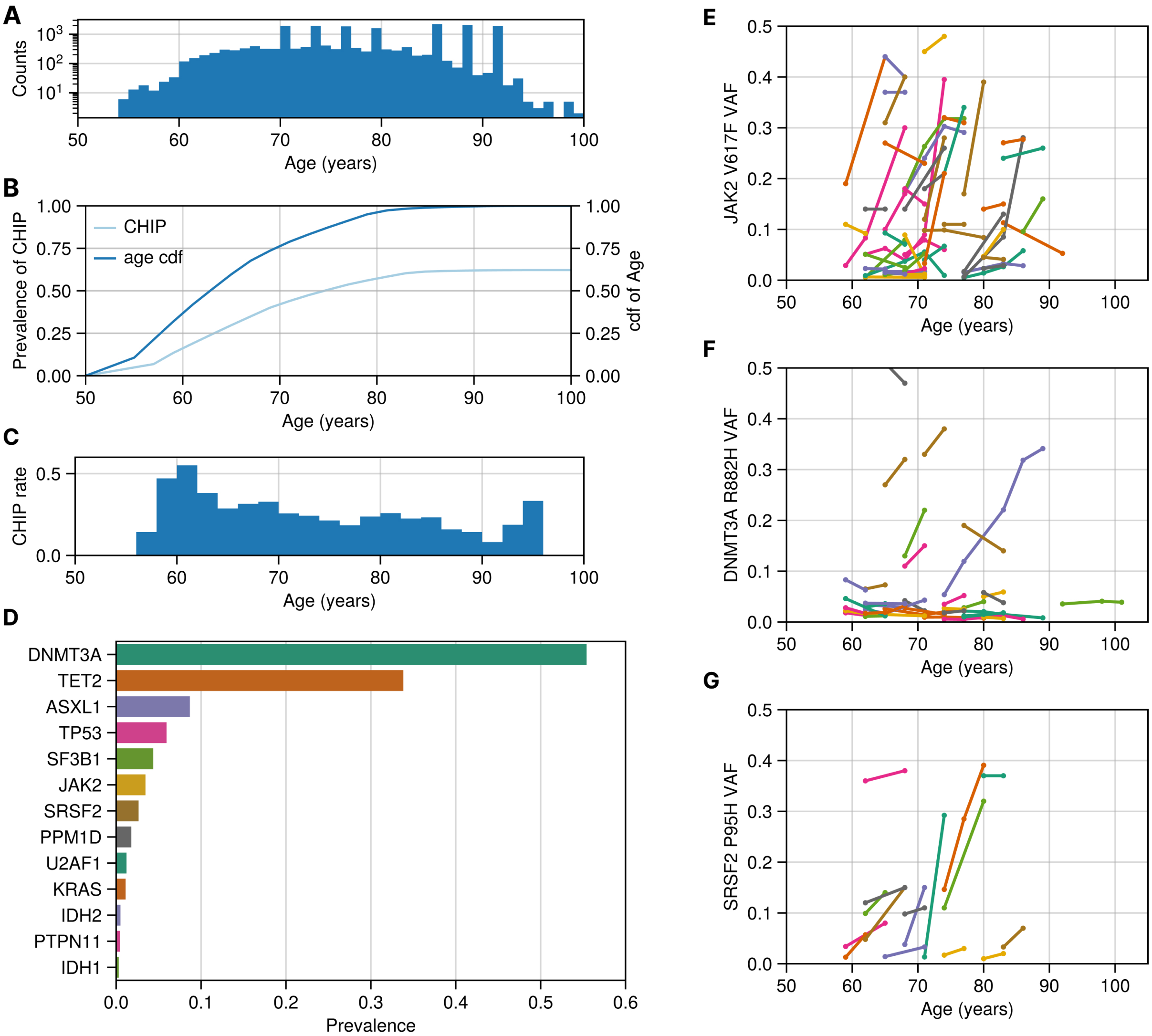

Figure S19. Aggregating three target sequencing datasets to study haematopoiesis in 1,942 individuals. A. The distribution of mutations per age. **B.** The prevalence of clonal haematopoiesis of indeterminate potential (CHIP) shown in light blue and computed as the cumulative sum of the prevalence of donors with at least one variant with VAF$\geq$2%. The cumulative distribution function (cdf) of the age of the donors (dark blue). **C.** The rate of CHIP computed by normalising the number of donors with CHIP within two-year age bins by the number of donors in each bin. **D.** Frequently mutated genes in this cohort. **E-G.** The dynamics of the VAF of three variants over time per different donors (each line represent an individual). *JAK2*-V617F was the most prevalent variant and was found in 67 donors, followed by *DNMT3A*-R882H which was found in 49 donors. *SRSF2*-P95H was found in 26 donors.

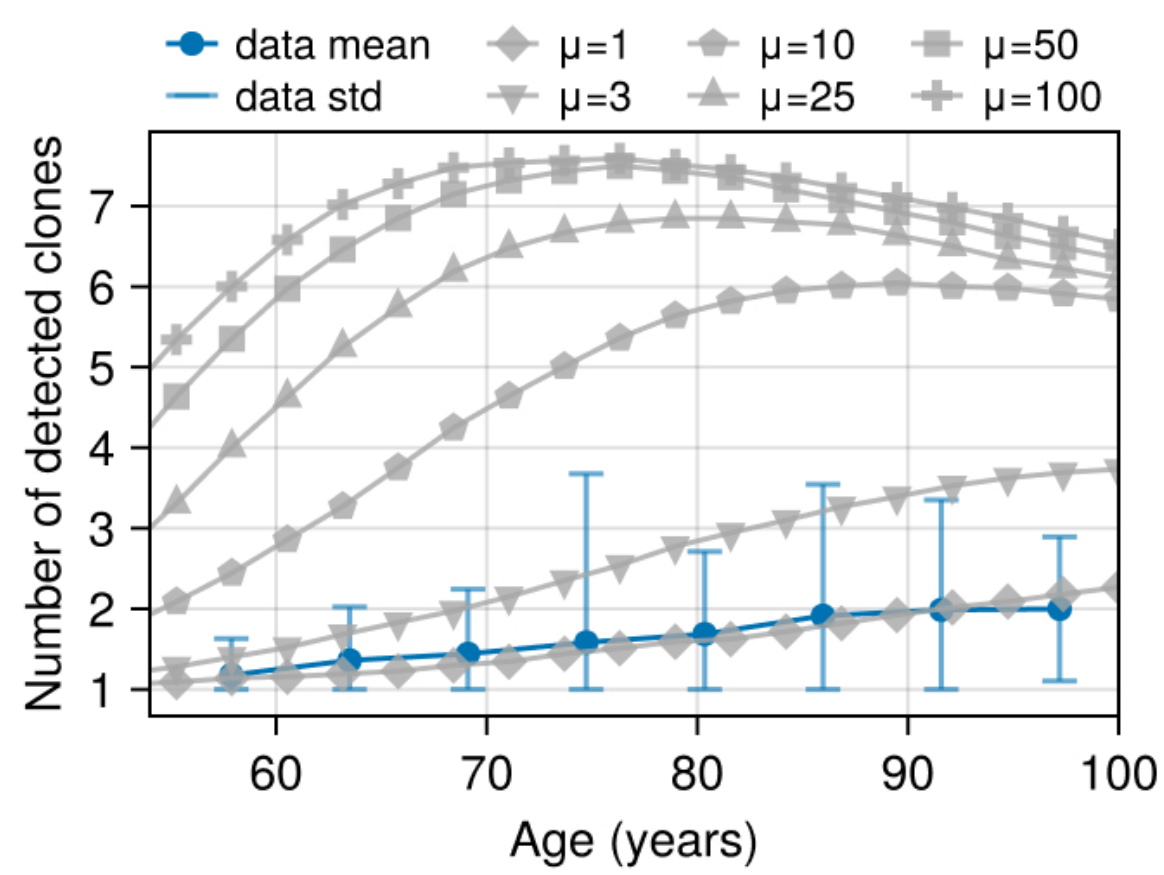

Figure S20. The mean number of detectable clones with age measured in simulations of the SDE model for various values of the clone arrival rate μ (gray lines) alongside the number observed in data (blue lines).

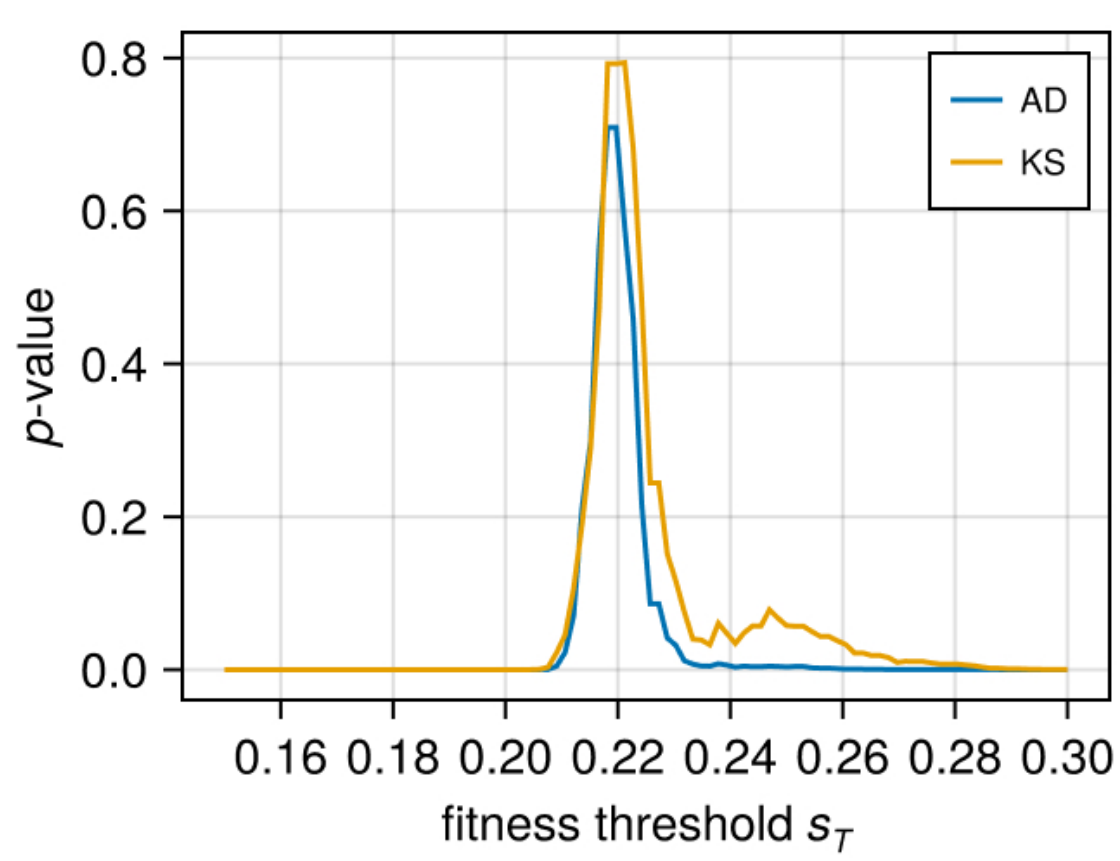

Figure S21. p-values for the Anderson-Darling and Kolmogorov-Smirnov tests when comparing the arrival-time distributions of high-fitness ($s > s_T$) trajectories observed in the data and simulated multi-step clones.

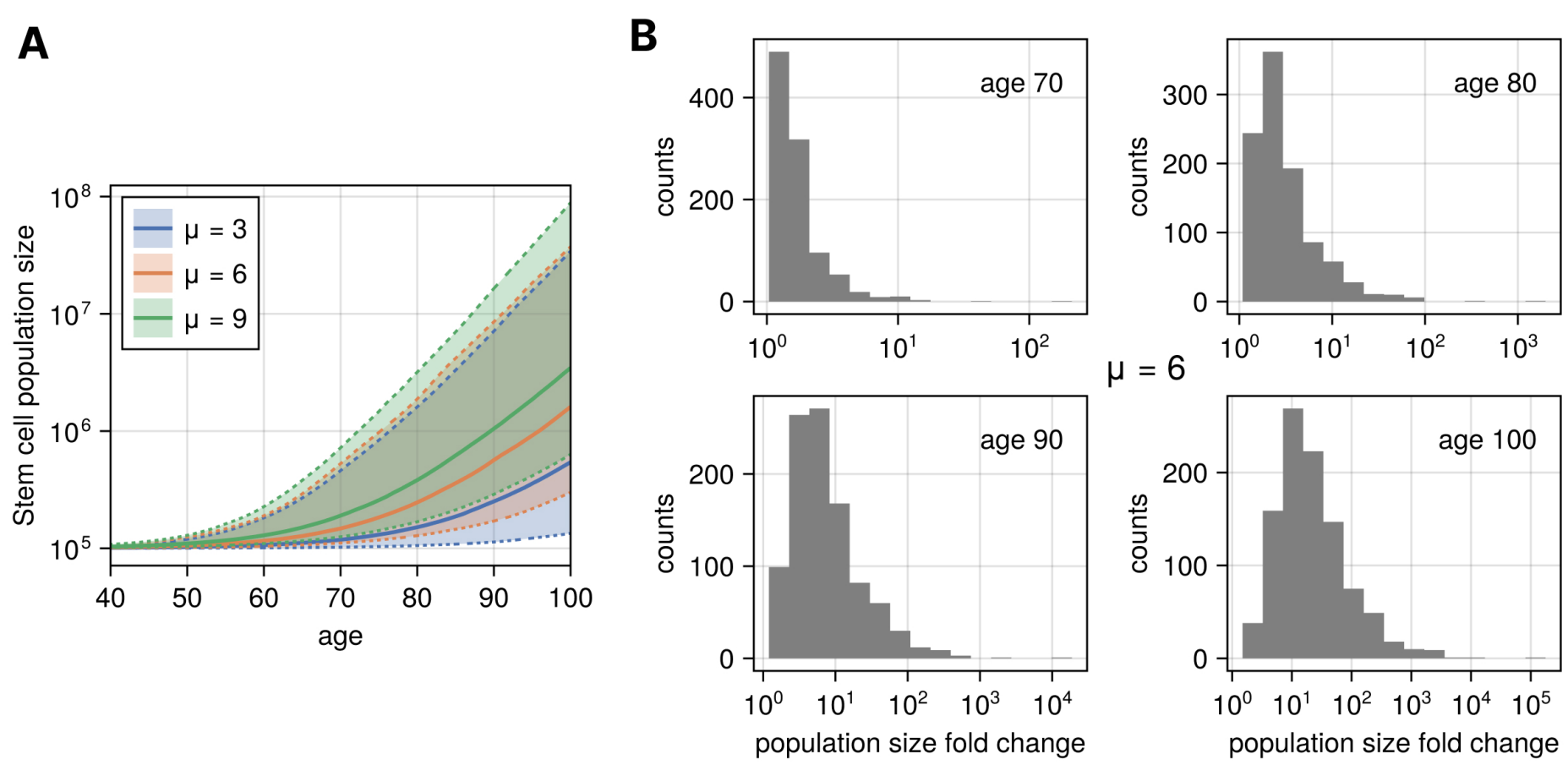

Figure S22. Unconstrained growth of clones leads to exponentially increasing stem cell populations. Under parameter values $\eta = 0.08$, $\sigma = 0.025$, $\tau = 1$, and $N = 10^5$, the HSC population increases many-fold from ages 70 to 100. **A** The median (solid lines) and 95% interval (shaded area) of the HSC population size across 1000 simulations for different values of the accumulation rate of fit clones μ. **B** The distribution of HSC population size fold changes for ages 70, 80, 90, and 100, obtained from 1000 simulations with $\mu = 6$.
