# Supplementary material for: The Evolution of Polyclonal Competition in Aging Hematopoiesis
Source: Cancer Discov. 2026 May 5;16(9):1765–79. doi: 10.1158/2159-8290.CD-25-0990 (PMC13531000; doi:10.1158/2159-8290.CD-25-0990)
Supplement: Supplementary file 2 [file cd-25-0990_supplementary_methods_1_suppsm1.pdf]

Supplementary Methods

Contents

1 Maximum likelihood estimation of logistic trajectories **1**

2 Clonal competition as a multi-type capacity-limited birth-death process **2**
- 2.1 The SDE system for clonal competition . . . 3
  - 2.1.1 Deriving the SDE system . . . 3
  - 2.1.2 Numerical simulations of the SDE system . . . 4
- 2.2 Unconstrained growth modeling . . . 5

3 Bayesian inference on clonal trajectories to estimate parameter values **6**
- 3.1 Simulation of a single ABC particle . . . 6
- 3.2 Prior distribution ranges . . . 6
- 3.3 Testing the ABC convergence with simulated data . . . 7

4 Accumulation of neutral mutations in the multi-type birth-death process **7**
- 4.1 Ontogenic growth of the HSC population . . . 8
- 4.2 Agent-based model with inclusion of neutral mutations . . . 8

5 The single-cell HSC dynamics **9**
- 5.1 The dataset of whole genome sequencing of HSC colonies . . . 9
- 5.2 The HSC site frequency spectrum dynamics . . . 9
- 5.3 Model parametrisation with the mutational burden . . . 10
- 5.4 Bayesian inference on site frequency spectra to estimate parameter values . . . 11
  - 5.4.1 Summary statistics . . . 11
  - 5.4.2 Longitudinal inference using the SFS and the number of clones . . . 11
  - 5.4.3 Testing the Bayesian framework on simulated data . . . 12

6 Combining three longitudinal sequencing datasets **12**
- 6.1 The rate of CH in data . . . 12
- 6.2 The number of detectable clones in data and simulations . . . 13

7 Bayesian inference of innate fitness and arrival time on individual trajectories **14**
- 7.1 Estimation error arises from inherent stochasticity . . . 14
- 7.2 Genic variant fitness estimates from multiple donors . . . 15

8 The arrival time distribution of detectable variants **16**
- 8.1 Single-event clones . . . 16
- 8.2 Multiple-event clones . . . 17
- 8.3 Comparing data and simulations . . . 17

1 Maximum likelihood estimation of logistic trajectories

The logistic function — obtained as the solution to the differential equation

$$\mathrm{d}x(t)/\mathrm{d}t = r_{\log}\, x(t)[1 - x(t)/K] \tag{SE1}$$

describes the expected growth curve of a mutant under selection in a constant-size population with fixed background fitness [1]. Here, $x(t)$ describes the number of stem cells carrying a certain variant at time and K represents the maximum total number of stem cells (carrying capacity) reachable. The growth rate $r_{\log}$ defines the relative fitness of a variant compared to the background population: $r_{\log} > 0$ (and $r_{\log} < 0$) implies a positively (or negatively) selected variant as the number of cells $x(t)$ increase (or decrease) over time. This equation can be solved analytically and the clone size is given by

$$x(t) = \frac{K}{1 + \frac{K - x_0}{x_0} e^{-r_{\log}(t - t_0)}}. \tag{SE2}$$

As the observed CH variant trajectories in human blood are sigmoidal, the above equation can be used to approximate a trajectory in a short time window. If a trajectory is well-described by Equation (SE2), the rate parameter $r_{\log}$ quantifies the selection strength acting on the clone in the observed time window.

We fit all variant size trajectories reported by Fabre et al. [2] to Equation (SE2) using a maximum likelihood estimation as follows. We assumed each measurement of a variant allele's frequency $\tilde{x}_i$ to be a binomial sampling, where the success probability is given by the (unknown) true frequency x_i and the number of trials is given by the coverage V of that site. Thus for any proposed trajectory $y(t|\beta)$ (where β is the set of parameters to fit), we could compute the probability of obtaining the observed sequence of measurements $\tilde{X}_i = \tilde{x}_i V$ at times t_i as

$$\mathcal{L}(\beta) = \prod_i \mathbb{P}\{\tilde{X}_i \,|\, y(t_i|\beta), V\} \tag{SE3}$$

where

$$\mathbb{P}\{\tilde{X}_i \,|\, V, y(t_i|\beta)\} = \binom{V}{\tilde{X}_i} y(t_i|\beta)^{\tilde{X}_i} [1 - y(t_i|\beta)]^{V - \tilde{X}_i} \tag{SE4}$$

which is the binomial probability of sampling successfully $\tilde{X}_i$ times out of V trials with success probability $y(t_i|\beta)$. Equation (SE3) is known as the likelihood function, and is maximized with respect to β to obtain the best fit. For each trajectory we maximized three different likelihood functions: one assuming the underlying trajectory is increasing, another assuming it is decreasing, and finally one assuming it is constant ($y(t) = x_0$). In the case of an increasing trajectory on a non-sex chromosome we took $K = 0.5$ and $x_0 = 1/2N$ as fixed parameters and — assuming $r_{\log} > 0$ — maximized with respect to $\beta = \{t_0, r_{\log}\}$. For a decreasing trajectory we took $\beta = \{t_0, x_0, r_{\log}\}$ (x_0 is then the size at which the clone became negatively selected) and instead assumed $r_{\log} < 0$. Finally for the saturating trajectory $r_{\log} = 0$ and the single free parameter $\beta = \{x_0\}$ (the fixed true clone frequency) was fit. The trajectory with the highest maximum likelihood was then taken as the best fit.

2 Clonal competition as a multi-type capacity-limited birth-death process

We model the haematopoietic stem cell compartment as a collection of cells, each carrying a set of genetic mutations. These cells individually undergo divisions and death as independent Poisson processes in time, where in case of the former the cell is replaced by two daughter cells inheriting the set of mutations from the parent, and in case of the latter the cell is removed from the population. We consider first only fitness-granting mutations which alter the birth rate of a cell. We take these to occur

as a Poisson process in time, and assume each new mutation is distinct from those already existing, a supposition typically referred to as the *infinite sites model*. Denoting the set of all fitness carrying mutations as $\mathcal{I}$, a cell of type $i \in \mathcal{I}$ as $\mathcal{X}_i$, and the birth, death, and mutation rates as β, δ, and μ respectively, the model is summarized through the following reactions:

$$\begin{aligned} &\mathcal{X}_i \xrightarrow{\beta_i} \mathcal{X}_i + \mathcal{X}_i \\ &\mathcal{X}_i \xrightarrow{\delta_i} \varnothing \\ &\mathcal{X}_i \xrightarrow{\mu} \mathcal{X}_j \quad j \notin \mathcal{I}. \end{aligned} \tag{SE5}$$

An important constraint is that the total stem cell population is expected to remain at an approximately constant size throughout most of the human lifespan [3, 4]. We take this to occur by enforcing an equality of the total population-level birth and death rates:

$$\mathcal{B} = \sum_{c \in \text{cells}} \beta_c = \mathcal{D} = \sum_{c \in \text{cells}} \delta_c \tag{SE6}$$

with β_c and δ_c the birth and death rates of cell c. If all cells are of the same wild type — having identical birth and death rates — this is trivially realized by setting $\beta_c = \delta_d = \beta = \delta, \forall c, d$. This amounts to a completely neutral population, where $\mathcal{B} = N\beta = \mathcal{D} = N\delta$ for a population of size N. If there are K types the population-level rates can instead be written as

$$\mathcal{B} = \sum_{k=1}^{K} \beta_k X_k = \mathcal{D} = \sum_{k=1}^{K} \delta_k X_k \tag{SE7}$$

where X_k denotes the number of cells of type k, and all cells of that type share the birth and death rates β_k and δ_k. We define selection acting on a type k as an altered birth rate with respect to the wild type through a fitness parameter s_k, and a fixed death rate across all types $\forall k : \delta_k = \mathcal{D}/N$:

$$\beta_k = \beta + s_k \tag{SE8}$$

$$\delta_k = \beta + \frac{\sum_j s_j X_j}{N} \tag{SE9}$$

2.1 The SDE system for clonal competition

2.1.1 Deriving the SDE system

From the rates (SE8) and (SE9) a master equation can be constructed for the probability distribution of the size of type k, where we use the shorthand notation $P_n^{(k)} = \mathbb{P}\{X_k = n\}$:

$$\begin{aligned} \frac{\mathrm{d}P_n^{(k)}}{\mathrm{d}t} &= \sum_m \left[T_{m\to n}^{(k)} P_m^{(k)} - T_{n\to m}^{(k)} P_n^{(k)} \right] \\ &= T_{n-1,\uparrow}^{(k)} P_{n-1}^{(k)} + T_{n+1,\downarrow}^{(k)} P_{n+1}^{(k)} \\ &\quad - T_{n,\uparrow}^{(k)} P_n^{(k)} - T_{n,\downarrow}^{(k)} P_n^{(k)} \end{aligned} \tag{SE10}$$

This expression contains the transition rates $T_{n,\uparrow\downarrow}^{(k)}$ for the size of clone k to increase or decrease by 1:

$$\begin{cases} T_{n,\uparrow}^{(k)} = n\beta_k \\ T_{n,\downarrow}^{(k)} = n\delta_k \end{cases} \tag{SE11}$$

Assuming that N is large, meaning that there are many possible values of X_k/N between 0 and 1, we construct an analogous stochastic process where the size is given by a continuous variable $x_k \in [0,1] \subset \mathbb{R}$, representing the frequency of clone k in the population. One convenient way to do this (see for example [5]) is by writing the master equation as a Kramers-Moyal expansion. First, noting that the transition rates here describe displacements of distance $\Delta x = 1/N$, i.e. $T^{(k)}_{n,\uparrow\downarrow} \to T^{(k)}_{\pm\Delta x}(x) = T^{(k)}_{\uparrow\downarrow}(x)$, we rewrite (SE10) in terms of a Taylor expansion of the continuous parameter x:

$$\begin{aligned}\frac{\mathrm{d}P^{(k)}(x)}{\mathrm{d}t} &= \left[\sum_{i=0}^{\infty}\frac{(-\Delta x)^i}{i!}\left(\frac{\partial}{\partial x}\right)^i - 1\right]T^{(k)}_{\uparrow}(x)P^{(k)}(x) \\ &\quad + \left[\sum_{i=0}^{\infty}\frac{(\Delta x)^i}{i!}\left(\frac{\partial}{\partial x}\right)^i - 1\right]T^{(k)}_{\downarrow}(x)P^{(k)}(x) \\ &= \sum_{i=1}^{\infty}\left(-\frac{\partial}{\partial x}\right)^i D_i(x)P^{(k)}(x)\end{aligned} \tag{SE12}$$

where the final equality immediately describes the Kramers-Moyal expansion with coefficients

$$D_i(x) = (\Delta x^i/i!)\left[T^{(k)}_{\uparrow}(x) + (-1)^i T^{(k)}_{\downarrow}(x)\right] \tag{SE13}$$

Using our assumption of small steps $\Delta x = 1/N$ we truncate after the second term, to obtain a Fokker-Planck equation with coefficients

$$\begin{aligned} D_1(x) &= x(\beta_k - \delta_k) \\ D_2(x) &= \frac{x}{2N}(\beta_k + \delta_k) \end{aligned} \tag{SE14}$$

These can be used to construct an equivalent stochastic differential equation

$$\mathrm{d}x = \mu(x,t)\,\mathrm{d}t + \sigma(x,t)\,\mathrm{d}W_t \tag{SE15}$$

where in the Itô formulation the drift and diffusion coefficients take the form

$$\mu(x,t) = D_1(x,t) \tag{SE16}$$

$$\sigma(x,t) = \sqrt{2D_2(x,t)} \tag{SE17}$$

Thus, plugging in (SE8) and (SE9), we have

$$\mathrm{d}x_k = s_k x_k\left(1 - \sum_j \frac{s_j}{s_k}x_j\right)\mathrm{d}t + \sqrt{\frac{1}{N}\left(2\beta + s_k + \sum_j s_j x_j\right)x_k}\,\mathrm{d}W_t \tag{SE18}$$

which describes a set of coupled stochastic differential equations for $k = 1, 2, \ldots, K$.

2.1.2 Numerical simulations of the SDE system

With Equation (SE18) describing the time-evolution of existing variants clones, all that remains to obtain the model described by Equation (SE5) is the introduction of newly arriving clones in time.

It suffices to take the total number of equations K to be a Poisson process in time, and have newly introduced clones initiate at size $1/N$:

$$\begin{cases} \mathrm{d}x_k = s_k x_k \left(1 - \sum_l \frac{s_l}{s_k} x_l\right) \mathrm{d}t + \sqrt{\frac{1}{N}\left(2\beta + s_i + \sum_j s_j x_j\right) x_i}\, \mathrm{d}W_t\,, \quad 0 < x_k < 1 \wedge t > t_k \\ \mathrm{d}x_k = 0, \qquad x_k \leq 0 \vee x_k \geq 1 \\ x_k = 0, \qquad t < t_k \\ x_k = 1/N, \qquad t = t_k \\ t_k = t_{k-1} + \tau_k;\ \tau_k \sim \mathrm{Exp}(\mu) \end{cases} \tag{SE19}$$

Upon introduction, a clone k has its fitness s_k drawn from a gamma distribution $s_k \sim \mathrm{Gamma}\left(\frac{\eta^2}{\sigma^2}, \frac{\sigma^2}{\eta}\right)$ with mean η and variance σ^2. Numerical solution trajectories of this system were calculated using the Julia computing language and the package 'DifferentialEquations.jl' [6–8]. Example solutions of this model are shown in Figure S1.

2.2 Unconstrained growth modeling

An alternative proposed model for CH [9, 10] is one in which there are no constraints on the total number of HSCs, effectively allowing all fit mutant clones to grow exponentially without competition. Because size changes are measured in terms of allele frequencies, growth trajectories would still appear sigmoidal upon measurement [10], suggesting such a model may predict similar empirical observations. However, an important difference is that in such a scenario the total HSC population must growth exponentially along with the expansion of its inhabiting mutant clones. As there currently exists little evidence for such behavior, we investigated to what extent the HSC pool would be expected to expand in a human lifetime under such a model. Constructing a birth-death process and moving to a diffusion picture in a manner analogous to 2.1, it is straightforward to show that the absolute number of cells X_k in a clone obeys the SDE

$$\mathrm{d}X_k = s_k X_k\, \mathrm{d}t + \sqrt{(2\beta + s_k) X_k}\, \mathrm{d}W_t \tag{SE20}$$

with β again the wild type division rate. The observable frequency of a clone can then be found as $X_k/N = X_k/\sum_j X_j$, where $N = \sum_j X_j$ includes X_0, the fixed wild type population size. Using the optimal parameters obtained from ABC inferences (Figure 3F-H) we measured the distribution of total population sizes over time across 1000 simulations (Figure S22). Taking the initial mature HSC pool to house 100,000 cells, we find a large variation of increases in this size across 1000 simulations. Even in the scenario of lowest clone arrival rate ($\mu \approx 3$ per year) the median population increase is 5-fold that of the initial size, with some simulations reaching 100-1000 fold increases. Such drastic changes to the composition of the bone marrow would likely effect other aspects haematopoiesis, a notion which warrants further empirical investigation.

3 Bayesian inference on clonal trajectories to estimate parameter values

The Bayesian inference on the clone trajectories (Fabre et al. [2]) was performed in the following manner. First 40,000 parameter combinations $\{P_i\}$ — hereafter referred to as particles — were drawn randomly from uniform priors (see 3.2) for each of the n parameters to be investigated. Next, for each particle a simulated dataset was generated from the SDE system (see 3.1), from which the variant size-distribution and growth rate $r_{\log}$ (see Equation (SE2)) distribution were measured and compared to those of the donor data using a χ^2 metric. Finally particles were ranked based on the similarity with the Fabre data according to the metric (see below), and only the top 1% of these were retained, resulting in a n-dimensional posterior distribution of the parameter space (Figure S9 and S16). Sensitivity of the inferences to the number of accepted particles was tested by recovering the posterior distribution at 0.1%, 0.5%, and 5% (Figure S12).

3.1 Simulation of a single ABC particle

To construct the simulated dataset for a single particle, 300 realizations of the SDE system were performed, representing a large cohort of virtual donors. Each run was measured at time T, and of the variants exceeding a detection threshold $x_{min} = 0.002$, only up to three were chosen for tracking, to obtain a similar number of total variants as well as variants per donor in the data. Because trajectories in the dataset were captured through targeted sequencing of known driver genes, it is possible that the observed variants would be biased towards higher innate fitness compared to a randomly selected set of drivers, since knowledge of a driver may correlate with its fitness. To account for this we introduced another parameter q describing the probability of an observed driver possessing the highest innate fitness in the system. In concrete terms, after ranking all variants in a simulated individual by their innate fitness, each of the observed variants has a probability q of being chosen in ranked order, and a probability $1 - q$ of being selected randomly. Thus for q=1, observed variants are maximally biased towards high innate fitness — i.e. sampled in order of innate fitness — whereas for q=0 variant observation is entirely random. From the pooled set of selected variants across all simulated individuals, the size distribution was constructed by binning variant sizes at the time of measurement into 25 bins covering the full VAF range $[x_{min}/2, 0.5]$ (the size of a clone is twice its variant allele frequency). For the growth rate distribution, each sampled variant was measured at the four equidistant time points spanning 10 years centered around T: $\{T - 5, T - 1.66, T + 1.66, T + 1.66\}$. The maximum likelihood fitting described in 1 was then performed to obtain all variants' growth rates $r_{\log}$. which were partitioned into 50 equidistant bins constituting the logistic growth rate distribution. Because both the size- and growth rate distributions are expected to change with the age of observed individuals, the donor dataset was partitioned into age groups covering 6 year spans, and the comparison with simulations was performed with T at the centre of the respective age group, for example variants measured between 70-76 years of age were compared with simulations measured at $T = 73$ (Figure S16).

3.2 Prior distribution ranges

The uniform prior distributions — from which each particle's parameter set P_i was drawn — were chosen such that they covered plausible ranges of the parameters based on literature consensus. With N and τ previously jointly estimated at $N\tau \approx$100,000 [3, 4, 9] and active HSC replication rates thought

to be around once per year [11] (or between once every 2 to 20 months [3]), we took $\tau \in (0.1, 10)$ per year and $N \in (25{,}000, 900{,}000)$. Since growth rates of most expanding clones are typically observed between 5% and 20% per year [2, 12], we set $\eta \in (0.01, 0.4)$ and $\sigma \in (0.001, 0.1)$. We also tested whether inferences of η, σ, and μ improved if the population size N and replication rate $1/\tau$ were fixed to current estimates of 100,000 HSCs and once per year. We found this to have little effect on the posteriors (Figure S17). Finally, for the total occurrence rate of driver mutations (or other fitness-granting mechanisms) μ we found that rates below 0.1 per per year often failed to produce detectable clones, whereas those greater than 30 per year became increasingly computationally expensive. For this reason we performed the main analysis with the prior $\mu \in (0.1, 30)$, and performed a sparser second ABC with a wider prior $\mu \in (1, 512)$ to validate the result (Figure S14).

3.3 Testing the ABC convergence with simulated data

To test the ability of the ABC framework to accurately identify unknown parameter values, we performed the inference on 1000 simulated datasets with known parameters. As inferred values we took the modes of the posterior distributions. Taking the Euclidean distance between inferred and true parameter value as the error, we then measured the distribution of errors across all inferences for each parameter (Figure S7B). For all parameters the error distributions have modes near 0 and mean errors less than 1 fold-change.

4 Accumulation of neutral mutations in the multi-type birth-death process

To study site frequency spectra (SFS) we must include the accumulation of neutral mutations in individual haematopoietic stem cells, as these are expected to form the majority of the cell mutational burden. Current evidence suggests mutations can occur both during cell division due to replication errors, as well as in between divisions due to persistent background stresses on the genome (e.g. radiation, oxidation, etc.) and imperfect DNA repair [13]. We must distinguish between these two forms of mutation as they lead to different distributions of the mutational burden in single cells [14]. The number of division-associated mutations in a cell scales with the number of divisions its parental lineage has undergone, whereas the number of background-associated divisions scales directly with time. We take division-associated mutations to be acquired as a Poisson distributed number during each division, and background mutations as a Poisson process in time. To this end we expand upon the events described in Equation (SE5). Denoting $\mathcal{X}_{k,\{u\}}$ a cell of type k with set of mutations $\{u\}$, and U the set of all existing mutations, division-associated mutations are introduced during birth events

$$\begin{cases} \mathcal{X}_{k,\{u\}} \\ \{v\}, \{w\} \notin U : U \end{cases} \xrightarrow{\beta_k} \begin{cases} \mathcal{X}_{k,\{u\}\cup\{v\}} + \mathcal{X}_{k,\{u\}\cup\{w\}} \\ U \cup \{v\} \cup \{w\} \end{cases} \tag{SE21}$$

and background mutations establish the additional event

$$\begin{cases} \mathcal{X}_{k,\{u\}} \\ \{z\} \notin U : U \end{cases} \xrightarrow{\nu} \begin{cases} \mathcal{X}_{k,\{u\}\cup\{z\}} \\ U \cup \{z\} \end{cases} \tag{SE22}$$

4.1 Ontogenic growth of the HSC population

Because population expansion drives neutral mutations to high frequency, the shape of the SFS reflects changes in a population's size [15]. These changes can occur gradually, such that SFS measured from blood in adult humans still contain signatures of the early development of the HSC pool. In order for a model to accurately capture the changing SFS in an individual, it must therefore also incorporate early ontogenic growth [14]. To this end we include an exponential growth phase preceding the previously-described constant population dynamics, starting from a single cell and lasting until the adult size of the HSC pool N is reached, which we take as the time of birth (0 years old). We take this phase to occur as a pure-birth process without cell death.

4.2 Agent-based model with inclusion of neutral mutations

We implemented the clonal competition model with accumulation of neutral mutations as a Moran model [16] via the stochastic simulation algorithm (SSA, also referred to as the Gillespie algorithm [17]). The SSA generates stochastic trajectories of the model by iteratively simulating the events described in Equations (SE5), (SE21), and (SE22) in a system of cells. Both the timing between events and the events themselves are drawn randomly, so that each simulation is a unique realization of the underlying stochastic process. In our model we assume a *pure-birth process* for the ontogenic growth phase: each event describes a cell division and results in the appearance of a new cell in the system. In the constant-population phase we assume *Moran* dynamics: each event consisting of a birth and a simultaneously occurring death, which enforces an equality of the total birth and death rates and thus keeps the population size constant over time. All events are assumed to occur as Poisson processes in time, i.e. having exponentially distributed waiting times. This means that following an event at time t, from Equation (SE21) the time until the next event is given by

$$\Delta t \sim \text{Exponential}\left(\sum_k \beta_k x_k(t)\right) \tag{SE23}$$

with x_k denoting the size of clone k and β_k its birth rate. The clone to which the proliferating cell belongs is then drawn randomly from the weighted set of existing clones, with the likelihood of clone k being selected given by $x_k s_k / \sum_l x_l s_l$.

Repeating this sampling procedure generates a sequence of proliferating cells and inter-division times, representing a random realization of the process described by Equation (SE10) without requiring an analytical solution for $P_n^{(k)}$.

We implemented the SSA in the Rust programming language [18, 19] as an agent-based model (ABM). Each agent represents a single HSC, which carries a set of mutations $\{u\}$ and belongs to a clone k. The proprieties of the agents define the state of the system at certain time, and we are interested in understanding the changes over time of the state of the system. Upon proliferation, a random cell belonging to the k-th clone is selected and assigned $m_b \sim \text{Pois}(\mu_b \Delta t)$ background neutral mutations, where Δt is the difference between the current time and the last time the cell divided. Then a Bernoulli trial with probability of success $\mu \Delta t / N$ is simulated to determine whether to allocate the cell to a new empty clone. The dependence on the stochastic inter-division time decouples the occurrence of fit clones from proliferation events, ensuring that our framework is agnostic to the type of event that generates new clones: they do not acquire a proliferative advantage exclusively via division-associated genetic mutations, but also due to background mutations, epigenetic alterations, or other

environmental factors. When a novel clone is created, its fitness s_k is drawn from a Gamma distribution. This quantifies how fast cells in the clone proliferate according to Equation (SE8). Finally, the dividing cell is duplicated and each daughter cell is assigned a set of random mutations $m_d \sim \text{Pois}(\mu_d)$. If the system has passed the growth phase, a random cell drawn uniformly across all clones is removed from the population. Since we generate clock-like mutational events with the background mutations m_b, we ignore asymmetric divisions for simplicity, which would only increase the mutational burden. Repeating these steps many times allowed us to simulate both clonal dynamics and the accumulation of neutral mutations that act as lineage markers in individual cells.

5 The single-cell HSC dynamics

Having extended the model to incorporate distinct mutations in individual cells, we aimed to understand the dynamics of clonal haematopoiesis (CH) from single-cell resolution data published in Mitchell et al. [4]. We show that using different summary statistics — namely the single-cell mutational burden, the site frequency spectrum and the number of expanded clones — a clonal competition model can quantify underlying evolutionary parameters of CH and recapitulate the genetic heterogeneity observed in the data.

5.1 The dataset of whole genome sequencing of HSC colonies

The HSC whole genome sequencing data published by Mitchell and colleagues [4] consist of samples collected from either the bone marrow, the peripheral or cord blood of 9 healthy donors aged 0 to 81. Cells were sorted and colonies were grown from individual haematopoietic stem cells or multipotent progenitors (HSCs/MPPs) and from haematopoietic progenitor cells (HPCs). For this study we assumed that both HPCs and HSCs/MPPs are subject to the HSC dynamics as differentiated cells in blood show similar mutation loads to HSCs [13]. Colonies were whole-genome sequenced providing high coverage data at single-cell resolution: only clonal colonies were kept for further analysis resulting in 3,579 sequenced colonies, of which 3,361 were grown from HSCs/MPPs and 218 from HPCs. We downloaded the genotype matrices of 9 individuals (3,046 cells) from Mendeley `https://data.mendeley.com/datasets/np54zjkvxr/2` and used them to compute the single-cell mutational burden (that this the number of distinct mutations in individual cells) and the site frequency spectrum. We also stored the number of expanded clones per donor, defined as the number of postnatal ancestral lineages whose descendants contributed more than 1% of colonies at the time of sampling. Note that the information about the clones is extracted from the phylogenies reconstructed by [4] from the single-cell mutations.

5.2 The HSC site frequency spectrum dynamics

We quantified HSC genetic heterogeneity using the site frequency spectrum (SFS) – the distribution of prevalences of all somatic variants within a sample. We analyzed the SFS of the donors to assess whether a clonal competition model can recapitulate the genetic heterogeneity observed in the data.

The SFS were computed from the single-nucleotide variants and short indels present in the individual cells of the 9 donors (216-451 cells per donor, 362 cells on average) stored as the genotype matrices. For

each donor, we calculated the frequency of cells carrying each individual mutation, and then counted all mutations having the same frequencies to obtain the variant counts. We then computed the variant frequencies by normalizing the variant counts, such that the frequencies sum up to 1.

Figure S3 shows the SFS of the donors (orange crosses) along with individual simulations and averages obtained from the ABM model (grey dots and lines). The theoretical expectations of the SFS for expanding populations under neutral dynamics (solid black lines), which follow a $1/f^2$ power-law distribution [15, 20], were sampled to match the sample size of each donor. The theoretical expectations of the SFS for a fixed-size population (dotted black lines) were computed by simulating a birth-death process without selection starting from a first growing phase followed by a fixed-size population phase [14] and were sampled to match the sample size of each donor.

5.3 Model parametrisation with the mutational burden

We aimed to quantify the parameters of the ABM clonal competition model (Supplementary Table 2) from the single-cell data described above. Four parameters were estimated from a Bayesian model (see 5.4), while the others were either fixed from the literature or parametrized using additional information from the data. From current knowledge we set the rate of neutral mutations upon division μ_d to 1.14 [3, 21], and fixed the number of stem cells N to 10^5 cells, based on recent estimates [3, 4, 9, 14] and the fact that the ABC inference performed on clone trajectories displayed limited sensitivity to this parameter within a wide range (Figure S16). Finally, we parametrized the rate of accumulation of neutral background mutations using single-cell mutational burden information from the data (Figure S5A). To accurately capture the dynamics of the burden observed in the data, we assumed different values of this parameter before and after birth, denoted $\mu_{\bar{b}}$ and μ_b respectively.

To compute the prenatal background mutation rate $\mu_{\bar{b}}$, we took mutations to occur as following: an initial growth phase, lasting t_{growth}, has mutations occurring through both divisions and background processes until the maximal population size N is reached; then for the remaining time until birth, that is $t_{\text{birth}} - t_{\text{growth}}$, only background mutations are added. Having the growth process complete before birth ($t_{\text{growth}} < t_{\text{birth}}$) enabled better agreement of the SFS between simulations and data from newborns. We chose t_{growth} such that simulations match the SFS of the neonates in the data, which we found to be 5 months (Figure S3A-B). The value of $\mu_{\bar{b}}$ is then obtained from

$$\tilde{m} = a\mu_d + \mu_{\bar{b}} t_{\text{birth}} \tag{SE24}$$

where $\tilde{m}$ is the expected burden distribution upon birth, t_{birth} is the total time elapsed before birth (9 months), and $a = 2\log\left((N+1)/2\right)$ the expected number of divisions per lineage once the population has reached size N [14]. Taking for $\tilde{m}$ the mean of the burden distributions measured from the two newborns in the data (Figure S5A) resulted in $\mu_{\bar{b}} = 35.64$.

To compute the postnatal background mutation rate μ_b, we regressed the means of the burden distributions for all individuals with no detected clones (i.e. individuals with 0, 29 and 48 years). We found an increase in the average burden of 14.35 mutations per year per cell (Figure S5B), and used as rate $\mu_b = 14.35 - \mu_d/\tau$.

Estimating the mutation rates in this manner ensures that our model accurately captures the mean of the burden in data. However, we find that it underestimates the variance, as similarly reported in previous work [14], with an error that increases with age. This suggests a high heterogeneity across cells in the number of mutations due to other processes not modeled in our framework (Figure S5C-D).

5.4 Bayesian inference on site frequency spectra to estimate parameter values

We then investigated the identifiability of the underlying evolutionary parameters of CH from *in vivo* single-cell mutational data. We first considered the qualitative behavior of the clonal competition model based on values of the arrival rate of advantageous clones μ, the mean η and standard deviation σ of the innate fitness distribution. Faster dynamics can be obtained by higher values of η and μ, and lower values of τ (Figure S2).

To test what combinations of these parameters $\{\tau, \mu, \eta, \sigma\}$ best describe the data, we implemented approximate Bayesian computation (ABC) inference schemes [22]. ABC is a Monte Carlo likelihood-free method that samples different parameter sets and stores those that generate simulations close to the data, according to a similarity metric applied to selected summary statistics (see 5.4.1).

5.4.1 Summary statistics

We implemented ABC by computing two summary statistics: the SFS S and the number of clones C with frequency greater than 1%. We quantified the similarity between the data y and the simulations z by computing the Wasserstein distance for the SFS $w(S_y, S_z)$ and the relative Euclidean distance for the number of detected clones $d(C_z, C_y) = |C_z - C_y|/C_z$. The Wasserstein distance, also called the Earth mover's distance or the optimal transport distance, is a similarity metric between two probability distributions which computes the unsigned average distance between the inverse cumulative distribution of S_z and S_y [23].

5.4.2 Longitudinal inference using the SFS and the number of clones

We performed a longitudinal inference to estimate the values of $\{\tau, \mu, \eta, \sigma\}$ from the single-colony data published in Mitchell et al. [4]. As mentioned above, the data consist in *in vitro* expanded single-cell colonies collected from either the bone marrow, the peripheral or cord blood of 9 healthy donors aged 0 to 81. Since the individuals are healthy donors, we assumed that they followed the same genetic process, i.e. we assumed that the individuals represented timepoints of the same underlying stochastic process. Being interested in the evolution of the CH dynamics over time, we needed exactly one datapoint per age: we kept the newborn with the higher number of cells and discarded the other one, running the inferential task on 8 donors in total.

We generated 65,000 realizations of our agent-based model on the high-performance cluster [24] drawing different sets of parameters from uniform prior distributions (see 3.2):

$$\begin{aligned} \eta &\sim \mathcal{U}_{[0.01,\,0.4]} \\ \sigma &\sim \mathcal{U}_{[0.001,\,0.1]} \\ \tau &\sim \mathcal{U}_{[0.1,\,10]} \\ \mu &\sim \mathcal{U}_{[0.1,\,30]}. \end{aligned} \tag{SE25}$$

We saved the state of the simulations after subsampling the stem cell population to the number of cells found in the 8 donors at their respective ages (age 0, 29, 38, 48, 63, 76, 77, 81). We then quantified the similarity between the simulations z and data y using the two summary statistics described above. We obtained the posterior distributions by storing the parameter sets that generated simulations with

both metric distances within the 1% quantile for at least 7 out of 8 timepoints i, i.e. the parameter sets for which the simulations had $d(C_y^i, C_z^i) < q \vee w(S_y^i, S_z^i) < q$, with q being the 1% quantile. These filtered parameters are approximated samples of the posterior distributions that describe the data best. We summarized the posterior distributions into point estimates by taking their modes after binning them into histograms, reporting the 90% credible interval. The summary statistics for the 8 donors are shown in Figure S6 (see also Figure 3D-E which show the summary statistics for the SFS of the 76 years old donor and the number of clones for all donors). The simulations that minimize $d(C_y^i, C_z^i) < q \vee w(S_y^i, S_z^i) < q$ recapitulate the stochastic behavior of clonal expansions, detected in the SFS as peaks in the higher frequency domain (Figure S11).

The inferred posterior distributions show estimates of mean and standard deviation per year of $\eta = 0.09^{+0.03}_{-0.04}$ and $\sigma = 0.025^{+0.011}_{-0.013}$, and an arrival rate of advantageous variants of $\mu = 3^{+23}_{-0}$ (Figure S10). Taking the point estimates of the posterior distributions of η and σ allows us to plot the Gamma distribution of the fitness effects inferred from the data by mapping η and σ to the scale α and shape θ: $\alpha = \eta^2/\sigma^2$ and $\theta = \sigma^2/\eta$. The recovered distribution of fitness effects is bell-shaped, suggesting that most clones expand at similar rates (Figure 3I). Moreover, we find that fit clones expand slowly, with an average growth that is 9% faster per year compared to wild-type cells. The findings do not depend on the number of runs accepted by the Bayesian framework, the estimates being consistent across a range of accepted runs of 0.1%, 0.5% and 5% (Figure S13).

5.4.3 Testing the Bayesian framework on simulated data

To validate our inferential framework, we tested our implementation on synthetic data generated by the ABM simulations with $\eta = 0.10$, $\sigma = 0.723$, $\mu = 4.86$ and $\tau = 1.16$. We found $\eta = 0.09^{+0.11}_{-0.05}$, $\sigma = 0.055^{+0.02}_{-0.02}$, $\mu = 1^{+23}_{-0}$ and $\tau = 2.5^{+5.9}_{-0.09}$ (Figure S8), confirming the validity of the inferential framework for this parameter range.

6 Combining three longitudinal sequencing datasets

To apply the clonal competition model to a large dataset, we aggregated the target sequencing data generated by Fabre et al. [2], by Robertson et al. [10] and by Van Zeventer et al. [12]. These are three longitudinal studies of DNA blood samples, collected from a total of 1,969 individuals with no history of hematological malignancy, for which a target panel of driver genes was sequenced. Some donors in the Van Zeventer et al. [12] had anemia, cytopenia, or peripheral blood count abnormalities. To ensure consistency across data, variants with a frequency smaller than 1% VAF were removed from Fabre et al. and Van Zeventer et al. A stricter threshold of $< 1.5\%$ VAF was adopted for Robertson et al. , due to excessive noise in this data. In total this resulted in 1,942 viable donors across all data sets.

6.1 The rate of CH in data

Clonal haematopoiesis of indeterminate potential (CHIP) is defined by the presence of at least one expanded clone with VAF $\geq 2\%$ [25]. To estimate the prevalence of CHIP in the data, we stored the donors' age at which the first variant with a frequency greater than 2% VAF occurred. These ages were binned into two-year spans, generating a distribution of ages (Figure S19A). Then we took the

cumulative sum of this distribution and normalised it by the total number of donors (with and without CHIP) to obtain the prevalence. We found 727 out of 1,942 total donors (37%) without any evidence of CHIP (Figure S19B, light blue), possibly underestimated by some missed clones. Half of the donors had CHIP by 75 years of age, showing that CHIP is prominent in healthy individuals. The prevalence of CHIP correlates with age (dark blue), as expected, and shows three different regimes: initially, it increases linearly, then sublinearly, and finally stays constant in the elderly. As most of the datapoints lie between 60-92 years (Figure S19A), the initial linear phase and the constant final phase might be biased by a small sample size.

To mitigate the age sampling bias in estimating the prevalence if CHIP, we computed the rate of CHIP incidence. Unlike the prevalence of CHIP, which is calculated by dividing the cumulative age distribution by the total number of donors, here we normalized the number of donors with CHIP within two-year age bins by the total number of donors in each bin. The estimated CHIP rate peaks around 60 years and declines at older ages (Figure S19C). This observed peak is likely influenced by age bias in the dataset, as very few data points were collected from individuals younger than 60 years. If blood samples from younger donors had been available, a more gradual increase between 55 and 60 years would likely have been observed. Additionally, it is possible that targeted sequencing approaches missed smaller clones that had already expanded at an earlier stage. Finally, the decrease in CHIP rate can be explained by the survival bias present in this dataset of healthy individuals, as cancer patients, who usually show large clones at younger ages, were excluded.

Plotting the prevalence of some frequently mutated genes showed that mutations were common in epigenetic regulator genes *DNMT3A* and *TET2*, and also frequent in *ASXL1*, *TP53*, *PPM1D* (Figure S19D). Figures S19E-G show the changes in VAF over time for *JAK2*-V617F, *DNMT3A*-R882H and *DNMT3A*-R882H, where each line corresponds to a donor (trajectory). *JAK2*-V617F was the most prevalent variant found in 67 donors, followed by *DNMT3A*-R882H, found in 49 donors, and *SRSF2*-P95H, found in 26 donors. Sequencing only 79 genes already shows a complex fitness landscape according to which trajectories can stay constant, increase or decrease over time. This suggests a dynamic fitness landscape that can be recapitulated by the clonal competition model, as opposed to simpler models that fail to do so. Interestingly, the data show that the same variant can have different growth effects in donors, suggesting that the fitness advantage conferred by the variant depends on the context in which it occurs, influenced by the presence of other clones, the microenvironment or other epigenetic factors.

6.2 The number of detectable clones in data and simulations

Stratifying donors into age groups, we examine the number of detectable clones in individuals of a certain age at a sequencing threshold of 1%VAF (Figure 3K). We find the distribution of clone numbers to widen with age up to around 75, with the mean number of clones similarly increasing. We compare this to simulations of the SDE model (1) using parameters obtain by resampling the posteriors found in the ABC (see Figures 3F-H, S9, S10, and 3, 5.4), where we count all clones above the threshold at each age. These showed typically higher clone numbers and a wider variance, though with a similar age-dependent trend (Figure 3K). The distribution of the expected number of missing clones (Figure 3L) was found by taking the difference of the mean clone number in simulations and each donor in the data.

Intuitively, the number of detectable clones should depend heavily on the occurrence rate of fit clones μ. Since this parameter was estimated with the least confidence in our ABC (Figure 3H), we investigated

the robustness of the above result by performing multiple sets of simulations for different μ (Figure S20). We found that the number of detectable clones indeed increases with higher μ, however the saturation (or even slight decrease) of the clone number in the latest stage of life remains consistent across all μ. Furthermore, from $\mu > 25$ the rise in clone number becomes increasingly less significant, with a clear saturation by $\mu = 100$. This suggests that even without any knowledge of μ, the expected number of missing (i.e. undetected) clones is capped around 7.

7 Bayesian inference of innate fitness and arrival time on individual trajectories

To estimate the innate fitness $\tilde{s}$ and arrival time $\tilde{t_0}$ of the trajectories we performed an additional approximate Bayesian computation. This compared a target trajectory to an ensemble of trajectories simulated from the SDE model (1), with the best-fit parameters obtained from the ABC described in 3. Each simulated trajectory was generated by inserting one single-celled clone with a predetermined fitness s_i at an arrival time t_i in a simulation of the SDE and tracking it over time. Drawing all s_i and t_i from uniform prior distributions $s \sim U(0.01, 2)$ and $t \sim U(0, 90)$, we obtained an ensemble of 150,000 independently simulated trajectories. For a target trajectory $\mathbf{x} = \{\tilde{x}_j\}$ measured at ages $\{z_j\}$, the distance from a reference trajectory $x_i(t)$ was calculated as the Euclidean distance $d_i = |\tilde{\mathbf{x}} - \mathbf{x_i}|$ from the reference measured at the same times $\mathbf{x_i} = \{x_i(z_j)\}$. To generate the posterior distribution we retained only trajectories with distances up to the 150th order statistic (0.1% of all trajectories). Finally, to obtain estimators for $\tilde{s}$ and $\tilde{t_0}$ we performed a kernel density estimate of the marginalized posteriors and took their maxima (Figure 4B).

7.1 Estimation error arises from inherent stochasticity

To probe the accuracy of this method we generated ensembles of target trajectories with a fixed parameter, for example a fixed $\tilde{s}$ and random $\tilde{t_0}$, or vice versa. We then performed the ABC comparison on each trajectory in the simulated ensemble, resulting in a distribution of estimates for the fixed parameter (4C). This distribution encodes the inherent uncertainty of the inference method, which arises from the randomness of the evolutionary process itself. Indeed, without specific knowledge of competing variant behaviors, a variant originating at t_0 with fitness s has a distribution of possible future trajectories, all of which have a certain likelihood of occurring. By running many realizations of the process, the ABC reproduces this distribution for all possible (t_0, s) combinations. Upon comparison with a target trajectory, it identifies the (t_0, s) pair with the most similar expected trajectory. Thus variants which evolve according to likely scenarios (i.e. close to their expected trajectory) will have trajectories similar to mean trajectories from nearby points in parameter space. However a variant which ends up on an unlikely trajectory, far from its expected value, will be identified as closer to a more distant parameter pair (t_0', s'). Overall, we found the errors to be unbiased for wide ranges of s and t_0, excepting very early arrival times ($t_0 \approx 0$), which had a slight positive bias. The error distributions also showed slight skewing depending on the parameter value, and increasing variance for higher fitnesses and lower arrival times.

7.2 Genic variant fitness estimates from multiple donors

To estimate the innate fitness of a particular variant observed in multiple donors, we note that a single trajectory fitness estimate $\hat{s}$ is subject to noise arising from both the above-described error, as well all other perturbing factors. These could be measurement errors, or unmodeled biological processes such as individual genetic variability or external perturbations (smoking, diet, etc). We thus write

$$\hat{s} = s + e_c + e_x \tag{SE26}$$

where s is the variant's true innate fitness and we have split the noise into a contribution from the Bayesian estimation e_c due to stochastic competition (see 7.1) and a contribution from all other sources e_x. Since e_c is unbiased, and assuming e_x is as well, we have $\langle \hat{s} \rangle = \langle s + e_c + e_x \rangle = s$. Therefore we can take the mean of the trajectory estimates within a single variant as estimator for the true innate fitness of that variant. For the most frequently occurring variants ($n > 10$) we obtained the fitness estimates shown in Supplementary Table 1. Each estimate has a standard error that depends on the number of included trajectories n as $\mathrm{SE}(n) = \sigma_{\hat{s}}/\sqrt{n}$, where $\sigma_{\hat{s}}$ is the standard deviation of the distribution of individual estimates. This can be found from the variance, which — further assuming the errors are not correlated — takes the form

$$\sigma_{\hat{s}}^2 = \left\langle e_c^2 \right\rangle + \left\langle e_x^2 \right\rangle \tag{SE27}$$

Here $\left\langle e_x^2 \right\rangle$ is by definition unknown, as it describes all error contributions that were not modeled. We can however obtain $\langle e_c \rangle^2 := \sigma_c^2$ — the error from our Bayesian estimate — by performing the Bayesian inference on a large set of simulated trajectories with identical innate fitness. In these simulations $e_x = 0$, thus taking the variance of the fitness estimates directly gives $\sigma_{\hat{s},\mathrm{sims}}^2 = \left\langle e_c^2 \right\rangle = \sigma_c^2$. For an innate fitness of $s = 0.15$ this resulted in $\sigma_c = 0.0267$ (Figure 4G).

With the value of σ_c^2 known, any remainder of variance observed in real-world trajectories of identical mutations must be due to unmodeled external factors. Or in other words, any variance up to σ_c^2 can be explained by competition — the cause of the error in our Bayesian estimate. To estimate how much would remain unexplained we looked to the data. We considered each set of fitness estimates per variant $\{s_i\}_{\mathrm{var}}$ as a collection of independent samples of $\hat{s}_{\mathrm{var}}$. Since we suspect a subset of observed trajectories to have undergone additional fitness-granting events (see Section 8.2), we excluded those categorized as potentially multi-hit-driven. Within this set, we interpret the respective observed variances $\bar{\sigma}_{\hat{s},\mathrm{var}}^2$ as independent estimators of $\sigma_{\hat{s}}^2$. Of the variants considered (> 10 occurrences in the donor data), DNMT3A_p.R882H/C and JAK2_p.V617F were the highest recurring, with n=47 and n=31 respectively, compared to n=13 for the next highest. Since their variances $\bar{\sigma}_{p.R882H/C}^2$ and $\bar{\sigma}_{p.V617F}^2$ should have the lowest statistical error with regards to the true $\sigma_{\hat{s}}^2$ we took a weighted mean of these as estimator $\bar{\sigma}_{\hat{s}}^2$. Under an assumption of Gaussian errors, the 95% confidence interval for this estimate is [26]:

$$\left(\sigma_{\hat{s},LB}^2, \sigma_{\hat{s},UB}^2\right) = \left(\frac{(n-1)\bar{\sigma}_{\hat{s}}^2}{\chi_{0.975,n-1}^2}, \frac{(n-1)\bar{\sigma}_{\hat{s}}^2}{\chi_{0.025,n-1}^2}\right) \tag{SE28}$$

where $\chi_{p,k}$ is the p-quantile of the chi-squared distribution with k degrees of freedom. We then estimate a minimal value for $\sigma_c^2/\sigma_{\hat{s}}^2$ — the proportion of total variance explained by competition — by taking $\sigma_c^2/\sigma_{\bar{s},UB}^2$.

8 The arrival time distribution of detectable variants

In order to evaluate the distribution of arrival times estimated from donor variant trajectories we investigate model predictions for this quantity.

8.1 Single-event clones

We first consider the case of variants arriving randomly in time according to the Poisson process, consistent with the observation of linearly accumulating mutations in the population. Denote $S_T(t)$ the expected number of detectable variants measured at time T which originated at time t. To obtain an expression for this we consider the following simplifications of the previously described model (2):

- Deterministic dynamics: newly arising clones grow according to a fixed growth function, i.e. experience no fluctuations and do not go extinct.
- Exponentially growing clones: we take clones to grow independent of any compartmental or competitive limitations, as we are only interested in the early growth period, until a clone reaches the detection threshold.

Under these assumptions, with μ the arrival rate of new clones and η and σ^2 the mean and variance of the (Gamma-distributed) fitness distribution, the expected number of detectable variants is given by:

$$S_T(t) = \mu \frac{(T-t)\Gamma\left(\frac{\eta^2}{\sigma^2}, \frac{R\eta}{(T-t)\sigma^2}\right) - R\frac{\eta}{\sigma^2}\Gamma\left(\frac{\eta^2}{\sigma^2} - 1, \frac{R\eta}{(T-t)\sigma^2}\right)}{\Gamma\left(\frac{\eta^2}{\sigma^2}\right)} \tag{SE29}$$

with $R = \log(Z/n_0)$ where Z is the size threshold for detection and n_0 is the starting size of newly initiated clone.

To derive this we first write $S_T(t)$ as an integration over time and fitness:

$$S_T(t) = \int_0^\infty \mathrm{d}s \int_{t+\zeta(s)}^T \mathrm{d}\tau \, \mu f(s) \Theta[T - (t + \zeta(s))] \tag{SE30}$$

where $\Theta(x)$ is the Heaviside step function, and $\zeta(s)$ is the time required for a clone to grow from its inception size n_0 to the detection threshold Z. This expression essentially states that the total number of detectable clones can be found by integrating over contributions from clones at all fitnesses. The contribution from clones at a particular fitness is found by integrating the constant arrival rate over the timespan from when they would first become detectable (given their arrival at time t) until the measurement time. The step function appears because we must only count clones that can actually reach the detection threshold within the given time frame $T - t$. Under deterministic non-competitive dynamics clones grow exponentially, so that $\zeta(s) = \log(Z/n_0)/s = R/s$. The inner integral can be performed immediately. The step function can be absorbed into the lower bound of the second integral (over s) with the assumption that $\zeta(s) < T - t$. Plugging in the probability density function of the

Gamma distribution with shape α and scale θ we are then left with

$$S_T(t) = \mu \int_{\frac{R}{T-t}}^{\infty} \frac{1}{\Gamma(\alpha)\theta^{\alpha}} s^{\alpha-1} e^{-s/\theta} \left[T - t - R/s\right] \mathrm{d}s \tag{SE31}$$

$$= \frac{\mu}{\Gamma(\alpha)\theta^{\alpha}} \left[(T-t)\theta^{\alpha} \int_{\frac{R}{(T-t)\theta}}^{\infty} u^{\alpha-1} e^{-u} \, \mathrm{d}u - R\theta^{\alpha-1} \int_{\frac{R}{(T-t)\theta}}^{\infty} u^{\alpha-2} e^{-u} \, \mathrm{d}u \right] \tag{SE32}$$

Where in the second equality we have performed the integral substitution $u = s/\theta$. Here we recognize the incomplete gamma function

$$\Gamma(\alpha, z) = \int_{z}^{\infty} u^{\alpha-1} e^{-u} \, \mathrm{d}u \tag{SE33}$$

in the first term and thus $\Gamma(\alpha - 1, R/(T-t)\theta)$ in the second. Finally, substituting shape and scale for mean and variance through the relations $\alpha = \eta^2/\sigma^2$ and $\theta = \sigma^2/\eta$ we obtain the result in Equation (SE29).

8.2 Multiple-event clones

To obtain a prediction for the arrival time distribution of clones originating with epistatic interactions, e.g. a second or third mutation, we modified our simulations (see 2.1) to include compounding fitness effects in the following manner. For each newly arising fit variant, a parent clone is chosen randomly from all currently existing clones in the system. The likelihood of a parent being selected is given by its population fraction. If the parent is the wild type, then the clone receives an innate fitness randomly drawn from the fitness distribution, just as in the non-epistatic model. If the parent is an expanding driver, the new clone receives a fitness that is a combination of the parent's innate fitness and a value drawn from the fitness distribution. We used a multiplicative fitness to model the epistatic effect of multiple variants:

$$s_{\text{child}} = s_{\text{parent}} + s_{\text{draw}} + s_{\text{parent}} s_{\text{draw}} \tag{SE34}$$

where s_{child} is the innate fitness of the newly arising clone, s_{parent} the innate fitness of the parent, and s_{draw} the fitness granted by the novel mutation.

We then ran a large number of simulations (50,000) and recorded the arrival times of all detectable clones (threshold 0.01%) with at least two drivers measured at random times between ages 60 and 95, resulting in the age distribution shown in Figure 4.

8.3 Comparing data and simulations

On the premise that clones carrying multiple fit mutations (multi-step clones) have higher fitness compared to clones carrying only one fit mutation (single-event clones), we hypothesized that the fitness distribution inferred from the trajectory data is due to a mixture of low and high fitness clones, corresponding to single- or multi-event drivers respectively. To find the fitness threshold s_T that distinguishes between these ($s_{low} < s_T < s_{high}$), we performed unsupervised clustering assuming that a Gaussian mixture model with two components could recapitulate the underlying distributions of the inferred fitness values. This approach showed that at $s = 0.226$ the probability for a fitness value to be assigned to either cluster was the same, and thus set $s_T = 0.226$ to discriminate between low and high fitness clones (Figure 4D).

To quantitatively assess the agreement between arrival time distributions obtained from data and simulations we made use of the k-sample Anderson-Darling test [27] and the two-sample Kolmogorov-Smirnov test. Both are non-parametric statistical tests that evaluate whether two (or k) sets of observations could be sampled from the same (unknown) probability distribution. Both test the null-hypothesis that the samples are drawn from the same distribution, thus similarity of the samples results in a high p-value and failure to reject. Taking the above-described clustering based on clone fitness, we find the arrival-time distribution of high fitness clones ($s > 0.226$) to match the prediction of multi-step clones from simulations (as shown in Figure 4E), with both statistical tests failing to reject the null: $p_{KS} = 0.27$ (Kolmogorov-Smirnov) and $p_{AD} = 0.09$ (Anderson-Darling). To further probe the stability of this result, we calculated the p-value of both statistics for a range of s_T, as shown in Figure S21. The p-values of both tests peak at $s_T = 0.22$, with maxima $p = 0.71$ (AD) and $p = 0.87$ (KS), and show clear rejection a short distance away. This demonstrates a significant failure to reject the null within a small window of s_T, and solidifies the statistical similarity between the observed high fitness trajectories and simulated multi-step clones.

This statistical agreement does not hold when comparing arrival times of the lower fitness variants to the expectation of single-step variants. This may be explained in part by the error on our arrival time estimates. When measuring this error from simulations (Figure 4C) we note that while the estimation error is symmetric for arrival times in adulthood, it appears to be positively biased for variants occurring early in life. This bias will have pushed the peak of the distribution from birth to childhood, which visually can be seen to form the main difference between theory and data.

References

[1] Peter Czuppon and Arne Traulsen. "Understanding evolutionary and ecological dynamics using a continuum limit". In: *Ecology and Evolution* 11.11 (2021), pp. 5857–5873.

[2] Margarete A Fabre et al. "The longitudinal dynamics and natural history of clonal haematopoiesis". In: *Nature* 606.7913 (June 2022), pp. 335–342.

[3] Henry Lee-Six et al. "Population dynamics of normal human blood inferred from somatic mutations". In: *Nature* 561.7724 (Sept. 2018), pp. 473–478.

[4] Emily Mitchell et al. "Clonal dynamics of haematopoiesis across the human lifespan". In: *Nature* 606.7913 (June 2022), pp. 343–350.

[5] Hannes Risken. *The Fokker-Planck Equation: Methods of Solution and Applications*. 2nd ed. Springer, 1996. Chap. 4.5.

[6] Christopher Rackauckas and Qing Nie. "Differentialequations.jl–a performant and feature-rich ecosystem for solving differential equations in julia". In: *Journal of Open Research Software* 5.1 (2017), p. 15.

[7] Christopher Rackauckas and Qing Nie. "Adaptive methods for stochastic differential equations via natural embeddings and rejection sampling with memory". In: *Discrete and continuous dynamical systems. Series B* 22.7 (2017), p. 2731.

[8] Chris Rackauckas and Qing Nie. "Stability-optimized high order methods and stiffness detection for pathwise stiff stochastic differential equations". In: *2020 IEEE High Performance Extreme Computing Conference (HPEC)*. IEEE. 2020, pp. 1–8.

[9] Caroline J Watson et al. "The evolutionary dynamics and fitness landscape of clonal hematopoiesis". In: *Science* 367.6485 (Mar. 2020), pp. 1449–1454.

[10] Neil A Robertson et al. “Longitudinal dynamics of clonal hematopoiesis identifies gene-specific fitness effects”. In: *Nature medicine* 28.7 (2022), pp. 1439–1446.

[11] Sandra N Catlin et al. “The replication rate of human hematopoietic stem cells in vivo”. In: *Blood, The Journal of the American Society of Hematology* 117.17 (2011), pp. 4460–4466.

[12] Isabelle A van Zeventer et al. “Evolutionary landscape of clonal hematopoiesis in 3,359 individuals from the general population”. In: *Cancer Cell* 41.6 (June 2023), 1017–1031.e4.

[13] Federico Abascal et al. “Somatic mutation landscapes at single-molecule resolution”. In: *Nature* 593.7859 (May 2021), pp. 405–410.

[14] Marius E Moeller et al. “Measures of genetic diversification in somatic tissues at bulk and single-cell resolution”. In: *eLife* 12 (2024), RP89780.

[15] Einar Bjarki Gunnarsson, Kevin Leder, and Jasmine Foo. “Exact site frequency spectra of neutrally evolving tumors: A transition between power laws reveals a signature of cell viability”. In: *Theor. Popul. Biol.* 142 (Dec. 2021), pp. 67–90.

[16] Warren John Ewens. *Mathematical population genetics: theoretical introduction*. Vol. 1. Springer, 2004.

[17] Daniel T. Gillespie. “Stochastic Simulation of Chemical Kinetics”. In: *Annu. Rev. Phys. Chem.* 58 (2007), pp. 35–55.

[18] Nicholas D. Matsakis and Felix S. Klock. “The Rust Language”. In: *ACM SIGAda Ada Letters* 34 (Oct. 2014), pp. 103–104. ISSN: 1094-3641. DOI: 10.1145/2692956.2663188.

[19] Jeffrey M. Perkel. “Why Scientists Are Turning to Rust”. In: *Nature* 588 (Dec. 2020), pp. 185–186. ISSN: 1476-4687. DOI: 10.1038/d41586-020-03382-2.

[20] Rick Durrett. “Population Genetics of Neutral Mutations in Exponentially Growing Cancer Cell Populations”. In: *The Annals of Applied Probability* 23 (Feb. 2013), pp. 230–250. ISSN: 1050-5164. DOI: 10.1214/11-AAP824.

[21] Benjamin Werner et al. “Measuring single cell divisions in human tissues from multi-region sequencing data”. In: *Nat. Commun.* 11.1 (Feb. 2020), p. 1035.

[22] Mark A. Beaumont. “Approximate Bayesian Computation in Evolution and Ecology”. In: *Annual Review of Ecology, Evolution, and Systematics* 41 (2010), pp. 379–406. DOI: 10.1146/annurev-ecolsys-102209-144621.

[23] Aaditya Ramdas, Nicolas Garcia, and Marco Cuturi. *On Wasserstein Two Sample Testing and Related Families of Nonparametric Tests*. Oct. 2015. DOI: 10.48550/arXiv.1509.02237. arXiv: 1509.02237 [math, stat].

[24] Thomas King, Simon Butcher, and Lukasz Zalewski. “Apocrita - High Performance Computing Cluster for Queen Mary University of London”. In: (Mar. 2017). DOI: 10.5281/zenodo.438045.

[25] Siddhartha Jaiswal and Benjamin L Ebert. “Clonal hematopoiesis in human aging and disease”. In: *Science* 366.6465 (Nov. 2019), eaan4673.

[26] George Casella and Roger Berger. *Statistical inference*. Chapman and Hall/CRC, 2024. Chap. 5.3.1.

[27] Fritz W Scholz and Michael A Stephens. “K-sample Anderson–Darling tests”. In: *Journal of the American Statistical Association* 82.399 (1987), pp. 918–924.
