# Supplementary material for: The Evolution of Polyclonal Competition in Aging Hematopoiesis
Source: Cancer Discov. 2026 May 5;16(9):1765–79. doi: 10.1158/2159-8290.CD-25-0990 (PMC13531000; doi:10.1158/2159-8290.CD-25-0990)
Supplement: Supplementary file 3 [file cd-25-0990_supplementary_table_1_suppst1.pdf]

Variant	Trajectories	s_{innate}	2SE
DNMT3A_p.P904L	13	0.14	0.016
DNMT3A_p.Y735C	13	0.14	0.016
DNMT3A_p.R882H/C	47	0.15	0.0085
TET2_p.I1873T	12	0.15	0.017
JAK2_p.V617F	31	0.16	0.010
ASXL1_p.G646fs	12	0.16	0.017
SF3B1_p.K700E	9	0.14	0.019
SF3B1_p.K666N	10	0.17	0.018
SRSF2_p.P95H13	8	0.18	0.021

Supplementary Table 1: Inferred innate fitness of commonly occurring variants.
