# Supplementary material for: The Evolution of Polyclonal Competition in Aging Hematopoiesis
Source: Cancer Discov. 2026 May 5;16(9):1765–79. doi: 10.1158/2159-8290.CD-25-0990 (PMC13531000; doi:10.1158/2159-8290.CD-25-0990)
Supplement: Supplementary file 4 [file cd-25-0990_supplementary_table_2_suppst2.pdf]

| Symbol | Description | Units | Inference |
| --- | --- | --- | --- |
| $N$ | Number of haematopoietic stem cells at maturity | cell number | Fixed to $10^5$ |
| $\tau$ | Inter-division time for wild-type cells | year | Inferred |
| $\eta$ | Mean of the Gamma distribution of innate fitness coefficients | year$^{-1}$ | Inferred |
| $\sigma$ | Standard deviation of the Gamma distribution of innate fitness coefficients | year$^{-1}$ | Inferred |
| $\mu$ | Arrival rate of advantageous mutations | year$^{-1}$ | Inferred |
| $\mu_{\bar{b}}$ | Arrival rate of prenatal neutral background mutations | year$^{-1}$ | Parametrized to 35.64 |
| $\mu_b$ | Arrival rate of postnatal neutral background mutations | year$^{-1}$ | Parametrized to $\mu_b = 14.35 - \mu_d/\tau$ |
| $\mu_d$ | Arrival rate of neutral mutations upon division | division$^{-1}$ | Fixed to 1.14 |

Supplementary Table 2: Parameters of the agent-based model. Parameters were inferred either through the ABC framework or by regression of the mean of the number of mutations.
